# Ancient DNA from European Early Neolithic Farmers Reveals Their Near Eastern Affinities

**DOI:** 10.1371/journal.pbio.1000536

**Published:** 2010-11-09

**Authors:** Wolfgang Haak, Oleg Balanovsky, Juan J. Sanchez, Sergey Koshel, Valery Zaporozhchenko, Christina J. Adler, Clio S. I. Der Sarkissian, Guido Brandt, Carolin Schwarz, Nicole Nicklisch, Veit Dresely, Barbara Fritsch, Elena Balanovska, Richard Villems, Harald Meller, Kurt W. Alt, Alan Cooper, the Genographic Consortium

**Affiliations:** 1 Australian Centre for Ancient DNA, School of Earth and Environmental Sciences, University of Adelaide, Adelaide, Australia; 2 Research Centre for Medical Genetics, Russian Academy of Medical Sciences, Moscow, Russia; 3 National Institute of Toxicology and Forensic Sciences, Canary Islands Delegation, Campus de Ciencias de la Salud, La Laguna, Tenerife, Spain; 4 Faculty of Geography, Moscow State University, Moscow, Russia; 5 Research Centre for Drug Evaluation, Ministry of Public Health of the Russian Federation, Moscow, Russia; 6 Institute for Anthropology, Johannes Gutenberg University of Mainz, Mainz, Germany; 7 Landesamt für Denkmalpflege und Archaeologie und Landesmuseum für Vorgeschichte, Halle (Saale), Germany; 8 Department of Evolutionary Biology, Institute of Molecular and Cell Biology, University of Tartu and Estonian Biocentre, Tartu, Estonia; Massey University, New Zealand

## Abstract

In Europe, the Neolithic transition (8,000–4,000 b.c.) from hunting and gathering to agricultural communities was one of the most important demographic events since the initial peopling of Europe by anatomically modern humans in the Upper Paleolithic (40,000 b.c.). However, the nature and speed of this transition is a matter of continuing scientific debate in archaeology, anthropology, and human population genetics. To date, inferences about the genetic make up of past populations have mostly been drawn from studies of modern-day Eurasian populations, but increasingly ancient DNA studies offer a direct view of the genetic past. We genetically characterized a population of the earliest farming culture in Central Europe, the Linear Pottery Culture (LBK; 5,500–4,900 calibrated b.c.) and used comprehensive phylogeographic and population genetic analyses to locate its origins within the broader Eurasian region, and to trace potential dispersal routes into Europe. We cloned and sequenced the mitochondrial hypervariable segment I and designed two powerful SNP multiplex PCR systems to generate new mitochondrial and Y-chromosomal data from 21 individuals from a complete LBK graveyard at Derenburg Meerenstieg II in Germany. These results considerably extend the available genetic dataset for the LBK (*n* = 42) and permit the first detailed genetic analysis of the earliest Neolithic culture in Central Europe (5,500–4,900 calibrated b.c.). We characterized the Neolithic mitochondrial DNA sequence diversity and geographical affinities of the early farmers using a large database of extant Western Eurasian populations (*n* = 23,394) and a wide range of population genetic analyses including shared haplotype analyses, principal component analyses, multidimensional scaling, geographic mapping of genetic distances, and Bayesian Serial Simcoal analyses. The results reveal that the LBK population shared an affinity with the modern-day Near East and Anatolia, supporting a major genetic input from this area during the advent of farming in Europe. However, the LBK population also showed unique genetic features including a clearly distinct distribution of mitochondrial haplogroup frequencies, confirming that major demographic events continued to take place in Europe after the early Neolithic.

## Abstract 2

The first farmers from Central Europe reveal a genetic affinity to modern-day populations from the Near East and Anatolia, which suggests a significant demographic input from this area during the early Neolithic.

## Author Summary

The transition from a hunter–gatherer existence to a sedentary farming-based lifestyle has had key consequences for human groups around the world and has profoundly shaped human societies. Originating in the Near East around 11,000 y ago, an agricultural lifestyle subsequently spread across Europe during the New Stone Age (Neolithic). Whether it was mediated by incoming farmers or driven by the transmission of innovative ideas and techniques remains a subject of continuing debate in archaeology, anthropology, and human population genetics. Ancient DNA from the earliest farmers can provide a direct view of the genetic diversity of these populations in the earliest Neolithic. Here, we compare Neolithic haplogroups and their diversity to a large database of extant European and Eurasian populations. We identified Neolithic haplotypes that left clear traces in modern populations, and the data suggest a route for the migrating farmers that extends from the Near East and Anatolia into Central Europe. When compared to indigenous hunter–gatherer populations, the unique and characteristic genetic signature of the early farmers suggests a significant demographic input from the Near East during the onset of farming in Europe.

## Introduction

The transition from a hunter–gatherer existence to a “Neolithic lifestyle,” which was characterized by increasing sedentarism and the domestication of animals and plants, has profoundly altered human societies around the world [1],[2]. In Europe, archaeological and population genetic views of the spread of this event from the Near East have traditionally been divided into two contrasting positions. Most researchers have interpreted the Neolithic transition as a period of substantial demographic flux (demic diffusion) potentially involving large-scale expansions of farming populations from the Near East, which are expected to have left a detectable genetic footprint [3],[4]. The alternative view (cultural diffusion model; e.g., [5]) suggests that indigenous Mesolithic hunter–gatherer groups instead adopted new subsistence strategies with relatively little, or no, genetic influence from groups originating in the Near East.

Genetic studies using mitochondrial DNA (mtDNA) and Y-chromosomal data from modern populations have generated contradictory results, and as a consequence, the extent of the Neolithic contribution to the gene pool of modern-day Europeans is still actively debated [6]–[8]. Studies that suggest that the genetic variation in modern-day Europe largely reflects farming communities of the Early Neolithic period [9]–[11] contrast strongly with others that consider the input from the Near East an event of minor importance and ascribe the European genetic variation and its distribution patterns to the initial peopling of Europe by anatomically modern humans in the Upper Paleolithic [12]–[15]. These patterns are also likely to have been significantly impacted by the early Holocene re-expansions of populations out of southerly refugia formed during the Last Glacial Maximum (∼25,000 y ago) and by the numerous demographic events that have taken place in post-Neolithic Europe.

The genetics of prehistoric populations in Europe remain poorly understood, restricting real-time insights into the process of the Neolithic transition [16]–[21]. As a result, most attempts to reconstruct history have been limited to extrapolation from allele frequencies and/or coalescent ages of mitochondrial and Y chromosome haplogroups (hgs) in modern populations. Ancient DNA (aDNA) analyses now provide a powerful new means to directly investigate the genetic patterns of the early Neolithic period, although contamination of specimens with modern DNA remains a major methodical challenge [22].

A previous genetic study of 24 individuals from the early Neolithic Linear Pottery Culture (LBK; 5,500–4,900 calibrated b.c. [cal b.c.]) in Central Europe detected a high frequency of the currently rare mtDNA hg N1a, and proposed this as a characteristic genetic signature of the Early Neolithic farming population [19]. This idea was recently supported by the absence of this particular lineage (and other now more common European hgs) among sequences retrieved from neighboring Mesolithic populations [20],[21]. However, a study of 11 individuals from a Middle/Late Neolithic site on the Iberian Peninsula (3,500–3,000 cal b.c.) did not find significant differences from modern populations, supporting a quite different population genetic model for the Neolithic transition in Iberia [18].

To gain direct insight into the genetic structure of a population at the advent of farming in Central Europe we analyzed a complete graveyard from the Early Neolithic LBK site at Derenburg Meerenstieg II (Harzkreis, Saxony-Anhalt) in Germany. The archaeological culture of the LBK had its roots in the Transdanubian part of the Carpathian Basin in modern-day Hungary approximately 7,500–8,000 y ago and spread during the subsequent five centuries across a vast area ranging from the Paris Basin to the Ukraine [23],[24]. The graveyard samples provide a unique view of a local, closed population and permit comparisons with other specimens of the LBK archaeological culture (the contemporaneous meta-population) and with modern populations from the same geographical area (covering the former range of the LBK), as well as groups across the wider context of Western Eurasia. Our primary aim was to genetically characterize the LBK early farming population: by applying comprehensive phylogeographic and population genetic analyses we were able to locate its origins within the broader Eurasian region, and to trace its potential dispersal routes into Europe.

## Results/Discussion

We used standard approaches to clone and sequence the mitochondrial hypervariable segment I (HVS-I) and applied quantitative real-time PCR (qPCR) as an additional quality control. In addition, we developed two new multiplex typing assays to simultaneously analyze important single nucleotide polymorphisms (SNPs) within the mtDNA coding region (22 SNPs: GenoCoRe22) and also the Y chromosome (25 SNPs: GenoY25). In addition to minimizing the risk of contamination, the very short DNA fragments (average 60–80 bp) required by this approach maximize the number of specimens that can be genetically typed.

We successfully typed 17 individuals for mtDNA, which together with a previous study [19] provided data for 22 individuals from the Derenburg graveyard (71% of all samples collected for genetic analysis; Tables 1 and S1), and significantly extended the genetic dataset of the LBK (*n* = 42), to our knowledge the largest Neolithic database available. Sequences have been deposited in GenBank (http://www.ncbi.nlm.nih.gov/genbank/; accession numbers HM009339–HM009341, HM009343–HM009355, and HM009358), and detailed alignments of all HVS-I clone sequences from Derenburg are shown in Dataset S1.

**Table 1:** Summary of archaeological, genetic, and radiocarbon data.

Sample	Feature	Grave	Age, Sex^a^	Radiocarbon Date (Laboratory Code) (Uncalibrated BP, Cal b.c.) [73]	HVS-I Sequence (np 15997–16409), Minus np 16000	Hg HVS-I	Hg GenoCoRe22	Hg GenoY25
deb09	420	9	Adult, f		rCRS	H	H	
deb06	421	10	Adult/mature, n.d.		Ambiguous	n.d.	H	—
deb11	569	16	Adult, f?		n.d.	n.d.	T	
deb10	566	17	Adult, m		093C, 224C, 311C	K	K	—
deb23	565	18	Infans I, m?		093C, 223T, 292T	W	W	—
deb12I	568	20	Infans I, m?	6,015±35 BP (KIA30400), 4,910±50 cal b.c.	298C	V	V	—
*deb03*	591	21	Adult, f	6,147±32 BP (KIA30401), 5,117±69 cal b.c.	147A, 172C, 223T, 248T, 320T, 355T	N1a	n.d.	
deb15	593	23	Infans I, f?		126C, 294T, 296T, 304C	T2	T	—
*deb05*	604/2	29	Infans II, f??		311C	HV	HV^b^	
deb22	604/3	30	Adult/mature, f		092C, 129A, 147A, 154C, 172C, 223T, 248T, 320T, 355T	N1a	N1	—
deb20	599	31	Adult, m	6,257±40 BP (KIA30403), 5,247±45 cal b.c.	311C	HV	HV	F*(xG,H,I,J,K)
deb21	600	32	Mature, f	6,151±27 BP (KIA30404), 5,122±65 cal b.c.	rCRS	H	H	
*deb01*	598	33	Infans II/Juvenile, f??		147A, 172C, 223T, 248T, 355T	N1a	N1	
*deb04*	596	34	Adult, m	6,141±33 BP (KIA30402), 5,112±73 cal b.c.	311C	HV	HV^b^	
deb26	606	37	Juvenile, m??		069T, 126C	J	J	—
deb32	640	38	Adult/mature f	6,142±34 BP (KIA30405), 5,112±73 cal b.c.	n.d.	n.d.	T	
deb30	592	40	Adult, f?		069T, 126C	J	J	—
deb29II	649	41	Adult, f?	6,068±31 BP (KIA30406), 4,982±38 cal b.c.	n.d.	n.d.	K	
deb34II	484	42	Adult/mature, m		093C, 223T, 292T	W	W	G2a3
deb33	483	43	Juvenile II, f??		126C, 147T, 293G, 294T, 296T, 297C, 304C	T2	T	—
*deb02*	644	44	Mature, f		224C, 311C	K	K	—
deb36	645	45	Mature, f		093C, 256T, 270T, 399G	U5a1a	U	
deb38	665	46	Adult/mature, m		093C, 224C, 311C	K	K	F*(xG,H,I,J,K)
deb35II	662	47	Adult, f?		126C, 189C, 294T, 296T	T	T	
deb37I	643	48	Adult/mature f		069T, 126C	J	J	
deb39	708	49	Adult/mature, f	6,148±33 BP (KIA30407), 5,117±69 cal b.c.	126C, 294T, 296T, 304C	T2	T	—

Italicized samples had been described previously [19].

a One versus two question marks after sex indicate two levels of insecurity in sexing.

b Previously analyzed diagnostic SNP sites at np 7028 AluI (hg H) and np 12308 HinfI (hg U) per restriction fragment length polymorphism.

BP, before present; f, female; m, male; n.d., not determined.

### Multiplex SNP Typing Assays

All of the mtDNA SNP typing results were concordant with the hg assignments based on HVS-I sequence information (Tables 1 and S1) and the known phylogenetic framework for the SNPs determined from modern populations [25]. The tight hierarchical structure of the latter provides a powerful internal control for contamination or erroneous results. Overall, both multiplex systems proved to be extremely time- and cost-efficient compared to the standard approach of numerous individual PCRs, and required 22–25 times less aDNA template while simultaneously reducing the chances of contamination dramatically. Also, both multiplex assays proved to be a powerful tool for analyzing highly degraded aDNA, and the GenoCoRe22 assay was able to unambiguously type four additional specimens that had failed to amplify more than 100 bp (Table 1) from two independent extractions. However, for reasons of overall data comparability, we could not include these specimens in downstream population genetic analyses, which required HVS-I sequence data. The only artifacts detected were occasional peaks in the electropherograms of the SNaPshot reactions outside the bin range of expected signals. These were probably due to primers and were mainly present in reactions from extracts with very little or no DNA template molecules; they were not observed with better preserved samples or modern controls.

In contrast, Y chromosome SNPs could be typed for only three out of the eight male individuals (37.5%; Table S2) identified through physical anthropological examination, reflecting the much lower copy number of nuclear loci [22]. After typing with the GenoY25 assay, individual deb34 was found to belong to hg G (M201), whereas individuals deb20 and deb38 both fall basally on the F branch (derived for M89 but ancestral for markers M201, M170, M304, and M9), i.e., they could be either F or H (Table 1). To further investigate the hg status beyond the standard GenoY25 assay, we amplified short fragments around SNP sites M285, P287, and S126 to further resolve deb34 into G1, G2*, and G2a3, and around SNP site M69 to distinguish between F and H [26]. deb34 proved to be ancestral for G1-M285 but derived for G2*-P287 and additional downstream SNP S126 (L30), placing it into G2a3. deb20 and deb38 were shown to be ancestral at M69 and hence basal F (M89), and remained in this position because we did not carry out further internal subtyping within the F clade.

The multiplexed single base extension (SBE) approach with its shortened flanking regions around targeted SNPs significantly increases the chance of successful Y-chromosomal amplifications, which have remained problematic for aDNA studies, as have nuclear loci in general, because of the much lower cellular copy number compared to mitochondrial loci. The multiplexed SBE approach promises to open the way to studying the paternal history of past populations, which is of paramount importance in determining how the social organization of prehistoric societies impacted the population dynamics of the past.

### Quantitative Real-Time PCR

Results of the qPCR revealed significantly (*p* = 0.012, Wilcoxon signed-ranks test) more mtDNA copies per microliter of each extract for the shorter fragment (141 bp) than for the longer (179 bp), with an average 3.7×10^4^–fold increase (detailed results are shown in Table S3). This finding is consistent with previous observations demonstrating a biased size distribution for authentic aDNA molecules [22],[27],[28] and suggests that any contaminating molecules, which would also result in higher copy numbers in the larger size class, did not significantly contribute to our amplifications.

### Population Genetic Analyses

To analyze the Neolithic mtDNA sequence diversity and characterize modern geographical affinities, we applied a range of population genetic analyses including shared haplotype analyses, principal component analyses (PCAs), multidimensional scaling (MDS), geographic mapping of genetic distances, and demographic modeling via Bayesian Serial Simcoal (BayeSSC) analyses (Table 2).

**Table 2:** Summary statistics, overview of population genetic analyses, and summary of haplogroup frequencies used for comparison with PCA vector loadings.

Category	Variable, Simulation, or Hg	Modern Datasets				Ancient Datasets^a^				
		Total Dataset	Pooled Geographic Sets of Equal Size (*n = *∼500)	Pooled European Dataset	Pooled Near East Populations	DEB22	LBK20	LBK42	LBK34	Hunter–Gatherers
**Summary statistics**	Populations	55	37	41	14	1	1	1	1	1
	Samples	23,394	18,039			22	20	42	34	20
**Population genetic analysis & simulations**	Shared haplotypes		X					X		
	PCA	X				X	X	X	X	X
	Relative hg frequencies			X	X	X	X	X	X	X
	MDS	X				X	X	X	X	
	Genetic distance maps	X				X		X		
	BayeSSC			X^b^	X^b^			X		X
	Haplotype diversity *h*					0.957	0.989	0.969	0.982	0.932
	Tajima's *D*					−0.91645	−0.90573	−0.91374	−0.88555	−1.05761
**Relative hg frequencies**	Asian hgs			1.62	2.09	0.00	0.00	0.00	0.00	0.00
	African hgs			0.65	6.43	0.00	0.00	0.00	0.00	0.00
	R0/preHV			0.37	3.26	0.00	0.00	0.00	0.00	0.00
	H			43.35	23.74	13.64	25.00	19.05	17.65	0.00
	HV			1.40	5.80	13.64	0.00	7.14	2.94	0.00
	J			8.49	10.59	13.64	5.00	9.52	5.88	4.76
	T			9.26	8.91	13.64	25.00	19.05	23.53	9.52
	I			2.23	1.97	0.00	0.00	0.00	0.00	0.00
	N1a			0.30	0.32	13.64	15.00	14.29	17.65	0.00
	K			5.39	6.67	13.64	15.00	14.29	14.71	4.76
	V			4.35	0.77	4.55	5.00	4.76	5.88	0.00
	W			2.03	2.25	9.09	5.00	7.14	5.88	0.00
	X			1.22	2.52	0.00	0.00	0.00	0.00	0.00
	U2			1.04	1.52	0.00	0.00	0.00	0.00	0.00
	U3			1.26	4.43	0.00	5.00	2.38	2.94	0.00
	U4			4.04	2.10	0.00	0.00	0.00	0.00	9.52
	U5a			5.46	2.53	4.55	0.00	2.38	2.94	23.80
	U5b			3.89	0.64	0.00	0.00	0.00	0.00	28.57
	Other rare hgs			3.67	13.45	0.00	0.00	0.00	0.00	19.05

X's indicate which datasets were used in the genetic analyses.

a For explanation of datasets, see Materials and Methods.

b For BayeSSC analyses, representative samples of the key areas were randomly drawn from the larger meta-population pool (Table S6).

### Shared Haplotype Analyses

**Figure 1:**
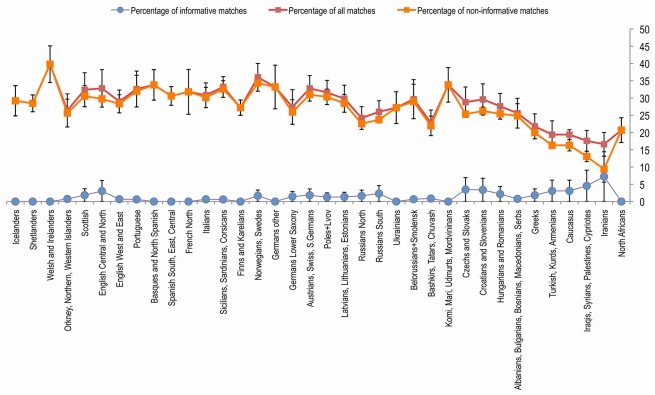
Percentages of shared haplotype matches per population. Populations are plotted on a northwest–southeast axis. Note that the percentage of non-informative matches (orange) is nearly identical to the percentage of all shared haplotypes (red) in most populations, whereas we observe elevated frequencies of informative matches (blue) in Southeast European and Near Eastern population pools, culminating in Iranians.

We prepared standardized modern population datasets of equal size (*n* = ∼500) from 36 geographical regions in Eurasia (*n* = 18,039; Table S4) to search for identical matches with each LBK haplotype. Out of 25 different haplotypes present in 42 LBK samples, 11 are found at high frequency in nearly all present-day populations under study, a further ten have limited geographic distribution, and the remaining four haplotypes are unique to Neolithic LBK populations (Table S4). The 11 widespread haplotypes are mainly basal (i.e., constituting a basal node within the corresponding hg) for Western Eurasian mitochondrial hgs H, HV, V, K, T, and W. While these haplotypes are relatively uninformative for identifying genetic affiliations to extant populations, this finding is consistent within an ancient population (5,500–4,900 cal b.c., i.e., prior to recent population expansions), in which basal haplotypes might be expected to be more frequent than derived haplotypes (e.g., end tips of branches within hgs). The next ten LBK haplotypes were unequally spread among present-day populations and for this reason potentially contain information about geographical affinities. We found nine modern-day population pools in which the percentage of these haplotypes is significantly higher than in other population pools (*p*>0.01, two-tailed *z* test; Figure 1; Table S4): (a) North and Central English, (b) Croatians and Slovenians, (c) Czechs and Slovaks, (d) Hungarians and Romanians, (e) Turkish, Kurds, and Armenians, (f) Iraqis, Syrians, Palestinians, and Cypriotes, (g) Caucasus (Ossetians and Georgians), (h) Southern Russians, and (i) Iranians. Three of these pools (b–d) originate near the proposed geographic center of the earliest LBK in Central Europe and presumably represent a genetic legacy from the Neolithic. However, the other matching population pools are from Near East regions (except [a] and [h]), which is consistent with this area representing the origin of the European Neolithic, an idea that is further supported by Iranians sharing the highest number of informative haplotypes with the LBK (7.2%; Table S4). The remaining pool (a) from North and Central England shares an elevated frequency of mtDNA T2 haplotypes with the LBK, but otherwise appears inconsistent with the proposed origin of the Neolithic in the Near East. It has been shown that certain alleles (here hgs) can accumulate in frequency while surfing on the wave of expansion, eventually resulting in higher frequencies relative to the proposed origin [29],[30]. Several of the other population pools also show a low but nonsignificant level of matches, which may relate to pre-Neolithic distributions or subsequent demographic movements (Figure 1).

Of the four unique mtDNA haplotypes, two were from an earlier study of the LBK (16286-16304 and 16319-16343; Table S5 and [19]). The haplotype 16286-16304 has many one- or two-step derivates in all parts of Europe and is therefore rather uninformative for inferring further geographical affinities. The only relatively close neighbor of haplotype 16319-16343 is found in Iraq (16129-16189-16319-16343), in agreement with the Near Eastern affinities of the informative LBK haplotypes. The other two unique LBK haplotypes belong to N1a, the characteristic LBK hg. The frequency of N1a was 13.6% for Derenburg samples (3/22) and 14.3% for all LBK samples published to date (6/42). Notably, N1a has not yet been observed in the neighboring hunter–gatherer populations of Central Europe before, during, or after the Early Neolithic [20] nor in the early Neolithic Cardial Ware Culture from Spain [18].

The Y chromosome hgs obtained from the three Derenburg early Neolithic individuals are generally concordant with the mtDNA data (Table 1). Interestingly, we do not find the most common Y chromosome hgs in modern Europe (e.g., R1b, R1a, I, and E1b1), which parallels the low frequency of the very common modern European mtDNA hg H (now at 20%–50% across Western Eurasia) in the Neolithic samples. Also, while both Neolithic Y chromosome hgs G2a3 and F* are rather rare in modern-day Europe, they have slightly higher frequencies in populations of the Near East, and the highest frequency of hg G2a is seen in the Caucasus today [15]. The few published ancient Y chromosome results from Central Europe come from late Neolithic sites and were exclusively hg R1a [31]. While speculative, we suggest this supports the idea that R1a may have spread with late Neolithic cultures from the east [31].

### Principal Component Analysis and Multidimensional Scaling

**Figure 2:**
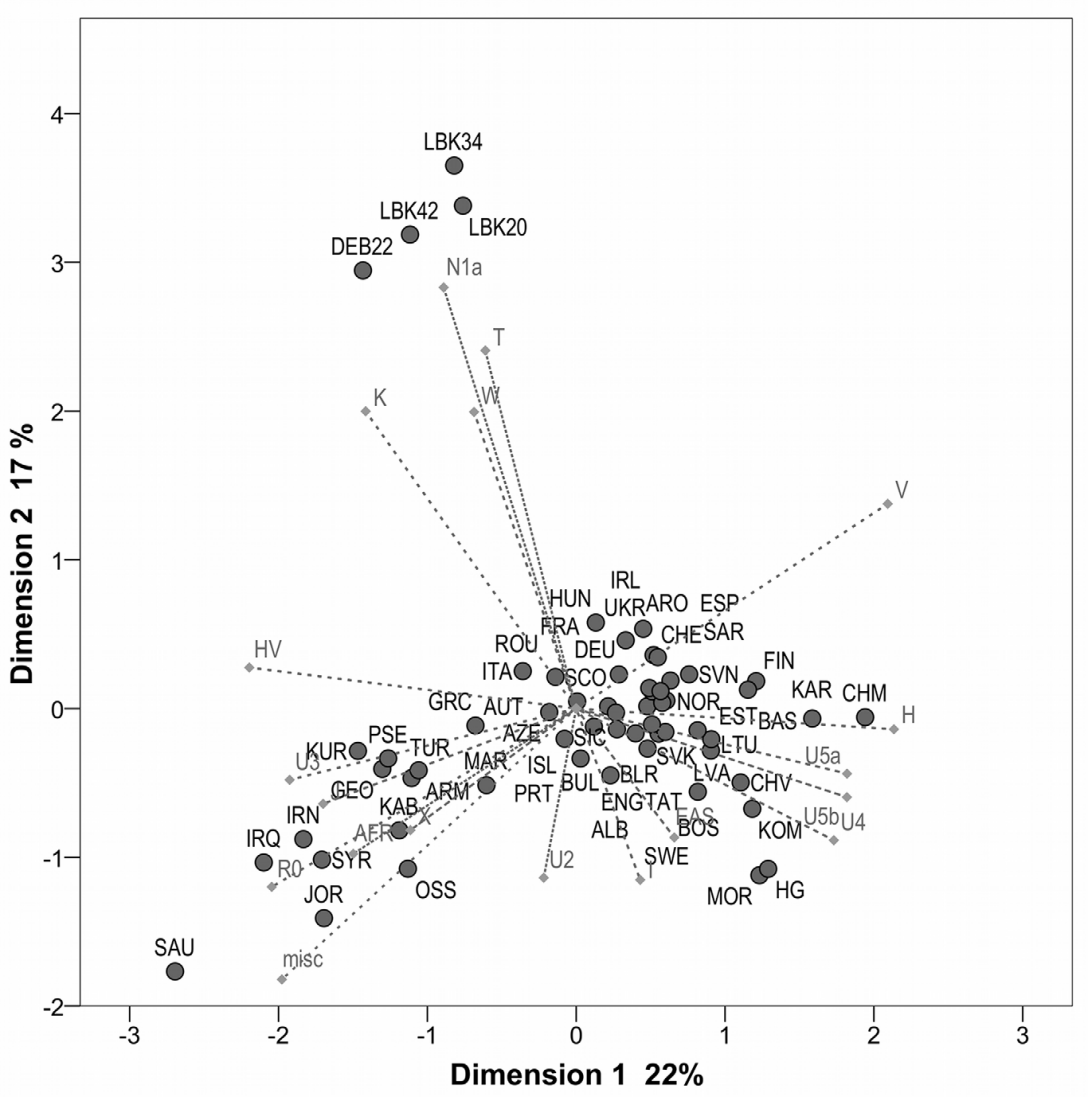
PCA plot based on mtDNA haplogroup frequencies. The two dimensions display 39% of the total variance. The contribution of each hg is superimposed as grey component loading vectors. Notably, the Derenburg dataset (DEB22) groups well with its meta-population (LBK20), supporting the unique status and characteristic composition of the LBK sample. Populations are abbreviated as follows (Table S6): ALB, Albanians; ARM, Armenians; ARO, Aromuns; AUT, Austrians; AZE, Azeris; BAS, Basques; BLR, Byelorussians; BOS, Bosnians; BUL, Bulgarians; CHE, Swiss; CHM, Mari; CHV, Chuvash; CRO, Croats; CZE, Czechs; DEB22, Derenburg; DEU, Germans; ENG, English; ESP, Spanish; EST, Estonians; FIN, Finns; FRA, French; GEO, Georgians; GRC, Greeks; HG, European Mesolithic hunter–gatherers.; HUN, Hungarians; IRL, Irish; IRN, Iranians; IRQ, Iraqis; ISL, Icelanders; ITA, Italians; JOR, Jordanians; KAB, Kabardinians; KAR, Karelians; KOM, Komis (Permyaks and Zyrian); KUR, Kurds; LBK20, LBK without Derenburg; LBK34, all LBK samples excluding potential relatives; LBK42, all LBK; LTU, Lithuanians; LVA, Latvians; MAR, Moroccans; MOR, Mordvinians; NOG, Nogais; NOR, Norwegians; OSS, Ossetians; POL, Poles; PRT, Portuguese; PSE, Palestinians; ROU, Romanians; RUS, Russians; SAR, Sardinians; SAU, Saudi Arabians; SCO, Scots; SIC, Sicilians; SVK, Slovaks; SVN, Slovenians; SWE, Swedes; SYR, Syrians; TAT, Tatars; TUR, Turkish; UKR, Ukrainians.

Four Neolithic datasets were constructed (Table 2) and compared with 55 present-day European and Near Eastern populations and one Mesolithic hunter–gatherer population [20] in a PCA (Figure 2). The PCA accounted for 39% of the total genetic variation, with the first principal component (PC) separating Near Eastern populations from Europeans (24.9%), and with LBK populations falling closer to Near Eastern ones. However, the second PC (17.4%) clearly distinguished the four Neolithic datasets from both Near East and European populations. An MDS plot (Figure S1) showed similar results, with the Near Eastern affinities of the LBK populations even more apparent.

To better understand which particular hgs made the Neolithic populations appear either Near Eastern or (West) European, we compared average hg frequencies of the total LBK (LBK42) and Derenburg (DEB22) datasets to two geographically pooled meta-population sets from Europe and the Near East (Tables 2 and S6; 41 and 14 populations, respectively). PC correlates and component loadings (Figure 2) showed a pattern similar to average hg frequencies (Table 2) in both large meta-population sets, with the LBK dataset grouping with Europeans because of a lack of mitochondrial African hgs (L and M1) and preHV, and elevated frequencies of hg V. In contrast, low frequencies of hg H and higher frequencies for HV, J, and U3 promoted Near Eastern resemblances. Removal of individuals with shared haplotypes within the Derenburg dataset (yielding dataset LBK34) did not noticeably decrease the elevated frequencies of J and especially HV in the Neolithic data.

Most importantly, PC correlates of the second component showed that elevated or high frequencies of hgs T, N1a, K, and W were unique to LBK populations, making them appear different from both Europe and Near East. The considerable within-hg diversity of all four of these hgs (especially T and N1a; Table 1) suggests that this observation is unlikely to be an artifact of random genetic drift leading to elevated frequencies in small, isolated populations.

The pooled European and Near Eastern meta-populations are necessarily overgeneralizations, and there are likely to be subsets of Near Eastern populations that are more similar to the Neolithic population. Interestingly, both the PCA and the MDS plots identified Georgians, Ossetians, and Armenians as candidate populations (Figures 2 and S1).

### Mapping Genetic Distances

**Figure 3:**
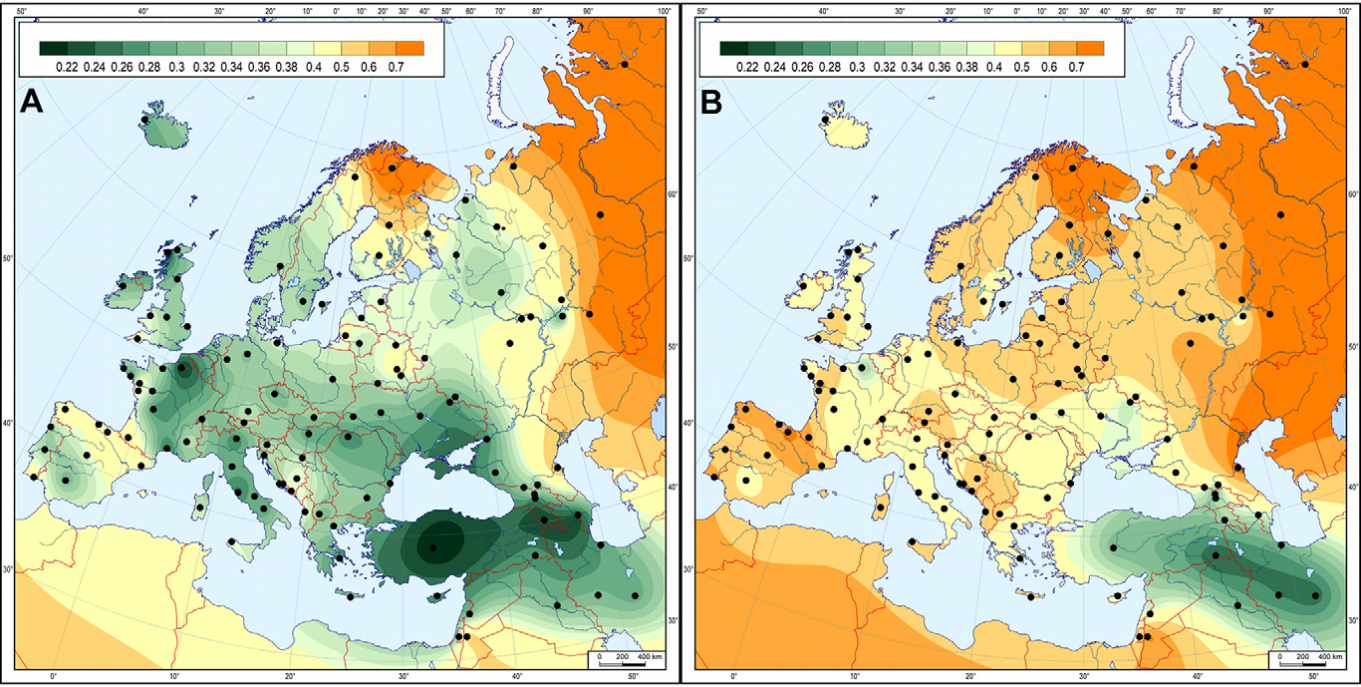
Genetic matrilineal distances between 55 modern Western Eurasian populations (Table S6) and Neolithic LBK samples. Mapped genetic distances are illustrated between 55 modern Western Eurasian populations and the total of 42 Neolithic LBK samples (A) or the single graveyard of Derenburg (B). Black dots denote the location of modern-day populations used in the analysis. The coloring indicates the degree of similarity of the modern local population(s) with the Neolithic sample set: short distances (greatest similarity) are marked by dark green and long distances (greatest dissimilarity) by orange, with fainter colors in between the extremes. Note that green intervals are scaled by genetic distance values of 0.02, with increasingly larger intervals towards the “orange” end of the scale.

We generated genetic distance maps to visualize the similarity/distance of the LBK and Derenburg populations (datasets LBK42 and DEB22) to all modern populations in the large Western Eurasian dataset (Figure 3). In agreement with the PCA and MDS analyses, populations from the area bounding modern-day Turkey, Armenia, Iraq, and Iran demonstrated a clear genetic similarity with the LBK population (Figure 3A). This relationship was even stronger in a second map generated with just the Neolithic Derenburg individuals (Figure 3B). Interestingly, the map of the combined LBK data also suggested a possible geographic route for the dispersal of Neolithic lineages into Central Europe: genetic distances gradually increase from eastern Anatolia westward across the Balkans, and then northwards into Central Europe. The area with lower genetic distances follows the course of the rivers Danube and Dniester, and this natural corridor has been widely accepted as the most likely inland route towards the Carpathian basin as well as the fertile Loess plains further northwest [23],[32],[33].

### Bayesian Serial Simcoal Analysis

While an apparent affinity of Neolithic farmers to modern-day Near East populations is revealed by the shared haplotype analyses, PCA, MDS, and genetic distance maps, the population-specific pairwise *F*_ST_ values among ancient populations (hunter–gatherers and LBK) and the modern population pools (Central Europe and Near East) tested were all significant (*p*>0.05; Table 3), suggesting a degree of genetic discontinuity between ancient and modern-day populations. The early farmers were closer to the modern Near Eastern pool (*F*_ST_ = 0.03019) than hunter–gatherers were (*F*_ST_ = 0.04192), while both ancient populations showed similar differences to modern Central Europe, with the hunter–gatherers slightly closer (*F*_ST_ = 0.03445) than the early farmers (*F*_ST_ = 0.03958). The most striking difference was seen between Mesolithic hunter–gatherers and the LBK population itself (*F*_ST_ = 0.09298), as previously shown [20]. We used BayeSSC analyses to test whether the observed *F*_ST_ values can be explained by the effects of drift or migration under different demographic scenarios (Figure S2). This encompassed comparing *F*_ST_ values derived from coalescent simulations under a series of demographic models with the observed *F*_ST_ values in order to test which model was the most likely, given the data. By using an approximate Bayesian computation (ABC) framework we were able to explore priors for initial starting deme sizes and dependent growth rates to maximize the credibility of the final results. The Akaike information criterion (AIC) was used to evaluate a goodness-of-fit value of the range of models in the light of the observed *F*_ST_ values. In addition, a relative likelihood estimate for each of the six models given the data was calculated via Akaike weights (ω). The highest AIC values, and therefore the poorest fit, were obtained for models representing population continuity in one large Eurasian meta-population through time (Models H_0_a and H_0_b; Table 4). Of note, the goodness of fit was better with a more recent population expansion (modeled at the onset of the Neolithic in Central Europe) and hence higher exponential growth rate (H_0_a). The model of cultural transmission (H_1_), in which a Central European deme including Neolithic farmers and hunter–gatherers coalesced with a Near Eastern deme in the Early Upper Paleolithic (1,500 generations, or ∼37,500 y ago), resulted in intermediate goodness-of-fit values (H_1_a and H_1_b; Table 4; Figure S2). The best goodness-of-fit values were retrieved for models of demic diffusion (model H_2_; Table 4) with differing proportions of migrants (25%, 50%, and 75% were tested) from the Near Eastern deme into the Central European deme around the time of the LBK (290 generations, ∼7,250 y ago; Table 4). Notably, the models testing 50% and 75% migrants returned the highest relative likelihood values (42% and 52%, respectively), and therefore warrant further investigation. However, while the demic diffusion model H_2_ produced values that approximated the observed *F*_ST_ between Neolithic farmers and the Near Eastern population pool, none of the models could account for the high *F*_ST_ between hunter–gatherers and early farmers or early farmers and modern-day Central Europeans.

**Table 3:** Pairwise *F*_ST_ values between ancient and modern-day population pools as used for goodness-of-fit estimates in BayeSSC analyses.

	Hunter–Gatherers	Near East	LBK	Central Europe
**Hunter–Gatherers**	0	—	—	—
**Near East**	0.04192	0	—	—
**LBK**	0.09298	0.03019	0	—
**Central Europe**	0.03445	0.00939	0.03958	0

**Table 4:** Details of the demographic models analyzed with BayeSSC and AIC goodness-of-fit estimates, and resulting model probabilities via Akaike weights.

Model	H_0_a	H_0_b	H_1_	H_2_	H_2_	H_2_
Prior *N*_e_, time 0 ,deme 0	U^a^:100000,30000000	U:100000,30000000	U:100000,12000000	U:100000,12000000	U:100000,12000000	U:100000,12000000
Prior *N*_e_, time 0, deme 1			U:100000,12000000	U:100000,12000000	U:100000,12000000	U:100000,12000000
Percent migrants from deme 0 to deme 1				25%	50%	75%
AIC	97.78	120.37	89.19	82.56	78.52	78.07
Akaike weight ω	2.76164e^−5^	3.42478e^−10^	0.002018032	0.055596369	0.418527622	0.52383036

Of note, the smaller the AIC value, the better the fit of the model. While no threshold value can be assigned to AIC values at which any model can be rejected, the Akaike weights estimate a model probability given the six models tested.

a U, uniform distribution of given range.

*N*_e_, effective population size.

The models we tested represent major oversimplifications and it should be noted that modeling human demographic history is notoriously difficult, especially given the complex history of Europe and the Near East over this time scale. The fact that no model explained the observed *F*_ST_ between ancient and modern-day populations particularly well suggests that the correct scenario has not yet been identified, and that there is also an obvious need for sampling of material from younger epochs. Additionally, sampling bias remains an issue in aDNA studies, and this is particularly true for the chronologically and geographically diverse hunter–gatherer dataset. In the light of the models tested (see also [19],[20]), we would suggest that the basis of modern European mtDNA diversity was formed from the postglacial re-peopling of Europe (represented here by the Mesolithic hunter–gatherers) and the genetic input from the Near East during the Neolithic, but that demographic processes after the early Neolithic have contributed substantially to shaping Europe's contemporary genetic make up.

### Synthesis of Population Genetic Analyses

The aDNA data from a range of Mesolithic hunter–gatherer samples from regions neighboring the LBK area have been shown to be surprisingly homogenous across space and time, with an mtDNA composition almost exclusively of hg U (∼80%), particularly hg U4 and U5, which is clearly different from the LBK dataset as well as the modern European diversity (Table 2) [20]. The observation that hgs U4 and U5 are virtually absent in the LBK population (1/42 samples) is striking (Table 2). Given this clear difference in the mtDNA hg composition, it is not surprising that the pairwise *F*_ST_ between hunter–gatherers and the LBK population is the highest observed (0.09298) when we compared ancient populations with representative population pools from Central Europe and the Near East (Table 3; see also [20]). If the Mesolithic data are a genuine proxy for populations in Central Europe at the onset of the LBK, it implies that the Mesolithic and LBK groups had clearly different origins, with the former potentially representing the pre-Neolithic indigenous groups who survived the Last Glacial Maximum in southern European refugia. In contrast, our population genetic analyses confirm that the LBK shares an affinity with modern-day Near East and Anatolia populations. Furthermore, the large number of basal lineages within the LBK, a reasonably high hg and haplotype diversity generated through one- or two-step derivative lineages, and the negative Tajima's *D* values (Tables 1 and 2) indicate a recent expansion. These combined data are compatible with a model of Central Europe in the early Neolithic of indigenous populations plus significant inputs from expanding populations in the Near East [4],[12],[34]. Overall, the mtDNA hg composition of the LBK would suggest that the input of Neolithic farming cultures (LBK) to modern European genetic variation was much higher than that of Mesolithic populations, although it is important to note that the unique characteristics of the LBK sample imply that further significant genetic changes took place in Europe after the early Neolithic.

aDNA data offers a powerful new means to test evolutionary models and assumptions. The European lineage with the oldest coalescent age, U5, has indeed been found to prevail in the indigenous hunter–gatherers [12],[35]. However, mtDNA hgs J2a1a and T1, which because of their younger coalescence ages have been suggested to be Neolithic immigrant lineages [8],[12], are so far absent from the samples of early farmers in Central Europe. Similarly, older coalescence ages were used to support hgs K, T2, H, and V as “postglacial/Mesolithic lineages,” and yet these have been revealed to be common only in Neolithic samples. The recent use of whole mitochondrial genomes and the refinement of mutation rate estimates have resulted in a general reduction in coalescence ages [8], which would lead to an improved fit with the aDNA data. However we advise caution in directly relating coalescence ages of specific hgs to evolutionary or prehistoric demographic events [36]. Significant temporal offsets can be caused by either observational bias (the delay between the actual split of a lineage and the eventual fixation and dissemination of this lineage) or calculation bias (incorrect coalescent age estimation). aDNA has considerable value not only for directly analyzing the presence or absence of lineages at points in the past but also for refining mutation rate estimates by providing internal calibration points [37].

Archaeological and anthropological research has produced a variety of models for the dispersal of the Neolithic agricultural system (“process of Neolithization”) into and throughout Europe (e.g., [1],[2],[38]). Our findings are consistent with models that argue that the cultural connection of the LBK to its proposed origin in modern-day Hungary, and reaching beyond the Carpathian Basin [23],[32],[38],[39], should also be reflected in a genetic relationship (e.g., shared haplotype analyses; Table S4). Therefore at a large scale, a *demic diffusion* model of genetic input from the Near East into Central Europe is the best match for our observations. It is notable that recent anthropological research has come to similar conclusions [40],[41]. On a regional scale, “leap-frog” or “individual pioneer” colonization models, where early farmers initially target the economically favorable Loess plains in Central Europe [33],[42], would explain both the relative speed of the LBK expansion and the clear genetic Near Eastern connections still seen in these pioneer settlements, although the resolving power of the genetic data is currently unable to test the subtleties of these models.

In conclusion, the new LBK dataset provides the most detailed and direct genetic portrait of the Neolithic transition in Central Europe; analysis of this dataset reveals a clear demonstration of Near Eastern and Anatolian affinities and argues for a much higher genetic input from these regions, while also identifying characteristic differences from all extant (meta-)populations studied. Ancient genetic data from adjacent geographic regions and time periods, and especially from the Near East and Anatolia, will be needed to more accurately describe the changing genetic landscape during and after the Neolithic, and the new multiplexed SBE assays offer a powerful means to access this information.

## Materials and Methods

### Archaeological Background

The archaeological site Derenburg Meerenstieg II (Harzkreis, Saxony-Anhalt, Germany) was excavated during three campaigns in 1997–1999 comprising an area of 3 ha. The archaeological context at this site shows a record of settlement activity ranging from the Early Neolithic (LBK) and Middle Neolithic (Rössen and Ammensleben cultures) to Bronze and Iron Age [43]. However, the main features of Derenburg are the LBK graveyard and its associated partial settlement approximately 70 m southwest. The archaeological data revealed that the larger part of the settlement has not yet been excavated and lies outside the area covered during these campaigns. In contrast, the graveyard was recorded in its entire dimension (25×30 m) and encompassed a total of 41 graves. Two separate graves were found outside the graveyard (50 m WSW and 95 m SSE). Erosion and modern agricultural ploughing might have led to a loss of some graves at the plateau area. Here, the graves were shallow and in average state of preservation, whereas the graves embedded in deeper Loess layers showed an excellent state of preservation. In total, 32 single grave burials were found; there were also one double burial, one triple burial, two burials in settlement pits, two or three times additional singular bones in a grave, three burials with a secondary inhumation, and one empty grave. The majority of individuals (75%) at Derenburg were buried in East–West orientation in a varying flexed position. The duration of usage of the graveyard spans over the entire time frame of the LBK and is reflected by the typology of the ceramics and associated grave goods ranging from older LBK pottery (Flomborn style) to youngest LBK pottery. Absolute radiocarbon dates confirm the usage over three centuries (5,200–4,900 cal b.c.; see also Table 1 and [44]).

### Ancient DNA Work

From an initial 43 graves in the Derenburg graveyard, 31 indicated morphological preservation suitable for sampling and aDNA analyses. Five individuals had already been sampled in 2003 for our previous study and showed excellent preservation of aDNA, a negligible level of contamination, and an unusual mtDNA hg distribution, thereby justifying further investigation [19]. Hence, 26 additional individuals were processed in this study (Table 1). We amplified, cloned, and sequenced mitochondrial HVS-I (nucleotide positions [np] 15997–16409; nucleotide position according to [45]) as described previously [19]. mtDNA hg assignments were further supported by typing with a newly developed multiplex of 22 mtDNA coding region SNPs (GenoCoRe22). In addition, we typed 25 Y chromosome SNPs using a second novel multiplex assay (GenoY25). Final refinement of Y chromosome hg assignments was performed via singleplex PCRs. Lastly, the amount of starting DNA template molecules was monitored using qPCR on seven random samples (Table S3). aDNA work was performed in specialized aDNA facilities at the Johannes Gutenberg University of Mainz and the Australian Centre for Ancient DNA (ACAD) at the University of Adelaide according to appropriate criteria. All DNA extractions as well as amplification, cloning, and sequencing of the mitochondrial control region HVS-I were carried out in the Johannes Gutenberg University of Mainz facilities. Additional singleplex, all multiplex, and quantitative real-time amplifications, SNP typing, and direct sequencing of Y chromosome SNPs were carried at the ACAD as described below.

### SNP Selection and Multiplex Design

The technique of SNP typing via SBE reactions (also known as minisequencing) has proven a reliable and robust method for high throughput analyses of polymorphisms, e.g., human mitochondrial variation [46], human X- and Y-chromosomal SNPs [47],[48], and human autosomal SNPs [49]. However, few SBE studies have addressed the special need for very short amplicon sizes to allow amplification from highly degraded DNA, as even forensic protocols have generally targeted relatively long amplicon sizes [50]–[54]. Our first multiplex (GenoCoRe22) was designed to type a panel of 22 mitochondrial coding region SNPs that are routinely typed within the Genographic Project [25], to allow for future maximum comparability with modern population data. A second multiplex (GenoY25) targeted a basal, but global, coverage of 25 commonly typed Y chromosome SNPs, for maximum comparability of paternal lineages. The aim of the SNP assay design was to produce highly efficient and sensitive protocols, capable of working on highly degraded DNA, that also allow modern human DNA contamination to be detected at very low levels and monitored [51]. The GenoCoRe22 SNP panel was chosen to cover the basal branches of mitochondrial hgs across modern human mtDNA diversity [25]. The chosen SNP sites were identical to the initial set (Figure 4 in [25]) except for hg W (SNP at np 8994 instead of np 1243) and hg R9 (SNP at np 13928 instead of np 3970), as a compromise arising from primer design within a multiplex assay. Selection of GenoY25 SNP panel for incorporation into the multiplex assay was performed using the highly resolved Y Chromosome Consortium tree and an extensive literature search for corresponding SNP allele frequencies in European populations [13],[26],[55].

### Multiplex PCR Assays GenoCoRe22 and GenoY25

Multiplex assays were set up, established, and performed at the ACAD facilities. Multiplex PCR using Amplitaq Gold (Applied Biosystems) was conducted in 25-µl volumes using 1× Buffer Gold, 6 mM (GenoCoRe22) or 8 mM (GenoY25) MgCl_2_, 0.5 mM dNTPs (Invitrogen), ≤0.2 µM of each primer, 1 mg/ml RSA (Sigma), 2 U of Amplitaq Gold Polymerase, and 2 µl of DNA extract. Thermocycling conditions consisted of an initial enzyme activation at 95°C for 6 min, followed by 40–45 cycles of denaturation at 95°C for 30 s, annealing at 60°C (GenoCoRe22) or 59°C (GenoY25) for 30 s, and elongation at 65°C for 30 s, with a single final extension time at 65°C for 10 min. Each PCR included extraction blanks as well as a minimum of two PCR negatives at a ratio of 5∶1. PCRs were visually checked by electrophoresis on 3.5% agarose TBE gels. PCR products were purified by mixing 5 µl of PCR product with 1 U of SAP and 0.8 U of ExoI and incubating at 37°C for 40 min, followed by heat inactivation at 80°C for 10 min. Because of the sensitivity of the multiplex PCR (using fragment lengths of only 60–85 bp), and to be able to monitor potential human background contamination, usually all controls were included in downstream fragment analysis. Multiplex primer sequences and concentration are given in Table S7.

### SNaPshot Typing

SBE reactions were carried out on the GenoCoRe22 and GenoY25 SNP multiplex assay using the ABI Prism SNaPshot multiplex reaction kit (Applied Biosystems) following the manufacturer's instructions, except that 10% 3 M ammonium sulfate was added to the extension primer mix to minimize artifacts [56]. SBE primers and concentrations are given in Table S7. Cycling conditions consisted of 35 cycles of denaturation at 96°C for 10 s, annealing at 55°C for 5 s, and extension at 60°C for 30 s. SBE reactions were purified using 1 U of SAP, incubating at 37°C for 40 min, followed by heat inactivation at 80°C for 10 min. Prior to capillary electrophoresis, 2 µl of purified SNaPshot product was added to a mix of 11.5 µl of Hi-Di Formamide (Applied Biosystems) and 0.5 µl of Gene-Scan-120 LIZ size standard (Applied Biosystems). Samples were run on an ABI PRISM 3130xl Genetic Analyzer (Applied Biosystems) after a denaturation carried out according to the manufacturer's instructions using POP-6 (Applied Biosystems). Evaluation and analyses of SNaPshot typing profiles were performed using custom settings within the GeneMapper version 3.2 Software (Applied Biosystems).

### Y Chromosome SNP Singleplex PCRs and Sequencing

Additional Y chromosome SNPs (M285, P287 S126, and M69) were tested to determine specific downstream subclades based on the initial multiplex results in order to gain further resolution. We chose appropriate SNP loci by following general criteria, trying to keep the PCR amplicon size smaller than 90 bp in size and flanking DNA sequences free from interfering polymorphisms, such as nucleotide substitutions in potential primer binding sites. We selected PCR amplification primers that have a theoretical melting temperature of around 60°C in neutral buffered solutions (pH 7–8), with monovalent cation (Na^+^) concentrations at 50 mM and divalent cation (Mg^++^) concentrations at 8 mM. All primer candidates were analyzed for primer–dimer formation, hairpin structures, and complementarities to other primers in the multiplex using Primer 3 (http://primer3.sourceforge.net/). Primer characteristics were chosen to ensure equal PCR amplification efficiency for all DNA fragments, as previously described [50]. The primers were HPLC-purified and checked for homogeneity by MALDI-TOF (Thermo). Table S7 shows the sequences and the concentrations of the amplification primers in the final multiplex PCR.

Additional Y chromosome SNP singleplex PCRs were carried out in the ACAD facilities. Standard PCRs using Amplitaq Gold (Applied Biosystems) were conducted in 25-µl volumes using 1× Buffer Gold, 2.5 mM MgCl_2_, 0.25 mM of each dNTP (Fermentas), 400 µM of each primer (Table S7), 1 mg/ml RSA (Sigma-Aldrich), 2 U of Amplitaq Gold Polymerase, and 2 µl of DNA extract. Thermocycling conditions consisted of an initial enzyme activation at 95°C for 6 min, followed by 50 cycles of denaturation at 94°C for 30 s, annealing at 59°C for 30 s, and elongation at 72°C for 30 s, with a single final extension time at 60°C for 10 min. Each PCR reaction included extraction blanks as well as a minimum of two PCR negatives. PCR products were visualized and purified as described above and were directly sequenced in both directions using the Big Dye Terminator 3.1 Kit (Applied Biosystems) as per manufacturer's instructions. Sequencing products were purified using Cleanseq magnetic beads (Agencourt, Beckman Coulter) according to the manufacturer's protocol. Sequencing products were separated on a 3130xl Genetic Analyzer (Applied Biosystems), and the resulting sequences were edited and aligned relative to the SNP reference sequence (GenBank SNP accession numbers: M285, rs13447378; P287, rs4116820; S126 [also known as L30], rs34134567; and M69, rs2032673) using the software Sequencher 4.7 (Genecodes).

### Quantitative Real-Time PCR

qPCR was used to determine the amount of DNA in the samples prior to amplification and to assess the authenticity based on the assumption that there is an inverse relationship between DNA quantity and fragment length for degraded aDNA [57],[58]. Two different length fragments were amplified from the HVS-I: 141 bp (L16117/H16218) and 179 bp (L16209/H16348) [19],[59]. All qPCR reactions were carried out in a 10-µl reaction volume containing 1× Express SYBR Green ER Supermix Universal (Invitrogen), rabbit serum albumin (10 mg/ml), forward and reverse primers (10 µM), and 1 µl of DNA extract. Thermocycling conditions consisted of an initial enzyme activation at 95°C for 5 min, followed by 50 cycles of 94°C for 10 s, 58°C for 20 s, and 72°C for 15 s. The primer specificity was assessed using a post-PCR melt curve to visualize the dissociation kinetics. The primers were validated using modern DNA, and a single peak was observed for both fragments, indicating specific binding. The dissociation temperature (*T*_M_) was 80–80.3°C for the 141-bp fragment and 81.7–82.3°C for the 179-bp fragment. Both primer pairs showed an absence of primer dimers, indicated by the lack of a smaller peak on the melt curve (≈60°C) and a single band on a 2% agarose gel. The starting quantity of DNA in the ancient samples was determined by comparison to a standard curve of a known amount of DNA_._ The standard curves for the two fragments were created from modern human DNA. The DNA was extracted from a buccal cheek swab of a single individual using DNeasy Blood and Tissue Kit (Qiagen). mtDNA was amplified for the two fragments (141 bp and 179 bp) using 1× Hotmaster Buffer (Eppendorf), 0.5 U of Hotmaster Taq (5Prime), forward and reverse primers (10 µM), distilled water, and 2 µl of DNA extract. Thermocycling conditions consisted of an initial enzyme activation at 94°C for 2 min, followed by 30 cycles of 94°C for 20 s, 60°C for 10 s, and 65°C for 1 min. The PCR products were purified using Agencourt Ampure (Beckman Coulter) according to manufacturer's instructions. The DNA concentration for the 141-bp and 179-bp amplicons was measured twice at 1∶1 and 1∶10 dilutions with a Nanovue (GE Healthcare). Ten-fold serial dilutions, from 1×10^6^ to 10 copies/µl, of the purified fragments were used to make the standards. These were run with the qPCR conditions described above. For each standard, each 10-fold dilution was run in triplicate and the qPCR was repeated on a separate day. All the standards met the following criteria: (1) there was a linear regression relationship between DNA quantity and cycle threshold (fluorescence above background), *R* ^2^>0.95, and (2) the reaction was efficient (i.e., a doubling of product per cycle in the exponential phase), between 90% and 110%. Ancient qPCRs were run in triplicate with extraction and PCR blanks, and PCR standards (positive control) run in duplicate. Amplifications were performed on Rotor-Gene 6000 and analyses on Rotor-Gene 6000 Series Software 1.7 (Corbett). The difference in mtDNA quantity between fragment lengths (141 and 179 bp) was assessed using a nonparametric version of a Student's *t* test, a Wilcoxon signed-ranks test. This test was selected because the data were not appropriate for a parametric test, displaying a mixture of normal (179 bp, *p* = 0.425) and non-normal (141 bp, *p* = 0.012) distributions, as determined from a Shapiro-Wilk *W* test, which is appropriate for testing the normality of groups with small sample sizes.

### Authentication Criteria

In line with previous publications on aDNA and especially with criteria for working with human aDNA, it can be stated that a 100% authentication of ancient samples is virtually impossible [22],[57],[60]. However, we took all possible precautions to prevent modern contaminations, and we regard the results as authentically derived from endogenous DNA based on the following chain of evidence. (1) All samples were collected under DNA-free conditions after excavation. Samples were not washed, treated, or examined before taking DNA samples. (2) All preparation and analytical steps prior to DNA amplification were conducted in a clean room area solely dedicated to aDNA work located in a physically separated building without any modern DNA work (pre-PCR area). Amplification, cloning, and sequencing were carried out in the post-PCR lab. (3) All steps were monitored by non-template controls and by using bovid samples in parallel. (4) All individuals were sampled twice from anatomically independent regions and treated independently. At least eight independent PCR reactions were carried out (four overlapping fragments × two extractions) per individual. In case of successful amplification of all eight fragments, these were cloned and an average of eight clones per amplicons was sequenced to detect heterogeneous sequences due to DNA degradation or contamination. All replicable polymorphic sites were consistent with existing mtDNA haplotypes, ruling out post mortem DNA damage as a potential source for erroneous sequences. (5) The new multiplexes not only clearly confirm hg assignment but also provide an ideal monitoring system for ancient human DNA samples, as they directly target SNPs defining all potential contaminating lineages. (6) qPCR was carried out on a selection of samples to ensure appropriate levels of DNA quantity and to assess DNA quality. (7) Samples were collected and processed by W. H. exclusively (mtDNA hg H1, np 15997–16409: 16189C 16311C, and Y chromosome hg E1b1b1a-M78) after excavation; no other staff were involved in any of the pre-PCR steps. Eventually, all listed criteria indicating authenticity or at least the plausibility of having retrieved endogenous DNA were evaluated, together with the sample's post-excavation history [60].

### Populations under Study

Four partly overlapping Neolithic datasets were analyzed: the 22 Derenburg individuals (DEB22); 20 individuals from other LBK populations previously published (LBK20; Table S5 and [19]); the combined LBK dataset (LBK42); and the combined LBK dataset excluding eight individuals of possible kinship (LBK34, see below) to avoid overestimation of haplotype frequencies. These four Neolithic sets were analyzed against extant population data from the MURKA mitochondrial DNA database and integrated software, currently containing 97,523 HVS-I records from published sources, and maintained by coauthors V. Z., E. B., and O. B. of the Russian Academy of Medical Sciences. Analyses were restricted to 390 populations from Europe and the Near East (35,757 mtDNAs). For detailed analysis of shared haplotypes, we included only sequences spanning from np 16069 to np 16365 (34,258 samples, haplotype dataset). aDNA sequences were trimmed to the same length. For frequency-based analyses (PCA, MDS, and genetic distance maps), we omitted mtDNAs whose hg affiliations were ambiguous (absence of information on coding region SNPs), resulting in our final hg frequency dataset of 23,394 individuals from 228 population studies, which subsequently were pooled into 55 populations based on ethnicity, language, and/or geographical criteria as described in the original publications (see Table S6).

### Addressing Potential Kinship within the Derenburg Graveyard

The mtDNA and Y chromosome hg results were overlaid onto the map of the graveyard to elucidate the spatial relationships within the graveyard (Figure S3). Four haplotypes were shared by two individuals each, and two haplotypes by three individuals each, while the remaining eight individuals (36.4%) showed unique haplotypes within the Derenburg graveyard. A number of shared haplotypes is not surprising in a medium sized, closed LBK graveyard where the influence of genetic drift and a certain level of biological kinship are likely. However, little positional structuring according to maternal lineages was observed. A clustering of mtDNA haplotypes H-rCRS (deb9 and deb21) and HV (deb4, deb20, and deb5) in the northwest corner of the cemetery is notable, whereas other shared haplotype “twins” or “trios” with a potential maternal relationship are spread across larger distances. However, it must be stated that there are many other factors influencing the layout of interments in a graveyard that cannot be unraveled by aDNA analyses. LBK burials commonly show a great variety of mortuary patterns or rites at the same site (e.g., burials within the settlement and burials in pits/middens), and it is therefore not clear whether individuals in the cemetery represent the norm or the exception, and how much of the initial genetic variation of the population is missing [44]. In any case, to avoid overestimation of haplotype frequencies in the LBK dataset, the eight duplicate haplotypes were excluded, and a reduced dataset (LBK34) was used in population genetic analyses alongside the complete set to account for a potential kinship effect.

### Haplotype Diversity and Tajima's *D*

Haplotype diversity (*h*) and Tajima's *D* were calculated using DnaSP version 5 [61].

### Shared Haplotype Analysis

In order to calculate the percentage of shared haplotypes between the LBK sample and modern-day populations, we chose modern populations of equal or larger sample sizes, resulting in 36 out of 55 pooled populations with sample size *n* = 500 or above. Pooling was based on geographic proximity and linguistic similarity. For population studies with *n*>500, 500 samples were selected randomly. After pooling and random selection the dataset comprised 18,039 samples. A pivot table was created (4,140 haplotypes in rows and 36 populations in columns), and Neolithic LBK data were included. Similarity between LBK and other populations was described quantitatively in two ways: (1) indicating presence or absence (1/0), i.e., whether or not the particular Neolithic haplotype was found in a given modern population, and (2) indicating the number of hits, i.e., how many times the particular haplotype was found in a given population. The 25 different LBK sequence haplotypes were sorted into clusters of noninformative (11), informative (10), and unique (4) haplotypes (Table S4). We then calculated the relative frequency of each of the shared informative vs. noninformative LBK sequence haplotypes in each of the 36 modern-day populations (Table S4). A two-tailed *z* test (Excel version 12.1, Microsoft Office) was applied to determine which population pool showed a significantly higher or lower percentage of shared informative haplotypes (Table S4). Nonparametric bootstrapping of 100 replicates for each hg per population was used to generate the confidence intervals for the percentage of hgs that are shared between all matches, informative matches, and noninformative matches. Bootstrapping was performed in Excel.version 12.1.

### Principal Component and Multidimensional Scaling Analyses

Classical and categorical PCAs and MDS were performed using the hg frequencies dataset. To avoid overpopulating graphs with 228 populations, populations were pooled into 55 groups defined by ethnicity, language, and/or geography as described in the original publications (see Table S6). To minimize statistical noise caused by very rare hgs, we considered only the following 19 hgs with average frequency above 1% in Europe and Near East: preHV, H, HV, J, T, I, N1a, K, V, W, X, U2, U3, U4, U5a, U5b, the group of African hgs (L and M1), the group of East Eurasian hgs (A, B, C, D, F, G, and Z), and the group of all other (rare) hgs. PCAs and categorical PCAs (used for the biplot graph in Figure 1, with default settings to correspond to a classical PCA) were performed and visualized using the software package SPSS Statistics 17.0. Nei's genetic distances [62] were calculated using the software program DJ, written by Yuri Seryogin (freely available at http://www.genofond.ru). The resulting distance matrix was visualized via MDS in SPSS Statistics 17.0.

### Mapping Genetic Distances

The genetic distances from two Neolithic datasets (DEB22 and LBK42) to populations in the hg frequencies dataset (pooled into 120 populations with the average sample size *n* = 196 to gain a balanced geographical coverage) were calculated using the software DJ. Distances were plotted on a geographic map of Europe using the software GeneGeo written by S. K. This software is the renewed GGMAG package previously used for gene geographical studies ([63] and references therein).

### Bayesian Serial Simcoal Analysis

We calculated population-specific pairwise genetic distances (*F*_ST_) in Arlequin version 3.5 [64], using 377-bp HVS-I sequences (np 16069–16365) assigned to one of four populations (Table S6): modern Central Europeans from the LBK core area (*n* = 1,030), modern Near Easterners (*n* = 737), LBK samples (*n* = 42), and hunter–gatherers (*n* = 20). *F*_ST_ values were estimated using the Kimura two-parameter model [65] using a gamma distribution with shape parameter of 0.205 [66].

To test whether drift can account for the high *F*_ST_ values between ancient and contemporary populations from Central Europe and the Near East we modeled three alternative population histories (Figure S2) using simulated coalescent analyses in the program BayeSSC [67],[68].

Under the null hypothesis (H_0_) we considered one large continuous Eurasian population with an effective population size ranging from 100,000 to 30 million and an exponential growth starting from a small Palaeolithic deme of 5,000 females, 300 (H_0_a) or 1,500 (H_0_b) generations ago. Hypothesis 1 (H_1_) assumes two exponentially growing populations, a Central European deme (100,000 to 12 million) and a Near Eastern deme (100,000 to 12 million), which coalesce 1,500 generations ago (37,500 y ago, assuming 25 y per generation) in an Early Upper Palaeolithic deme of 5,000 females and constant size. Here, ancient samples from hunter–gatherers and Neolithic farmers were included in the Central European deme; therefore, this model can be considered a test for genetic continuity of Central European lineages under a scenario of cultural diffusion/transmission. Alternatively, we modeled a contrasting (“demic diffusion”) scenario (H_2_), similar to H_1_ in structure but allowing for migration from the Near Eastern deme 290 generations ago. We tested a contribution of 25%, 50%, and 75% migrants from the Near Eastern to the Central European deme.

Each model was simulated initially using BayeSSC for 100,000 genealogies and a fixed mutation rate of 7.5×10^−6^ per site per generation [66]. A uniform distribution was used for priors to estimate effective population sizes at time 0 for the Central European and Near Eastern demes (Table 4). To compare the simulated and observed data, five pairwise *F*_ST_ values were chosen that reflect population differentiation between each of the two ancient samples and modern populations (Table 3). The simulated and observed *F*_ST_ values were compared within an ABC framework [69], in which the top 1% of simulations were retained. Posterior distributions for each of the parameters with a prior were assessed. ABC was performed in R version 2.11.0 using scripts freely available at http://www.stanford.edu/group/hadlylab/ssc/index.html.

To compare the goodness of fit of each model using AIC [70] given the observed data, priors were removed from the model and replaced with absolute parameter values that gave the maximum likelihood. The model was rerun in BayeSSC for 1,000 genealogies. The AIC for each model was calculated in R, and Akaike weights ω to compare the relative likelihood of each model where calculated in Excel version 12.1 [71],[72].

## Supporting Information

Dataset S1 Sequence alignments of the Derenburg individuals.
(17.75 MB PDF)

Figure S1 Multidimensional scaling plot of genetic distances based on haplogroup frequencies (alienation = 0, 1117760; stress = 0, 1053030). Population abbreviations are consistent with Figure 1, and further population details and references are listed in Table S6.
(1.05 MB TIF)

Figure S2 Demographic models and population pairwise *F*_ST_ values used in BayeSSC analyses. CE_1_, Central European deme 1; exp, exponential; HG, hunter–gatherers; M, migrants; Ne, effective population size; NE_0_, Near Eastern deme 0; r, growth rate; UP, Upper Paleolithic.
(3.00 MB TIF)

Figure S3 Map of the Neolithic graveyard Derenburg Meerenstieg II.
(1.29 MB TIF)

Table S1 Results of mtDNA coding region SNP typing using the GenoCoRe22 assay. SNPs are detected in forward orientation (L-strand) unless stated otherwise (underlined), and SNP results are reported as typed in the SBE assay. Italicized samples were discarded from further analyses. Samples were typed twice from two independent extracts except for individuals deb1 and deb2. Empty cells indicate either allelic dropout or a relative fluorescence unit value below the threshold of 50. SNP 3594_L3'4 consistently yielded relative fluorescence unit values below 50, and was not reported. Subsequent primer mixes were adjusted for the suboptimal performance of SNP3594 (Table S7).
(0.26 MB DOC)

Table S2 Results of Y chromosome SNP typing using the GenoY25 assay. SNPs are detected in forward orientation unless stated otherwise (underlined), and SNP results are reported as typed in the SBE assay.
(0.21 MB DOC)

Table S3 Quantitative real-time PCR of Neolithic Samples from Derenburg.
(0.02 MB XLS)

Table S4 Shared haplotype analyses.
(0.08 MB XLS)

Table S5 Ancient samples from other LBK sites used for population genetics analyses [19].
(0.07 MB PDF)

Table S6 Details of Neolithic and modern-day populations used for comparison.
(0.14 MB XLS)

Table S7 GenoCoRe22 and GenoY25 multiplex assay and additional Y chromosome PCR primer information.
(0.24 MB XLS)

## Abbreviations

ABC: approximate Bayesian computation
ACAD: Australian Centre for Ancient DNA
aDNA: ancient DNA
AIC: Akaike information criterion
BayeSSC: Bayesian Serial Simcoal
cal b.c.: calibrated b.c
hg: haplogroup
HVS-I: hypervariable segment I
LBK: Linear Pottery Culture
MDS: multidimensional scaling
mtDNA: mitochondrial DNA
np: nucleotide position(s)
PC: principal component
PCA: principal component analysis
qPCR: quantitative real-time PCR
SBE: single base extension
SNP: single nucleotide polymorphism
