# Supplementary material for: Ancient DNA from European Early Neolithic Farmers Reveals Their Near Eastern Affinities
Source: PLoS Biol. 2010 Nov 9;8(11):e1000536. doi: 10.1371/journal.pbio.1000536 (PMC2976717; doi:10.1371/journal.pbio.1000536)
Supplement: Dataset S1 — Sequence alignments of the Derenburg individuals. (17.75 MB PDF) [file pbio.1000536.s001.pdf]

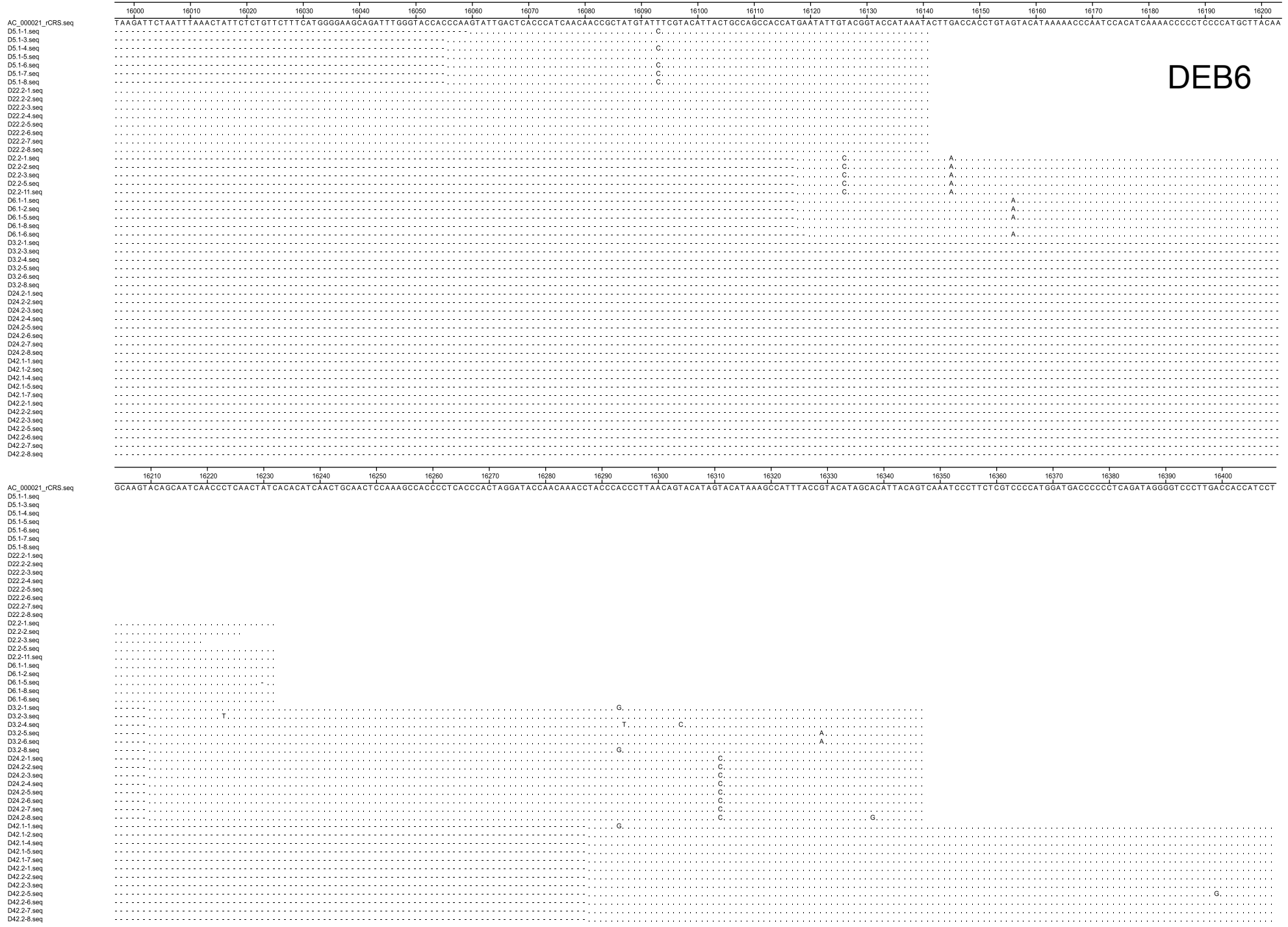
DEB6
16000 16010 16020 16030 16040 16050 16060 16070 16080 16090 16100 16110 16120 16130 16140 16150 16160 16170 16180 16190 16200
AC_000021_rCRS.seq
TAAGATTCTAATTTAAACTATTCTCTGTTCTTTCATGGGGAAGCAGATTTGGGTACCACCCAAGTATTGACTCACCCATCAACAACCGCTATGTATTTCGTACATTACTGCCAGCCACCATGAATATTGTACGGTACCATAAATACTTGACCACCTGTAGTACATAAAAACCCAATCCACATCAAAACCCCCTCCCCATGCTTACAA
D5.1-1.seq
D5.1-3.seq
D5.1-4.seq
D5.1-5.seq
D5.1-6.seq
D5.1-7.seq
D5.1-8.seq
D22.2-1.seq
D22.2-2.seq
D22.2-3.seq
D22.2-4.seq
D22.2-5.seq
D22.2-6.seq
D22.2-7.seq
D22.2-8.seq
D2.2-1.seq
D2.2-2.seq
D2.2-3.seq
D2.2-5.seq
D2.2-11.seq
D6.1-1.seq
D6.1-2.seq
D6.1-5.seq
D6.1-8.seq
D6.1-6.seq
D3.2-1.seq
D3.2-3.seq
D3.2-4.seq
D3.2-5.seq
D3.2-6.seq
D3.2-8.seq
D24.2-1.seq
D24.2-2.seq
D24.2-3.seq
D24.2-4.seq
D24.2-5.seq
D24.2-6.seq
D24.2-7.seq
D24.2-8.seq
D42.1-1.seq
D42.1-2.seq
D42.1-4.seq
D42.1-5.seq
D42.1-7.seq
D42.2-1.seq
D42.2-2.seq
D42.2-3.seq
D42.2-5.seq
D42.2-6.seq
D42.2-7.seq
D42.2-8.seq
16210 16220 16230 16240 16250 16260 16270 16280 16290 16300 16310 16320 16330 16340 16350 16360 16370 16380 16390 16400
AC_000021_rCRS.seq
GCAAGTACAGCAATCAACCCTCAACTATCACACATCAACTGCAACTCCAAAGCCACCCCTCACCCACTAGGATACCAACAAACCTACCCACCCTTAACAGTACATAGTACATAAAGCCATTTACCGTACATAGCACATTACAGTCAAATCCCTTCTCGTCCCCATGGATGACCCCCCTCAGATAGGGGTCCCTTGACCACCATCCT
D5.1-1.seq
D5.1-3.seq
D5.1-4.seq
D5.1-5.seq
D5.1-6.seq
D5.1-7.seq
D5.1-8.seq
D22.2-1.seq
D22.2-2.seq
D22.2-3.seq
D22.2-4.seq
D22.2-5.seq
D22.2-6.seq
D22.2-7.seq
D22.2-8.seq
D2.2-1.seq
D2.2-2.seq
D2.2-3.seq
D2.2-5.seq
D2.2-11.seq
D6.1-1.seq
D6.1-2.seq
D6.1-5.seq
D6.1-8.seq
D6.1-6.seq
D3.2-1.seq
D3.2-3.seq
D3.2-4.seq
D3.2-5.seq
D3.2-6.seq
D3.2-8.seq
D24.2-1.seq
D24.2-2.seq
D24.2-3.seq
D24.2-4.seq
D24.2-5.seq
D24.2-6.seq
D24.2-7.seq
D24.2-8.seq
D42.1-1.seq
D42.1-2.seq
D42.1-4.seq
D42.1-5.seq
D42.1-7.seq
D42.2-1.seq
D42.2-2.seq
D42.2-3.seq
D42.2-5.seq
D42.2-6.seq
D42.2-7.seq
D42.2-8.seq

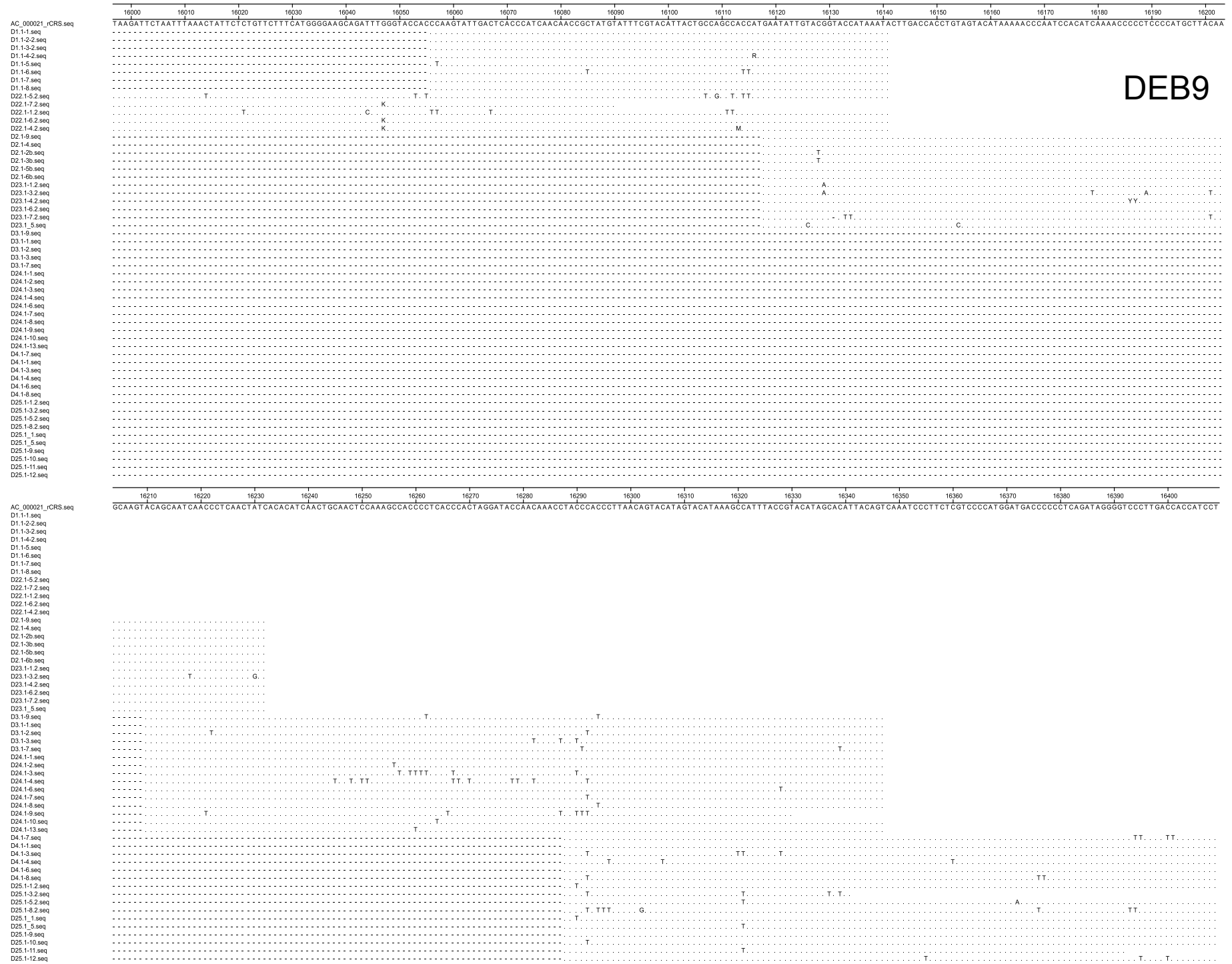
DEB9
16000 16010 16020 16030 16040 16050 16060 16070 16080 16090 16100 16110 16120 16130 16140 16150 16160 16170 16180 16190 16200
AC_000021_rCRS.seq
TAAGATTCTAATTTAAACTATTCTCTGTTCTTTCATGGGGAAGCAGATTTGGGTACCACCCAAGTATTGACTCACCCATCAACAACCGCTATGTATTTCGTACATTACTGCCAGCCACCATGAATATTGTACGGTACCATAAATACTTGACCACCTGTAGTACATAAAAACCCAATCCACATCAAAACCCCCTCCCCATGCTTACAA
16210 16220 16230 16240 16250 16260 16270 16280 16290 16300 16310 16320 16330 16340 16350 16360 16370 16380 16390 16400
AC_000021_rCRS.seq
GCAAGTACAGCAATCAACCCTCAACTATCACACATCAACTGCAACTCCAAAGCCACCCCTCACCCACTAGGATACCAACAAACCTACCCACCCTTAACAGTACATAGTACATAAAGCCATTTACCGTACATAGCACATTACAGTCAAATCCCTTCTCGTCCCCATGGATGACCCCCCTCAGATAGGGGTCCCTTGACCACCATCCT
D1.1-1.seq
D1.1-2-2.seq
D1.1-3-2.seq
D1.1-4-2.seq
D1.1-5.seq
D1.1-6.seq
D1.1-7.seq
D1.1-8.seq
D22.1-5.2.seq
D22.1-7.2.seq
D22.1-1.2.seq
D22.1-6.2.seq
D22.1-4.2.seq
D2.1-9.seq
D2.1-4.seq
D2.1-2b.seq
D2.1-3b.seq
D2.1-5b.seq
D2.1-6b.seq
D23.1-1.2.seq
D23.1-3.2.seq
D23.1-4.2.seq
D23.1-6.2.seq
D23.1-7.2.seq
D23.1_5.seq
D3.1-9.seq
D3.1-1.seq
D3.1-2.seq
D3.1-3.seq
D3.1-7.seq
D24.1-1.seq
D24.1-2.seq
D24.1-3.seq
D24.1-4.seq
D24.1-6.seq
D24.1-7.seq
D24.1-8.seq
D24.1-9.seq
D24.1-10.seq
D24.1-13.seq
D4.1-7.seq
D4.1-1.seq
D4.1-3.seq
D4.1-4.seq
D4.1-6.seq
D4.1-8.seq
D25.1-1.2.seq
D25.1-3.2.seq
D25.1-5.2.seq
D25.1-8.2.seq
D25.1_1.seq
D25.1_5.seq
D25.1-9.seq
D25.1-10.seq
D25.1-11.seq
D25.1-12.seq

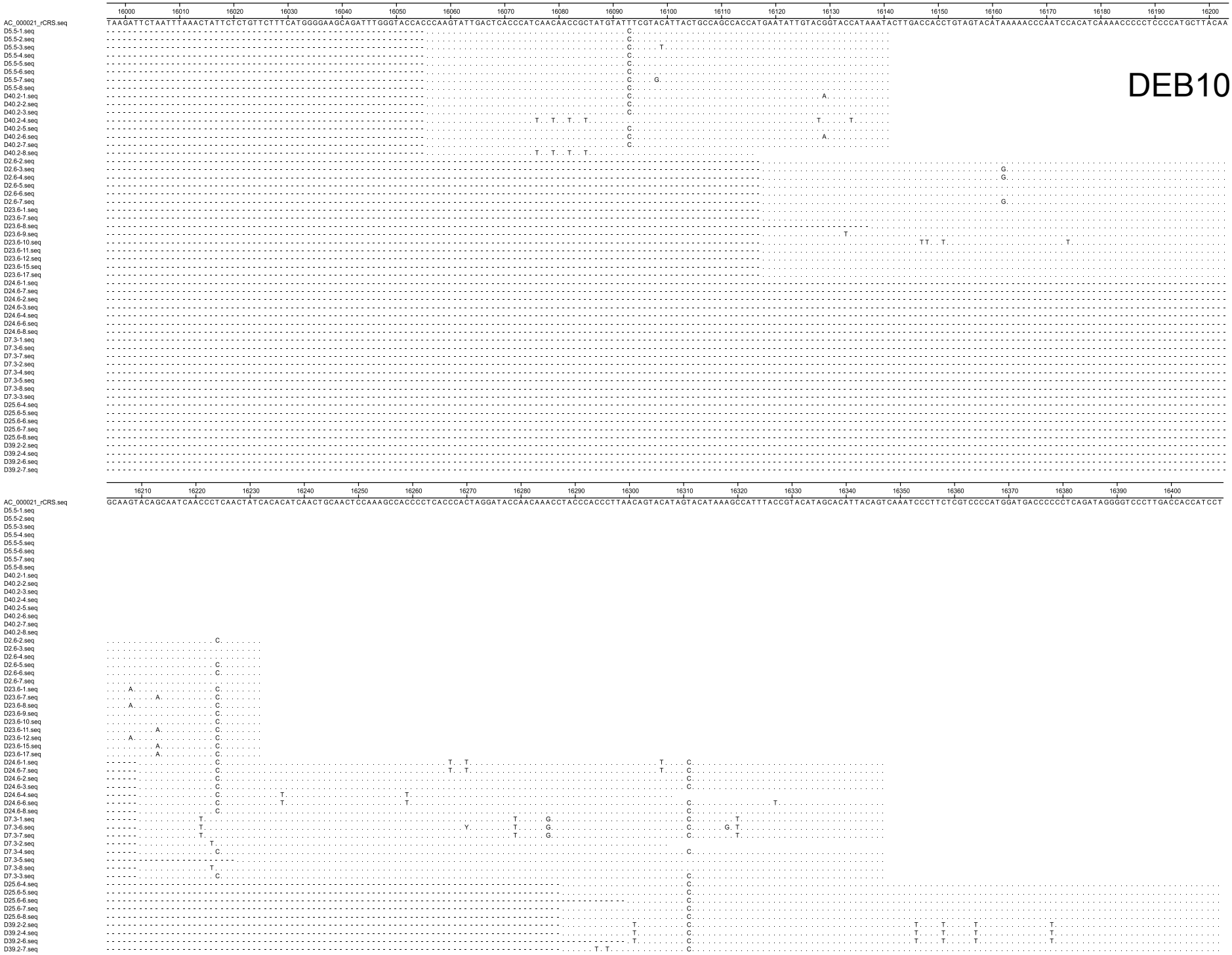
DEB10
16000
16010
16020
16030
16040
16050
16060
16070
16080
16090
16100
16110
16120
16130
16140
16150
16160
16170
16180
16190
16200
AC_000021_rCRS.seq
TAAGATTCTAATTTAAACTATTCTCTGTTCTTTCATGGGGAAGCAGATTTGGGTACCACCCAAGTATTGACTCACCCATCAACAACCGCTATGTATTTCGTACATTACTGCCAGCCACCATGAATATTGTACGGTACCATAAATACTTGACCACCTGTAGTACATAAAAACCCAATCCACATCAAAACCCCCTCCCCATGCTTACAA
D5.5-1.seq
D5.5-2.seq
D5.5-3.seq
D5.5-4.seq
D5.5-5.seq
D5.5-6.seq
D5.5-7.seq
D5.5-8.seq
D40.2-1.seq
D40.2-2.seq
D40.2-3.seq
D40.2-4.seq
D40.2-5.seq
D40.2-6.seq
D40.2-7.seq
D40.2-8.seq
D2.6-2.seq
D2.6-3.seq
D2.6-4.seq
D2.6-5.seq
D2.6-6.seq
D2.6-7.seq
D23.6-1.seq
D23.6-7.seq
D23.6-8.seq
D23.6-9.seq
D23.6-10.seq
D23.6-11.seq
D23.6-12.seq
D23.6-15.seq
D23.6-17.seq
D24.6-1.seq
D24.6-7.seq
D24.6-2.seq
D24.6-3.seq
D24.6-4.seq
D24.6-6.seq
D24.6-8.seq
D7.3-1.seq
D7.3-6.seq
D7.3-7.seq
D7.3-2.seq
D7.3-4.seq
D7.3-5.seq
D7.3-8.seq
D7.3-3.seq
D25.6-4.seq
D25.6-5.seq
D25.6-6.seq
D25.6-7.seq
D25.6-8.seq
D39.2-2.seq
D39.2-4.seq
D39.2-6.seq
D39.2-7.seq
16210
16220
16230
16240
16250
16260
16270
16280
16290
16300
16310
16320
16330
16340
16350
16360
16370
16380
16390
16400
AC_000021_rCRS.seq
GCAAGTACAGCAATCAACCCTCAACTATCACACATCAACTGCAACTCCAAAGCCACCCCTCACCCACTAGGATACCAACAAACCTACCCACCCTTAACAGTACATAGTACATAAAGCCATTTACCGTACATAGCACATTACAGTCAAATCCCTTCTCGTCCCCATGGATGACCCCCCTCAGATAGGGGTCCCTTGACCACCATCCT
D5.5-1.seq
D5.5-2.seq
D5.5-3.seq
D5.5-4.seq
D5.5-5.seq
D5.5-6.seq
D5.5-7.seq
D5.5-8.seq
D40.2-1.seq
D40.2-2.seq
D40.2-3.seq
D40.2-4.seq
D40.2-5.seq
D40.2-6.seq
D40.2-7.seq
D40.2-8.seq
D2.6-2.seq
D2.6-3.seq
D2.6-4.seq
D2.6-5.seq
D2.6-6.seq
D2.6-7.seq
D23.6-1.seq
D23.6-7.seq
D23.6-8.seq
D23.6-9.seq
D23.6-10.seq
D23.6-11.seq
D23.6-12.seq
D23.6-15.seq
D23.6-17.seq
D24.6-1.seq
D24.6-7.seq
D24.6-2.seq
D24.6-3.seq
D24.6-4.seq
D24.6-6.seq
D24.6-8.seq
D7.3-1.seq
D7.3-6.seq
D7.3-7.seq
D7.3-2.seq
D7.3-4.seq
D7.3-5.seq
D7.3-8.seq
D7.3-3.seq
D25.6-4.seq
D25.6-5.seq
D25.6-6.seq
D25.6-7.seq
D25.6-8.seq
D39.2-2.seq
D39.2-4.seq
D39.2-6.seq
D39.2-7.seq

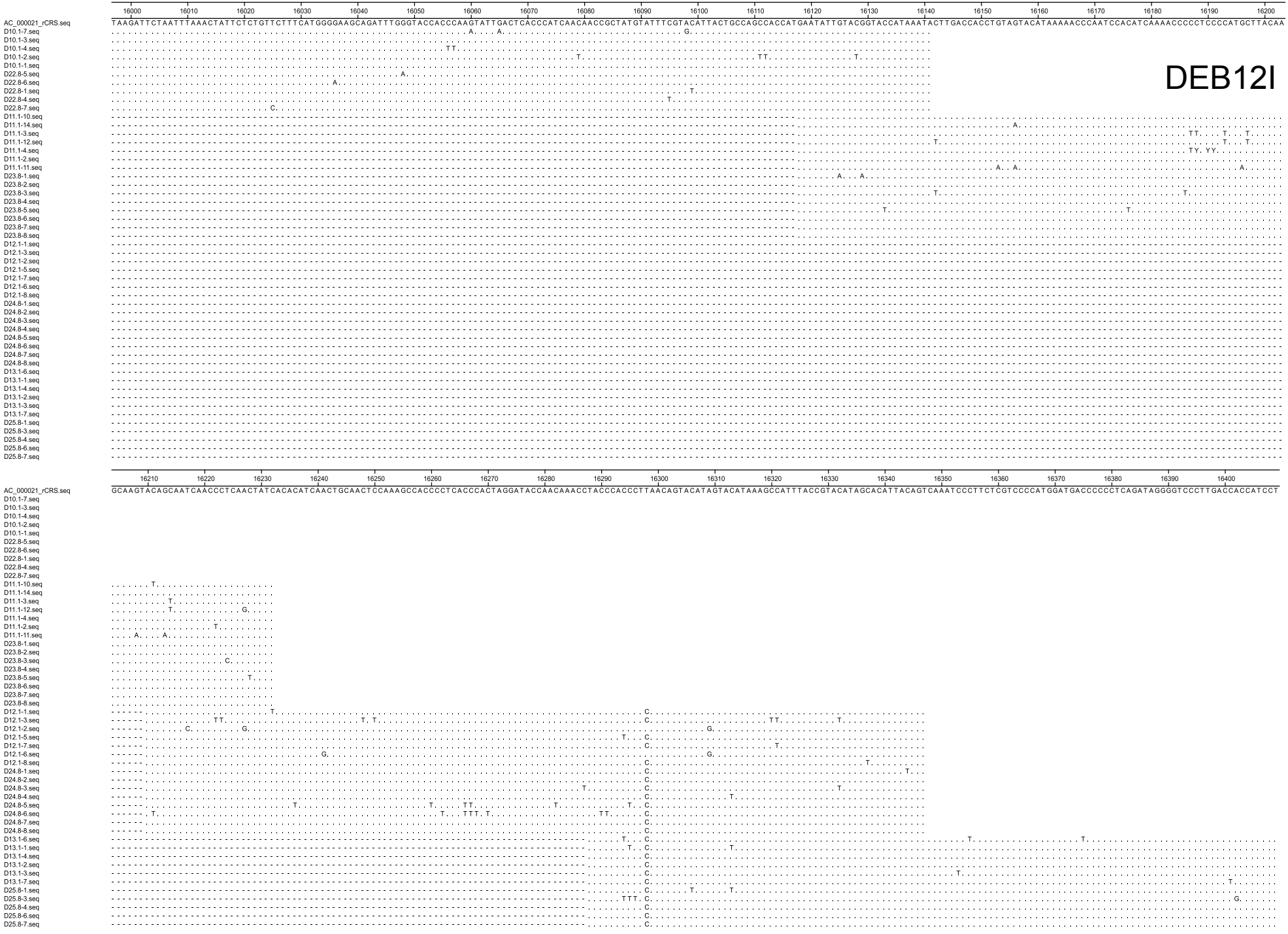
DEB12I
16000
16010
16020
16030
16040
16050
16060
16070
16080
16090
16100
16110
16120
16130
16140
16150
16160
16170
16180
16190
16200
AC_000021_rCRS.seq
TAAGATTCTAATTTAAACTATTCTCTGTTCTTTCATGGGGAAGCAGATTTGGGTACCACCCAAGTATTGACTCACCCATCAACAACCGCTATGTATTTCGTACATTACTGCCAGCCACCATGAATATTGTACGGTACCATAAATACTTGACCACCTGTAGTACATAAAAACCCAATCCACATCAAAACCCCCTCCCCATGCTTACAA
D10.1-7.seq
D10.1-3.seq
D10.1-4.seq
D10.1-2.seq
D10.1-1.seq
D22.8-5.seq
D22.8-6.seq
D22.8-1.seq
D22.8-4.seq
D22.8-7.seq
D11.1-10.seq
D11.1-14.seq
D11.1-3.seq
D11.1-12.seq
D11.1-4.seq
D11.1-2.seq
D11.1-11.seq
D23.8-1.seq
D23.8-2.seq
D23.8-3.seq
D23.8-4.seq
D23.8-5.seq
D23.8-6.seq
D23.8-7.seq
D23.8-8.seq
D12.1-1.seq
D12.1-3.seq
D12.1-2.seq
D12.1-5.seq
D12.1-7.seq
D12.1-6.seq
D12.1-8.seq
D24.8-1.seq
D24.8-2.seq
D24.8-3.seq
D24.8-4.seq
D24.8-5.seq
D24.8-6.seq
D24.8-7.seq
D24.8-8.seq
D13.1-6.seq
D13.1-1.seq
D13.1-4.seq
D13.1-2.seq
D13.1-3.seq
D13.1-7.seq
D25.8-1.seq
D25.8-3.seq
D25.8-4.seq
D25.8-6.seq
D25.8-7.seq
16210
16220
16230
16240
16250
16260
16270
16280
16290
16300
16310
16320
16330
16340
16350
16360
16370
16380
16390
16400
AC_000021_rCRS.seq
GCAAGTACAGCAATCAACCCTCAACTATCACACATCAACTGCAACTCCAAAGCCACCCCTCACCCACTAGGATACCAACAAACCTACCCACCCTTAACAGTACATAGTACATAAAGCCATTTACCGTACATAGCACATTACAGTCAAATCCCTTCTCGTCCCCATGGATGACCCCCCTCAGATAGGGGTCCCTTGACCACCATCCT
D10.1-7.seq
D10.1-3.seq
D10.1-4.seq
D10.1-2.seq
D10.1-1.seq
D22.8-5.seq
D22.8-6.seq
D22.8-1.seq
D22.8-4.seq
D22.8-7.seq
D11.1-10.seq
D11.1-14.seq
D11.1-3.seq
D11.1-12.seq
D11.1-4.seq
D11.1-2.seq
D11.1-11.seq
D23.8-1.seq
D23.8-2.seq
D23.8-3.seq
D23.8-4.seq
D23.8-5.seq
D23.8-6.seq
D23.8-7.seq
D23.8-8.seq
D12.1-1.seq
D12.1-3.seq
D12.1-2.seq
D12.1-5.seq
D12.1-7.seq
D12.1-6.seq
D12.1-8.seq
D24.8-1.seq
D24.8-2.seq
D24.8-3.seq
D24.8-4.seq
D24.8-5.seq
D24.8-6.seq
D24.8-7.seq
D24.8-8.seq
D13.1-6.seq
D13.1-1.seq
D13.1-4.seq
D13.1-2.seq
D13.1-3.seq
D13.1-7.seq
D25.8-1.seq
D25.8-3.seq
D25.8-4.seq
D25.8-6.seq
D25.8-7.seq

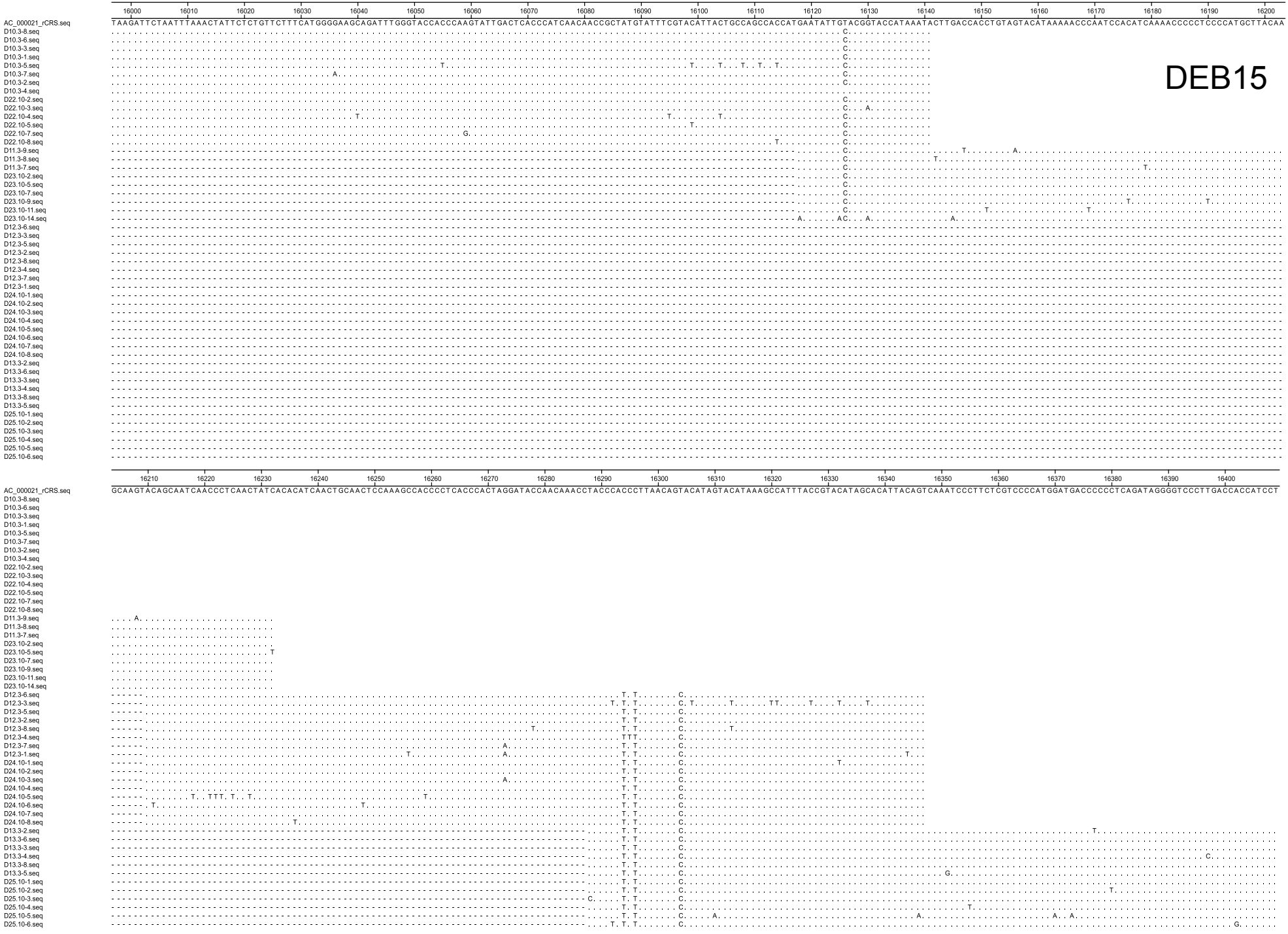
DEB15
16000 16010 16020 16030 16040 16050 16060 16070 16080 16090 16100 16110 16120 16130 16140 16150 16160 16170 16180 16190 16200
AC_000021_rCRS.seq
16210 16220 16230 16240 16250 16260 16270 16280 16290 16300 16310 16320 16330 16340 16350 16360 16370 16380 16390 16400
AC_000021_rCRS.seq

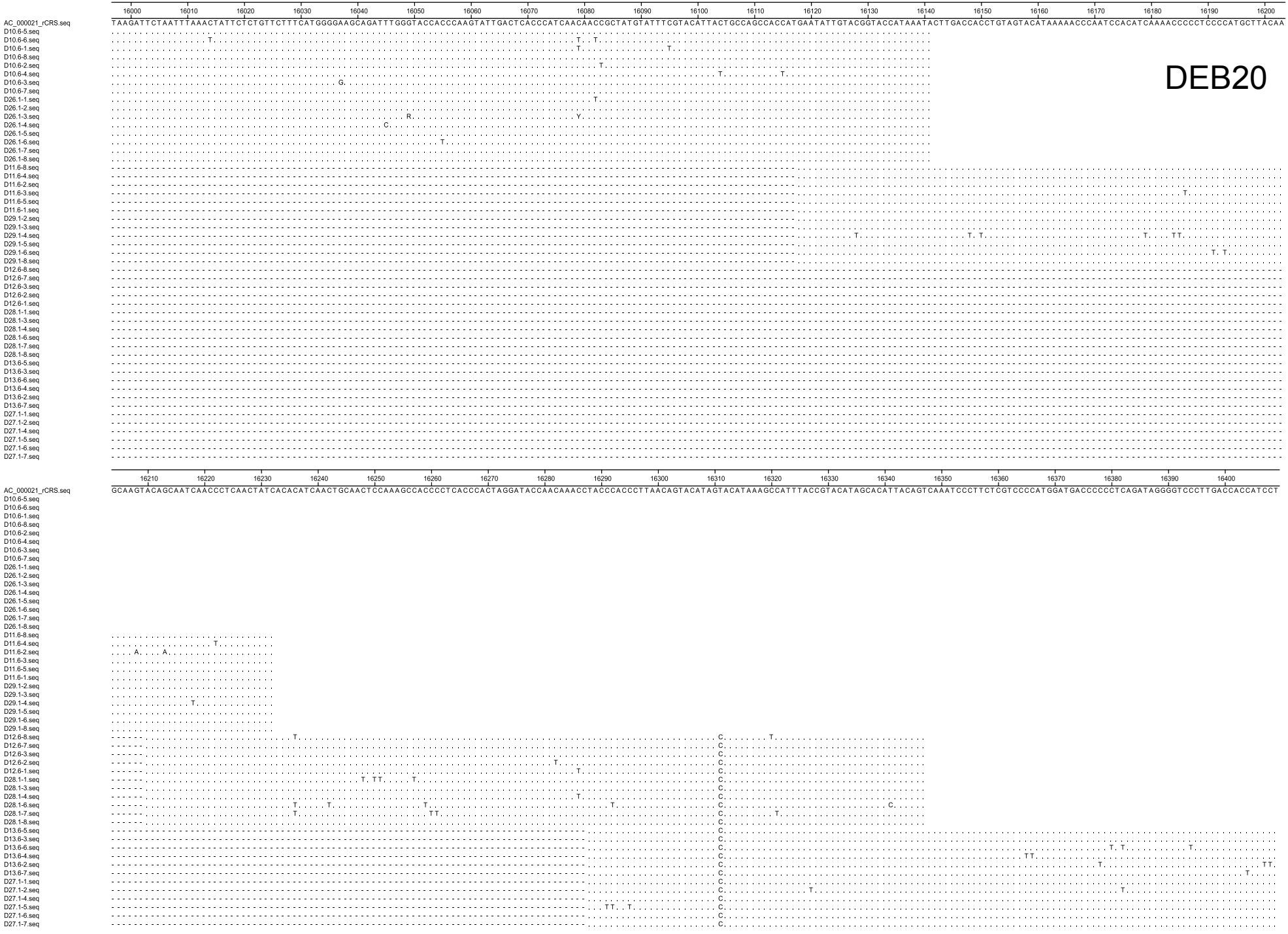
DEB20
AC_000021_rCRS.seq
D10.6-5.seq
D10.6-6.seq
D10.6-1.seq
D10.6-8.seq
D10.6-2.seq
D10.6-4.seq
D10.6-3.seq
D10.6-7.seq
D26.1-1.seq
D26.1-2.seq
D26.1-3.seq
D26.1-4.seq
D26.1-5.seq
D26.1-6.seq
D26.1-7.seq
D26.1-8.seq
D11.6-8.seq
D11.6-4.seq
D11.6-2.seq
D11.6-3.seq
D11.6-5.seq
D11.6-1.seq
D29.1-2.seq
D29.1-3.seq
D29.1-4.seq
D29.1-5.seq
D29.1-6.seq
D29.1-8.seq
D12.6-8.seq
D12.6-7.seq
D12.6-3.seq
D12.6-2.seq
D12.6-1.seq
D28.1-1.seq
D28.1-3.seq
D28.1-4.seq
D28.1-6.seq
D28.1-7.seq
D28.1-8.seq
D13.6-5.seq
D13.6-3.seq
D13.6-6.seq
D13.6-4.seq
D13.6-2.seq
D13.6-7.seq
D27.1-1.seq
D27.1-2.seq
D27.1-4.seq
D27.1-5.seq
D27.1-6.seq
D27.1-7.seq
16000
16010
16020
16030
16040
16050
16060
16070
16080
16090
16100
16110
16120
16130
16140
16150
16160
16170
16180
16190
16200
TAAGATTCTAATTTAAACTATTCTCTGTTCTTTCATGGGGAAGCAGATTTGGGTACCACCCAAGTATTGACTCACCCATCAACAACCGCTATGTATTTCGTACATTACTGCCAGCCACCATGAATATTGTACGGTACCATAAATACTTGACCACCTGTAGTACATAAAAACCCAATCCACATCAAAACCCCCTCCCCATGCTTACAA
16210
16220
16230
16240
16250
16260
16270
16280
16290
16300
16310
16320
16330
16340
16350
16360
16370
16380
16390
16400
GCAAGTACAGCAATCAACCCTCAACTATCACACATCAACTGCAACTCCAAAGCCACCCCTCACCCACTAGGATACCAACAAACCTACCCACCCTTAACAGTACATAGTACATAAAGCCATTTACCGTACATAGCACATTACAGTCAAATCCCTTCTCGTCCCCATGGATGACCCCCCTCAGATAGGGGTCCCTTGACCACCATCCT

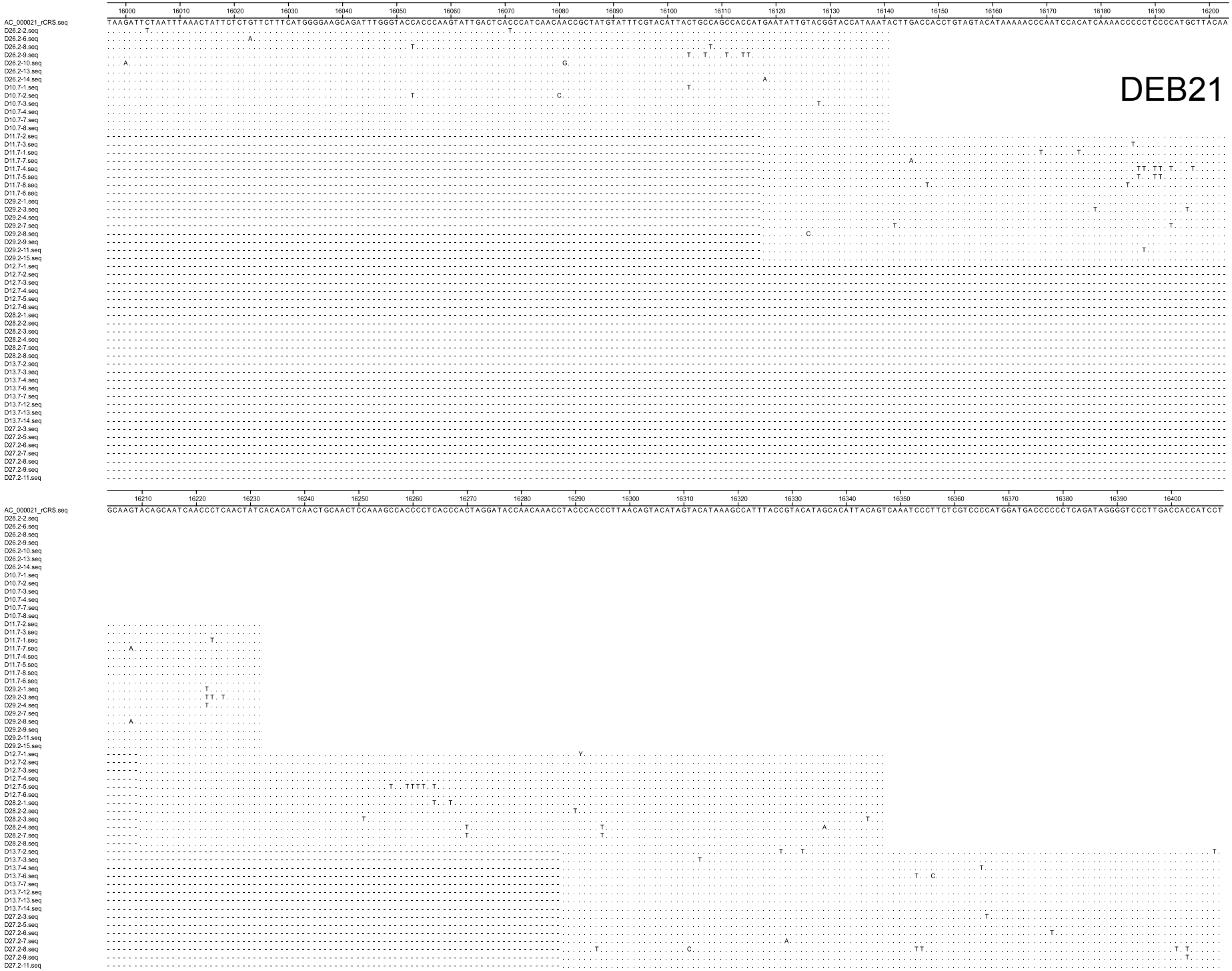
DEB21
16000
16010
16020
16030
16040
16050
16060
16070
16080
16090
16100
16110
16120
16130
16140
16150
16160
16170
16180
16190
16200
AC_000021_rCRS.seq
D26.2-2.seq
D26.2-6.seq
D26.2-8.seq
D26.2-9.seq
D26.2-10.seq
D26.2-13.seq
D26.2-14.seq
D10.7-1.seq
D10.7-2.seq
D10.7-3.seq
D10.7-4.seq
D10.7-7.seq
D10.7-8.seq
D11.7-2.seq
D11.7-3.seq
D11.7-1.seq
D11.7-7.seq
D11.7-4.seq
D11.7-5.seq
D11.7-8.seq
D11.7-6.seq
D29.2-1.seq
D29.2-3.seq
D29.2-4.seq
D29.2-7.seq
D29.2-8.seq
D29.2-9.seq
D29.2-11.seq
D29.2-15.seq
D12.7-1.seq
D12.7-2.seq
D12.7-3.seq
D12.7-4.seq
D12.7-5.seq
D12.7-6.seq
D28.2-1.seq
D28.2-2.seq
D28.2-3.seq
D28.2-4.seq
D28.2-7.seq
D28.2-8.seq
D13.7-2.seq
D13.7-3.seq
D13.7-4.seq
D13.7-6.seq
D13.7-7.seq
D13.7-12.seq
D13.7-13.seq
D13.7-14.seq
D27.2-3.seq
D27.2-5.seq
D27.2-6.seq
D27.2-7.seq
D27.2-8.seq
D27.2-9.seq
D27.2-11.seq
16210
16220
16230
16240
16250
16260
16270
16280
16290
16300
16310
16320
16330
16340
16350
16360
16370
16380
16390
16400

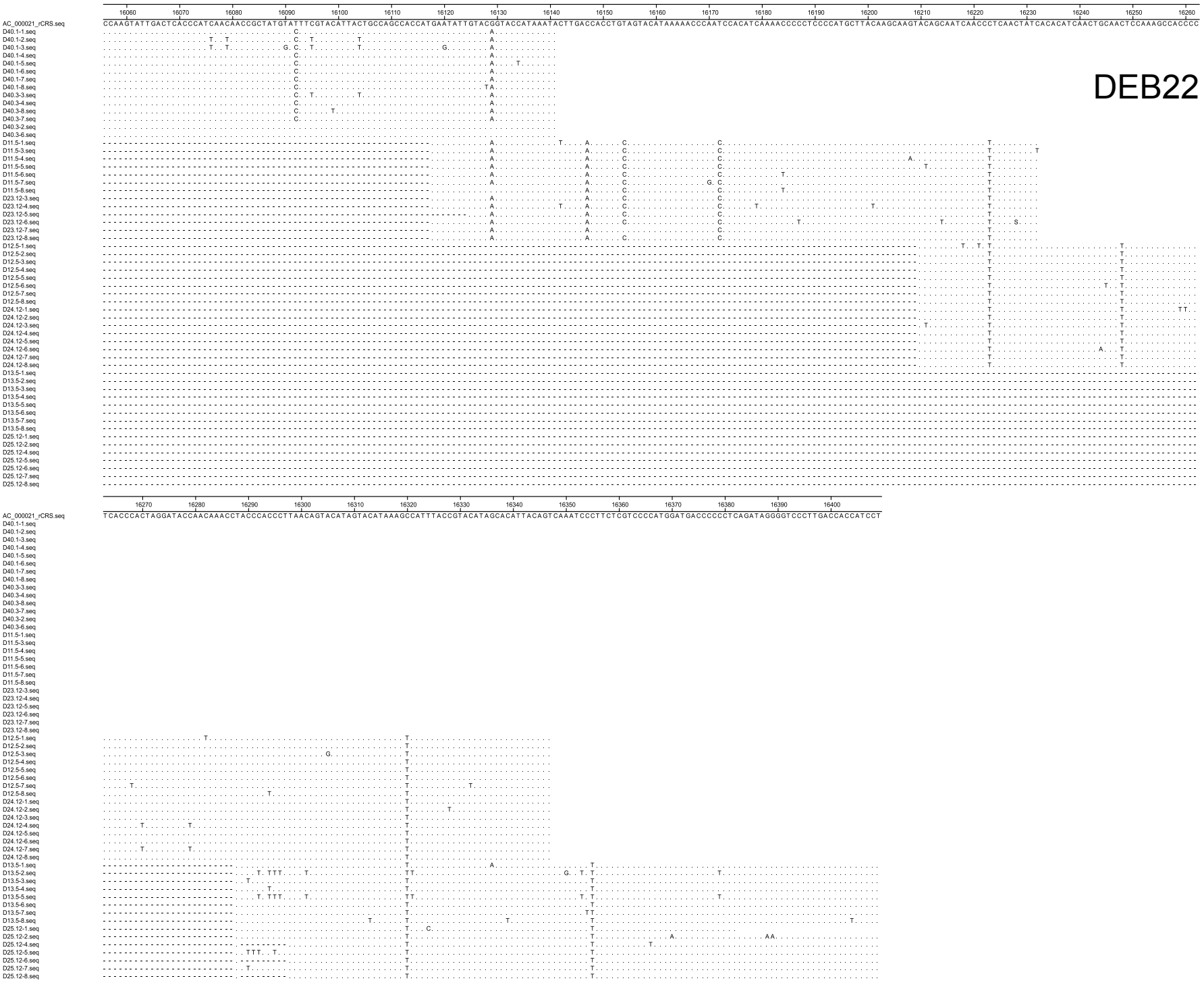
DEB22
16060
16070
16080
16090
16100
16110
16120
16130
16140
16150
16160
16170
16180
16190
16200
16210
16220
16230
16240
16250
16260
16270
16280
16290
16300
16310
16320
16330
16340
16350
16360
16370
16380
16390
16400
AC_000021_rCRS.seq
D40.1-1.seq
D40.1-2.seq
D40.1-3.seq
D40.1-4.seq
D40.1-5.seq
D40.1-6.seq
D40.1-7.seq
D40.1-8.seq
D40.3-3.seq
D40.3-4.seq
D40.3-8.seq
D40.3-7.seq
D40.3-2.seq
D40.3-6.seq
D11.5-1.seq
D11.5-3.seq
D11.5-4.seq
D11.5-5.seq
D11.5-6.seq
D11.5-7.seq
D11.5-8.seq
D23.12-3.seq
D23.12-4.seq
D23.12-5.seq
D23.12-6.seq
D23.12-7.seq
D23.12-8.seq
D12.5-1.seq
D12.5-2.seq
D12.5-3.seq
D12.5-4.seq
D12.5-5.seq
D12.5-6.seq
D12.5-7.seq
D12.5-8.seq
D24.12-1.seq
D24.12-2.seq
D24.12-3.seq
D24.12-4.seq
D24.12-5.seq
D24.12-6.seq
D24.12-7.seq
D24.12-8.seq
D13.5-1.seq
D13.5-2.seq
D13.5-3.seq
D13.5-4.seq
D13.5-5.seq
D13.5-6.seq
D13.5-7.seq
D13.5-8.seq
D25.12-1.seq
D25.12-2.seq
D25.12-4.seq
D25.12-5.seq
D25.12-6.seq
D25.12-7.seq
D25.12-8.seq

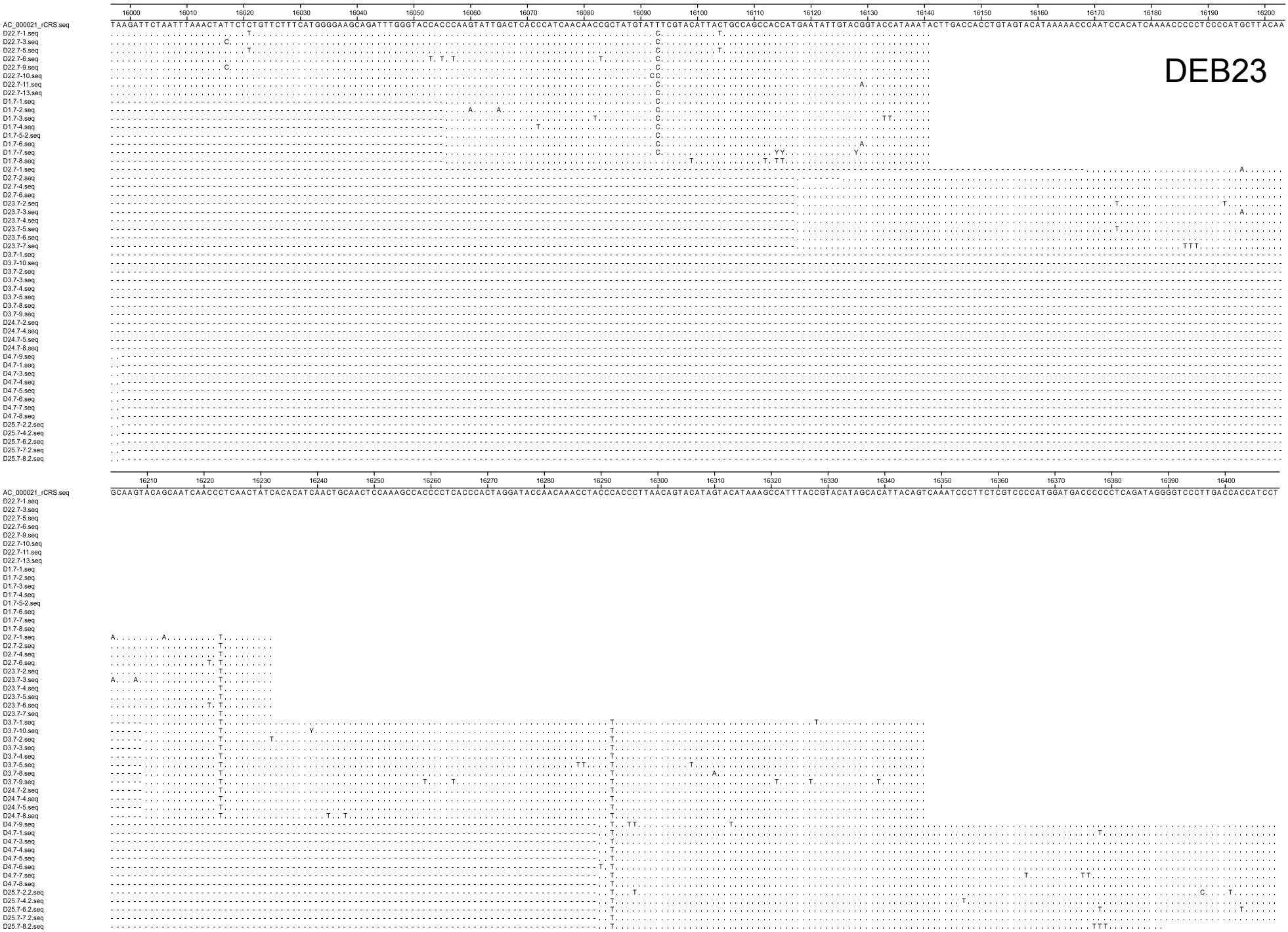
DEB23

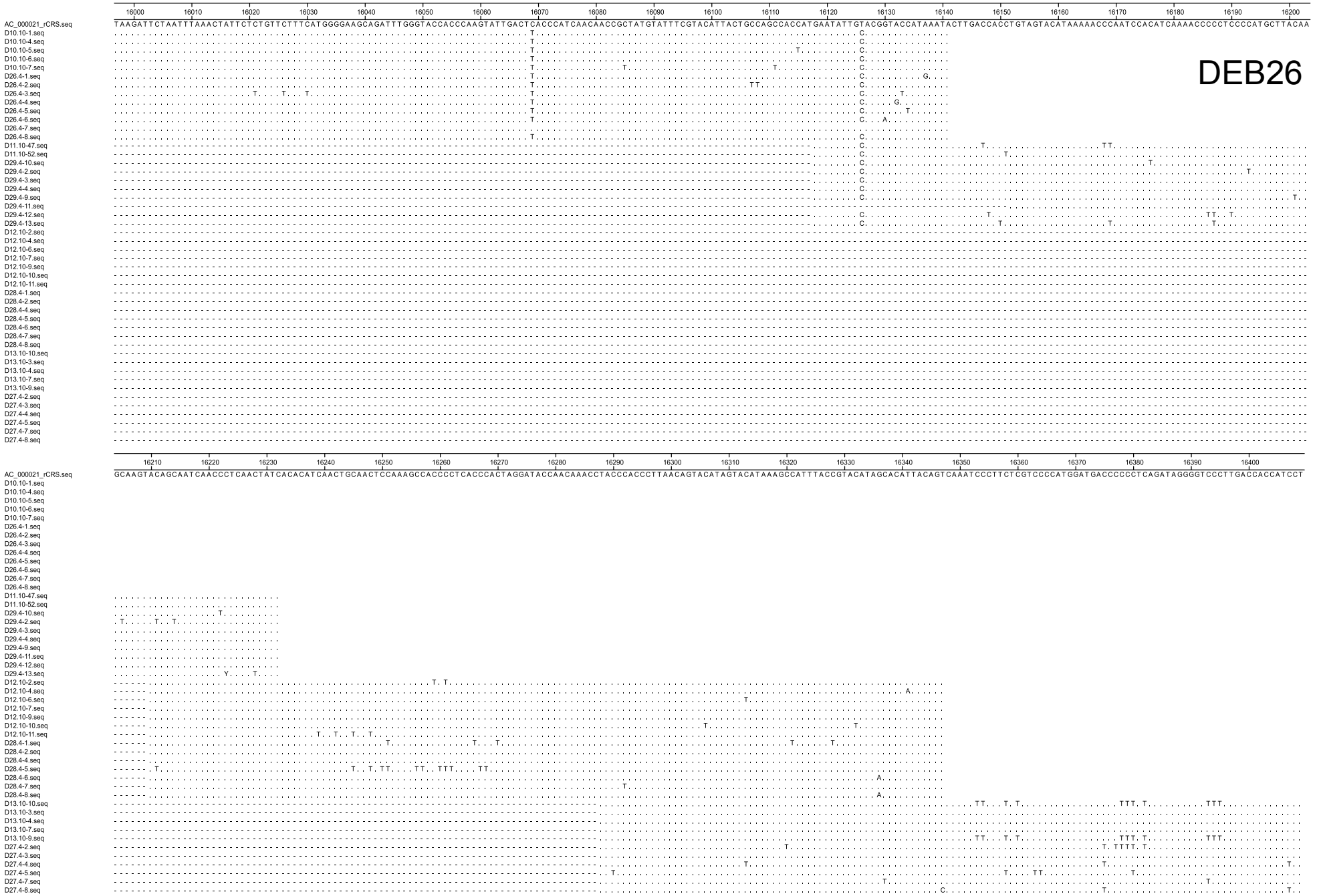
DEB26
16000 16010 16020 16030 16040 16050 16060 16070 16080 16090 16100 16110 16120 16130 16140 16150 16160 16170 16180 16190 16200
AC_000021_rCRS.seq
D10.10-1.seq
D10.10-4.seq
D10.10-5.seq
D10.10-6.seq
D10.10-7.seq
D26.4-1.seq
D26.4-2.seq
D26.4-3.seq
D26.4-4.seq
D26.4-5.seq
D26.4-6.seq
D26.4-7.seq
D26.4-8.seq
D11.10-47.seq
D11.10-52.seq
D29.4-10.seq
D29.4-2.seq
D29.4-3.seq
D29.4-4.seq
D29.4-9.seq
D29.4-11.seq
D29.4-12.seq
D29.4-13.seq
D12.10-2.seq
D12.10-4.seq
D12.10-6.seq
D12.10-7.seq
D12.10-9.seq
D12.10-10.seq
D12.10-11.seq
D28.4-1.seq
D28.4-2.seq
D28.4-4.seq
D28.4-5.seq
D28.4-6.seq
D28.4-7.seq
D28.4-8.seq
D13.10-10.seq
D13.10-3.seq
D13.10-4.seq
D13.10-7.seq
D13.10-9.seq
D27.4-2.seq
D27.4-3.seq
D27.4-4.seq
D27.4-5.seq
D27.4-7.seq
D27.4-8.seq
16210 16220 16230 16240 16250 16260 16270 16280 16290 16300 16310 16320 16330 16340 16350 16360 16370 16380 16390 16400

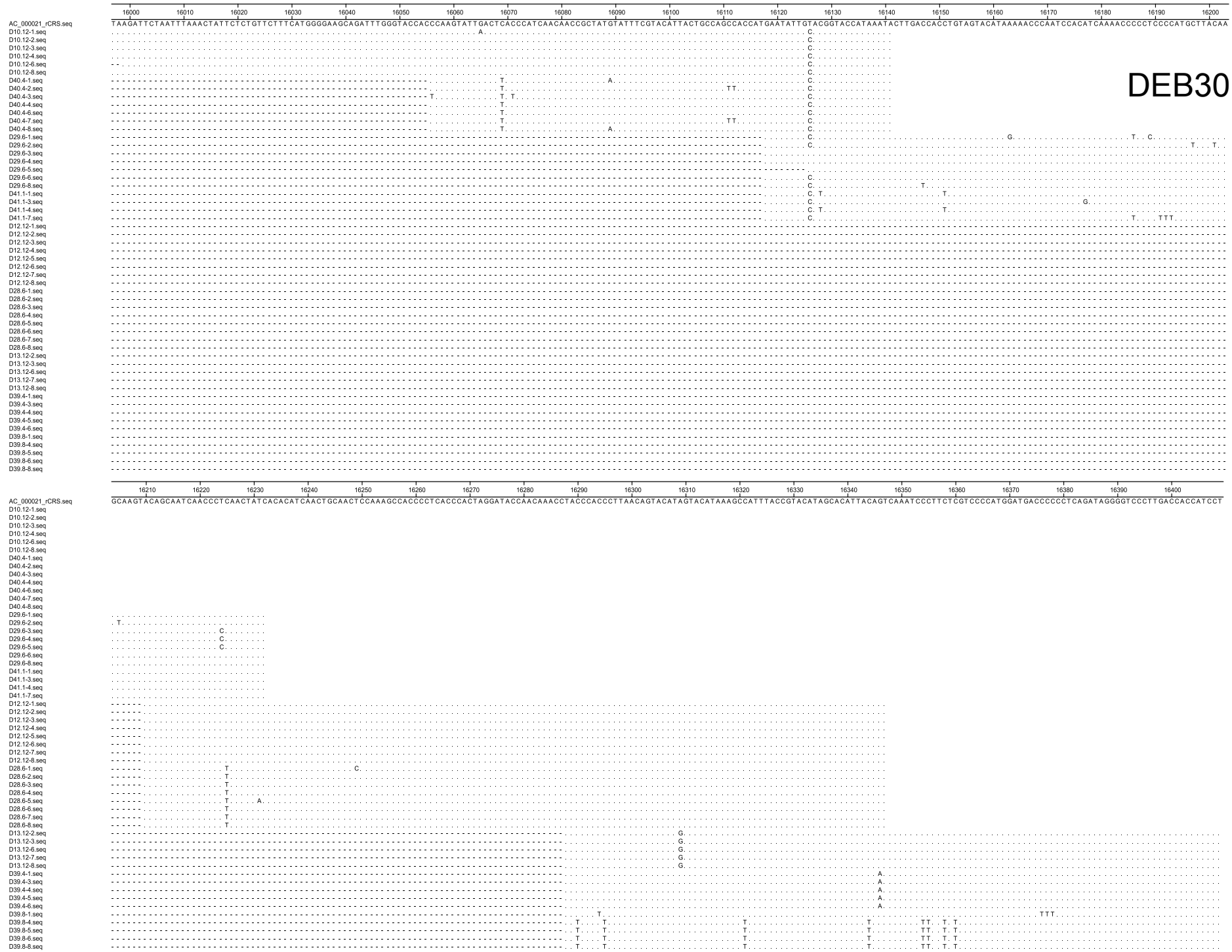
DEB30
AC_000021_rCRS.seq
D10.12-1.seq
D10.12-2.seq
D10.12-3.seq
D10.12-4.seq
D10.12-6.seq
D10.12-8.seq
D40.4-1.seq
D40.4-2.seq
D40.4-3.seq
D40.4-4.seq
D40.4-6.seq
D40.4-7.seq
D40.4-8.seq
D29.6-1.seq
D29.6-2.seq
D29.6-3.seq
D29.6-4.seq
D29.6-5.seq
D29.6-6.seq
D29.6-8.seq
D41.1-1.seq
D41.1-3.seq
D41.1-4.seq
D41.1-7.seq
D12.12-1.seq
D12.12-2.seq
D12.12-3.seq
D12.12-4.seq
D12.12-5.seq
D12.12-6.seq
D12.12-7.seq
D12.12-8.seq
D28.6-1.seq
D28.6-2.seq
D28.6-3.seq
D28.6-4.seq
D28.6-5.seq
D28.6-6.seq
D28.6-7.seq
D28.6-8.seq
D13.12-2.seq
D13.12-3.seq
D13.12-6.seq
D13.12-7.seq
D13.12-8.seq
D39.4-1.seq
D39.4-3.seq
D39.4-4.seq
D39.4-5.seq
D39.4-6.seq
D39.8-1.seq
D39.8-4.seq
D39.8-5.seq
D39.8-6.seq
D39.8-8.seq
16000
16010
16020
16030
16040
16050
16060
16070
16080
16090
16100
16110
16120
16130
16140
16150
16160
16170
16180
16190
16200
TAAGATTCTAATTTAAACTATTCTCTGTTCTTTCATGGGGAAGCAGATTTGGGTACCACCCAAGTATTGACTCACCCATCAACAACCGCTATGTATTTCGTACATTACTGCCAGCCACCATGAATATTGTACGGTACCATAAATACTTGACCACCTGTAGTACATAAAAACCCAATCCACATCAAAACCCCCTCCCCATGCTTACAA
16210
16220
16230
16240
16250
16260
16270
16280
16290
16300
16310
16320
16330
16340
16350
16360
16370
16380
16390
16400
GCAAGTACAGCAATCAACCCTCAACTATCACACATCAACTGCAACTCCAAAGCCACCCCTCACCCACTAGGATACCAACAAACCTACCCACCCTTAACAGTACATAGTACATAAAGCCATTTACCGTACATAGCACATTACAGTCAAATCCCTTCTCGTCCCCATGGATGACCCCCCTCAGATAGGGGTCCCTTGACCACCATCCT

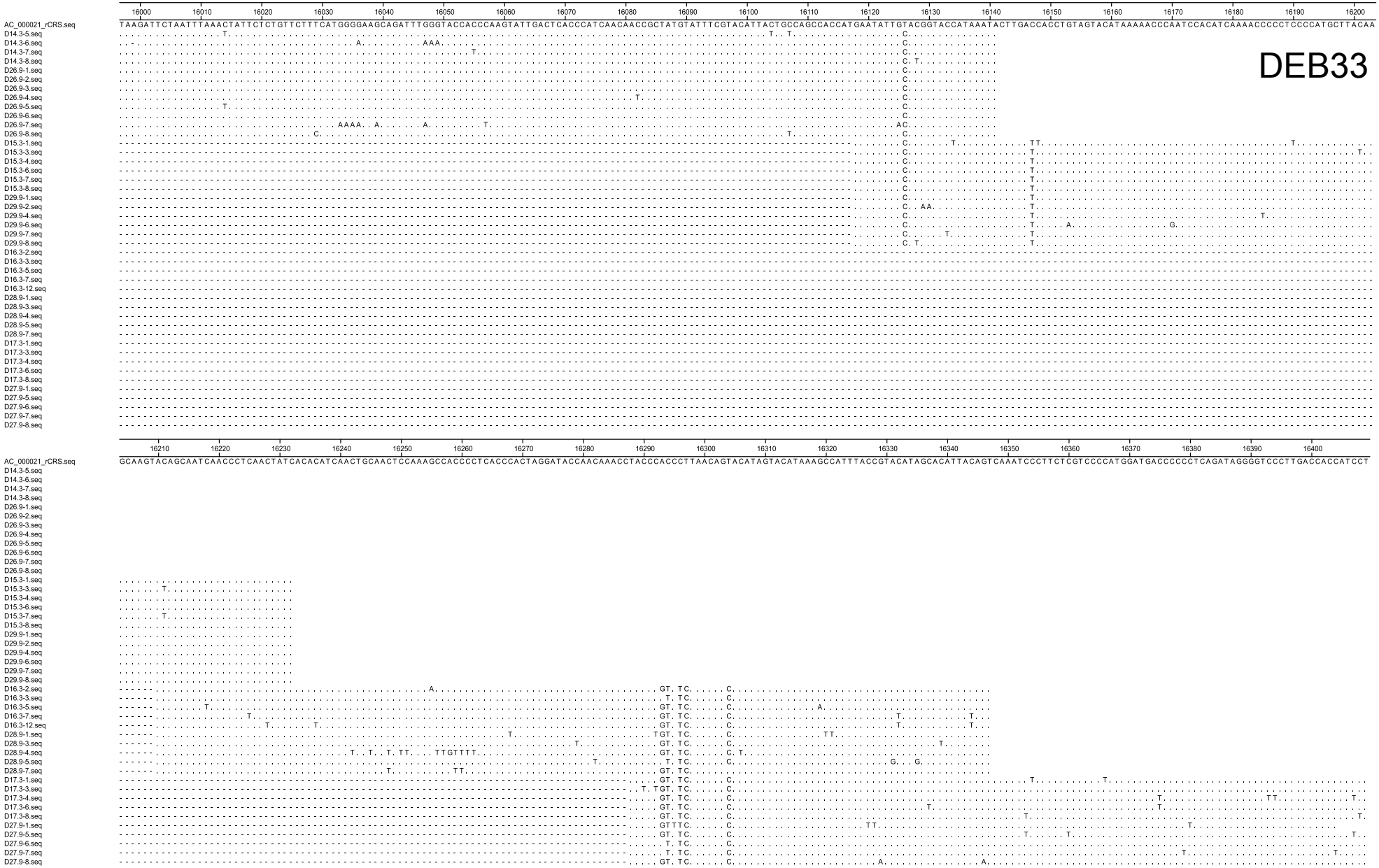
DEB33
AC_000021_rCRS.seq
D14.3-5.seq
D14.3-6.seq
D14.3-7.seq
D14.3-8.seq
D26.9-1.seq
D26.9-2.seq
D26.9-3.seq
D26.9-4.seq
D26.9-5.seq
D26.9-6.seq
D26.9-7.seq
D26.9-8.seq
D15.3-1.seq
D15.3-3.seq
D15.3-4.seq
D15.3-6.seq
D15.3-7.seq
D15.3-8.seq
D29.9-1.seq
D29.9-2.seq
D29.9-4.seq
D29.9-6.seq
D29.9-7.seq
D29.9-8.seq
D16.3-2.seq
D16.3-3.seq
D16.3-5.seq
D16.3-7.seq
D16.3-12.seq
D28.9-1.seq
D28.9-3.seq
D28.9-4.seq
D28.9-5.seq
D28.9-7.seq
D17.3-1.seq
D17.3-3.seq
D17.3-4.seq
D17.3-6.seq
D17.3-8.seq
D27.9-1.seq
D27.9-5.seq
D27.9-6.seq
D27.9-7.seq
D27.9-8.seq

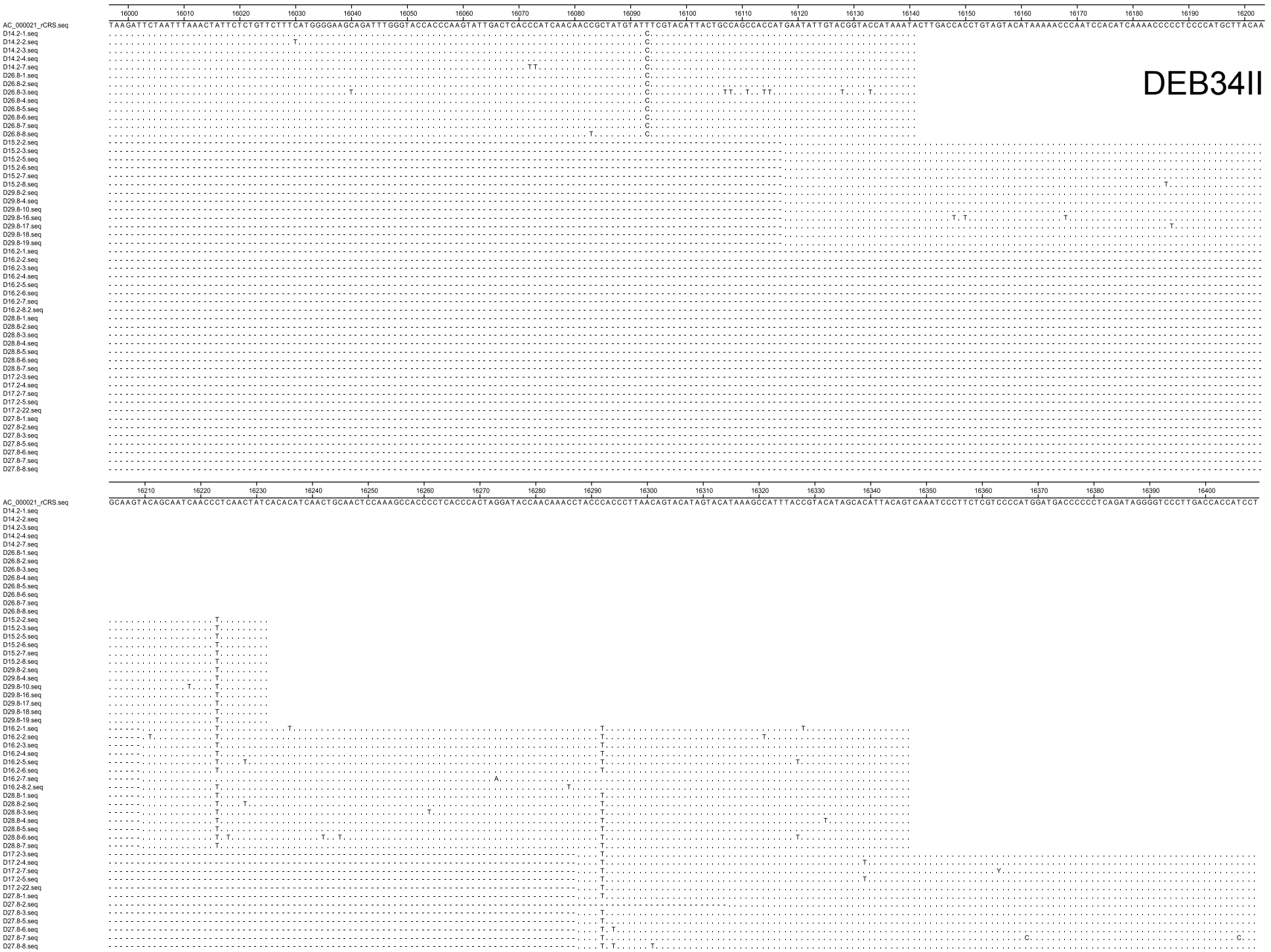
DEB34II
AC_000021_rCRS.seq
D14.2-1.seq
D14.2-2.seq
D14.2-3.seq
D14.2-4.seq
D14.2-7.seq
D26.8-1.seq
D26.8-2.seq
D26.8-3.seq
D26.8-4.seq
D26.8-5.seq
D26.8-6.seq
D26.8-7.seq
D26.8-8.seq
D15.2-2.seq
D15.2-3.seq
D15.2-5.seq
D15.2-6.seq
D15.2-7.seq
D15.2-8.seq
D29.8-2.seq
D29.8-4.seq
D29.8-10.seq
D29.8-16.seq
D29.8-17.seq
D29.8-18.seq
D29.8-19.seq
D16.2-1.seq
D16.2-2.seq
D16.2-3.seq
D16.2-4.seq
D16.2-5.seq
D16.2-6.seq
D16.2-7.seq
D16.2-8.2.seq
D28.8-1.seq
D28.8-2.seq
D28.8-3.seq
D28.8-4.seq
D28.8-5.seq
D28.8-6.seq
D28.8-7.seq
D17.2-3.seq
D17.2-4.seq
D17.2-7.seq
D17.2-5.seq
D17.2-22.seq
D27.8-1.seq
D27.8-2.seq
D27.8-3.seq
D27.8-5.seq
D27.8-6.seq
D27.8-7.seq
D27.8-8.seq

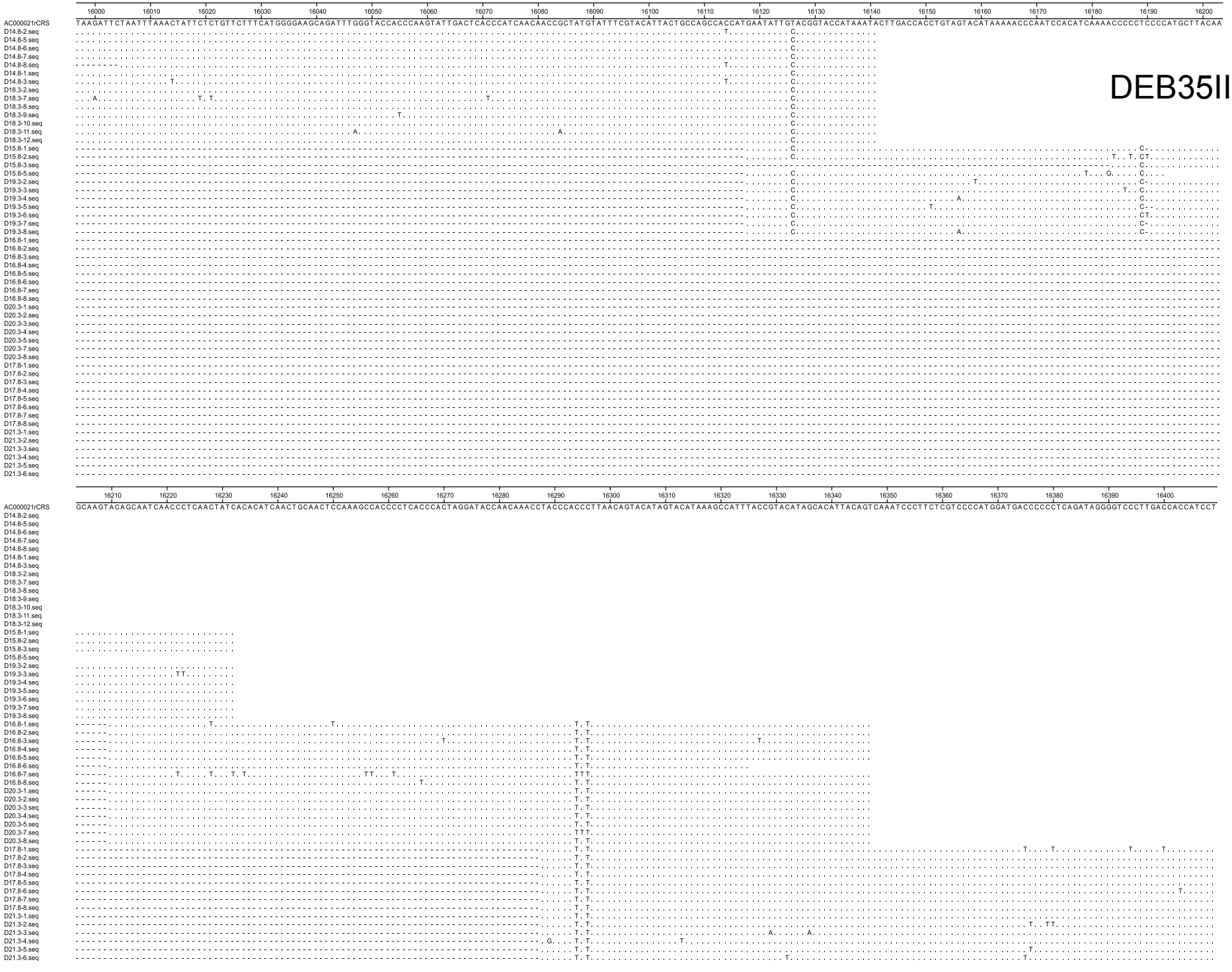
DEB35II

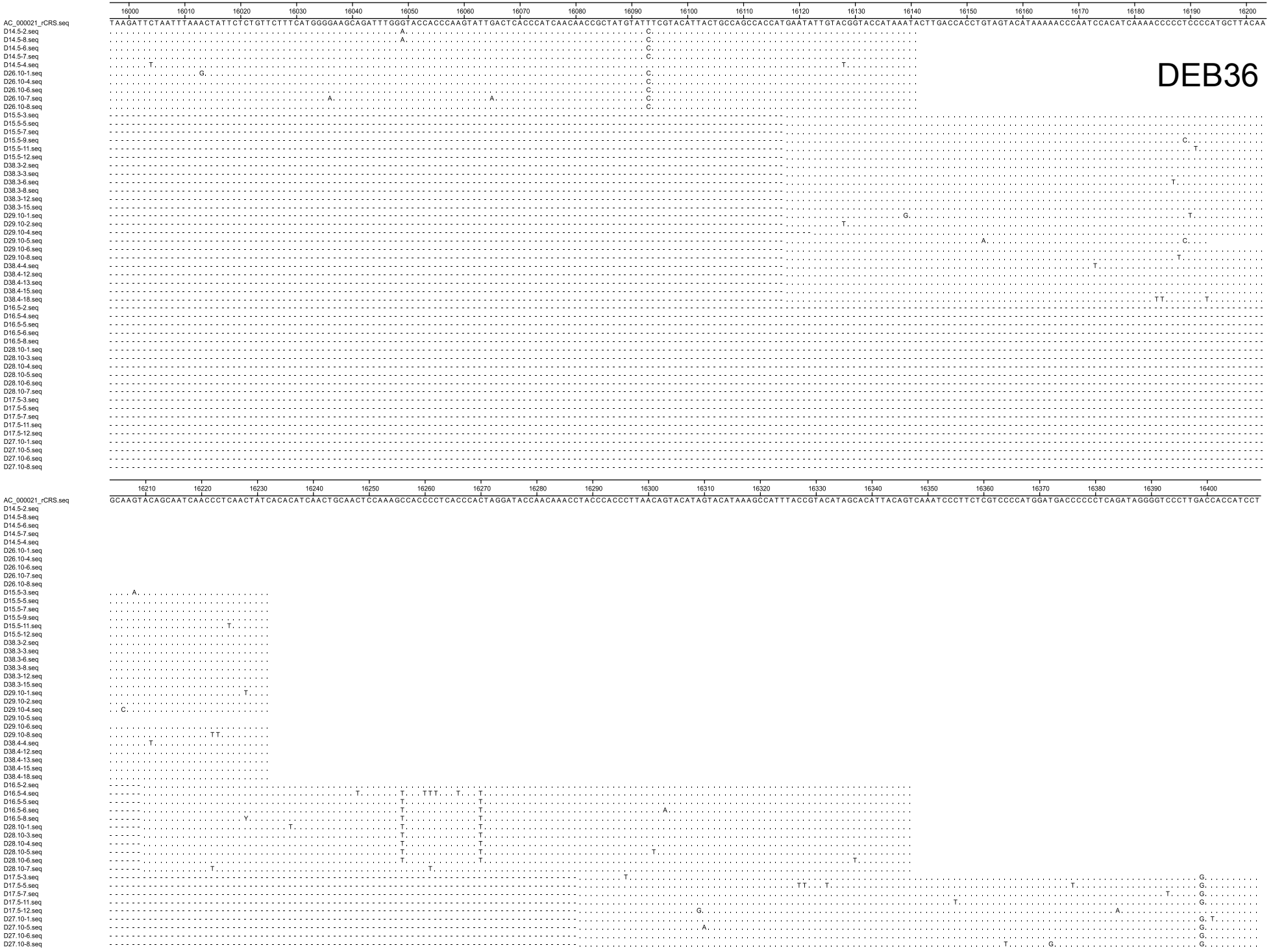
DEB36
16000 16010 16020 16030 16040 16050 16060 16070 16080 16090 16100 16110 16120 16130 16140 16150 16160 16170 16180 16190 16200
AC_000021_rCRS.seq
TAAGATTCTAATTTAAACTATTCTCTGTTCTTTCATGGGGAAGCAGATTTGGGTACCACCCAAGTATTGACTCACCCATCAACAACCGCTATGTATTTCGTACATTACTGCCAGCCACCATGAATATTGTACGGTACCATAAATACTTGACCACCTGTAGTACATAAAAACCCAATCCACATCAAAACCCCCTCCCCATGCTTACAA
D14.5-2.seq
D14.5-8.seq
D14.5-6.seq
D14.5-7.seq
D14.5-4.seq
D26.10-1.seq
D26.10-4.seq
D26.10-6.seq
D26.10-7.seq
D26.10-8.seq
D15.5-3.seq
D15.5-5.seq
D15.5-7.seq
D15.5-9.seq
D15.5-11.seq
D15.5-12.seq
D38.3-2.seq
D38.3-3.seq
D38.3-6.seq
D38.3-8.seq
D38.3-12.seq
D38.3-15.seq
D29.10-1.seq
D29.10-2.seq
D29.10-4.seq
D29.10-5.seq
D29.10-6.seq
D29.10-8.seq
D38.4-4.seq
D38.4-12.seq
D38.4-13.seq
D38.4-15.seq
D38.4-18.seq
D16.5-2.seq
D16.5-4.seq
D16.5-5.seq
D16.5-6.seq
D16.5-8.seq
D28.10-1.seq
D28.10-3.seq
D28.10-4.seq
D28.10-5.seq
D28.10-6.seq
D28.10-7.seq
D17.5-3.seq
D17.5-5.seq
D17.5-7.seq
D17.5-11.seq
D17.5-12.seq
D27.10-1.seq
D27.10-5.seq
D27.10-6.seq
D27.10-8.seq
16210 16220 16230 16240 16250 16260 16270 16280 16290 16300 16310 16320 16330 16340 16350 16360 16370 16380 16390 16400
AC_000021_rCRS.seq
GCAAGTACAGCAATCAACCCTCAACTATCACACATCAACTGCAACTCCAAAGCCACCCCTCACCCACTAGGATACCAACAAACCTACCCACCCTTAACAGTACATAGTACATAAAGCCATTTACCGTACATAGCACATTACAGTCAAATCCCTTCTCGTCCCCATGGATGACCCCCCTCAGATAGGGGTCCCTTGACCACCATCCT
D14.5-2.seq
D14.5-8.seq
D14.5-6.seq
D14.5-7.seq
D14.5-4.seq
D26.10-1.seq
D26.10-4.seq
D26.10-6.seq
D26.10-7.seq
D26.10-8.seq
D15.5-3.seq
D15.5-5.seq
D15.5-7.seq
D15.5-9.seq
D15.5-11.seq
D15.5-12.seq
D38.3-2.seq
D38.3-3.seq
D38.3-6.seq
D38.3-8.seq
D38.3-12.seq
D38.3-15.seq
D29.10-1.seq
D29.10-2.seq
D29.10-4.seq
D29.10-5.seq
D29.10-6.seq
D29.10-8.seq
D38.4-4.seq
D38.4-12.seq
D38.4-13.seq
D38.4-15.seq
D38.4-18.seq
D16.5-2.seq
D16.5-4.seq
D16.5-5.seq
D16.5-6.seq
D16.5-8.seq
D28.10-1.seq
D28.10-3.seq
D28.10-4.seq
D28.10-5.seq
D28.10-6.seq
D28.10-7.seq
D17.5-3.seq
D17.5-5.seq
D17.5-7.seq
D17.5-11.seq
D17.5-12.seq
D27.10-1.seq
D27.10-5.seq
D27.10-6.seq
D27.10-8.seq

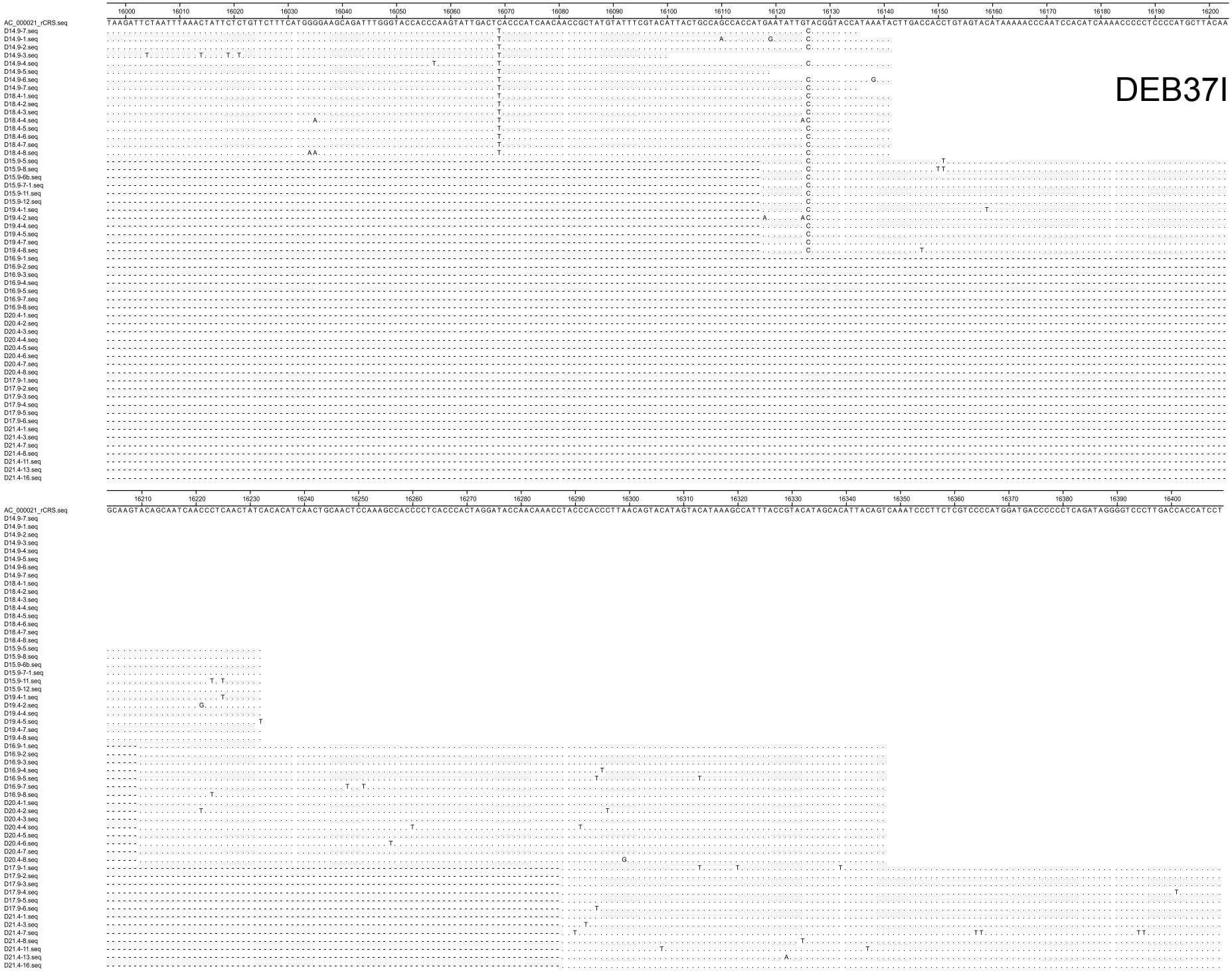
DEB37I
AC_000021_rCRS.seq
D14.9-7.seq
D14.9-1.seq
D14.9-2.seq
D14.9-3.seq
D14.9-4.seq
D14.9-5.seq
D14.9-6.seq
D14.9-7.seq
D18.4-1.seq
D18.4-2.seq
D18.4-3.seq
D18.4-4.seq
D18.4-5.seq
D18.4-6.seq
D18.4-7.seq
D18.4-8.seq
D15.9-5.seq
D15.9-8.seq
D15.9-6b.seq
D15.9-7-1.seq
D15.9-11.seq
D15.9-12.seq
D19.4-1.seq
D19.4-2.seq
D19.4-4.seq
D19.4-5.seq
D19.4-7.seq
D19.4-8.seq
D16.9-1.seq
D16.9-2.seq
D16.9-3.seq
D16.9-4.seq
D16.9-5.seq
D16.9-7.seq
D16.9-8.seq
D20.4-1.seq
D20.4-2.seq
D20.4-3.seq
D20.4-4.seq
D20.4-5.seq
D20.4-6.seq
D20.4-7.seq
D20.4-8.seq
D17.9-1.seq
D17.9-2.seq
D17.9-3.seq
D17.9-4.seq
D17.9-5.seq
D17.9-6.seq
D21.4-1.seq
D21.4-3.seq
D21.4-7.seq
D21.4-8.seq
D21.4-11.seq
D21.4-13.seq
D21.4-16.seq
16000
16010
16020
16030
16040
16050
16060
16070
16080
16090
16100
16110
16120
16130
16140
16150
16160
16170
16180
16190
16200
TAAGATTCTAATTTAAACTATTCTCTGTTCTTTCATGGGGAAGCAGATTTGGGTACCACCCAAGTATTGACTCACCCATCAACAACCGCTATGTATTTCGTACATTACTGCCAGCCACCATGAATATTGTACGGTACCATAAATACTTGACCACCTGTAGTACATAAAAACCCAATCCACATCAAAACCCCCTCCCCATGCTTACAA
16210
16220
16230
16240
16250
16260
16270
16280
16290
16300
16310
16320
16330
16340
16350
16360
16370
16380
16390
16400
GCAAGTACAGCAATCAACCCTCAACTATCACACATCAACTGCAACTCCAAAGCCACCCCTCACCCACTAGGATACCAACAAACCTACCCACCCTTAACAGTACATAGTACATAAAGCCATTTACCGTACATAGCACATTACAGTCAAATCCCTTCTCGTCCCCATGGATGACCCCCCTCAGATAGGGGTCCCTTGACCACCATCCT

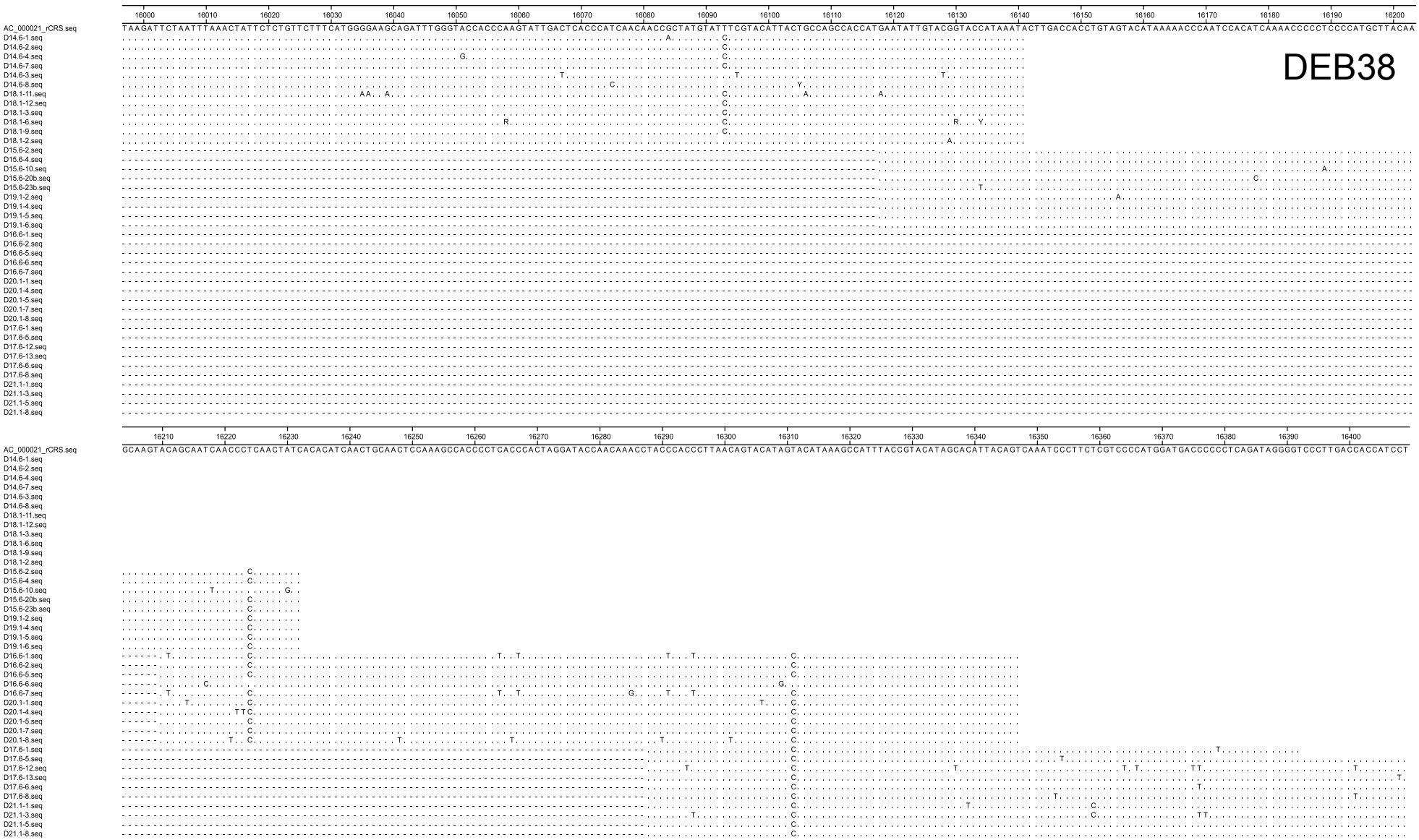
DEB38
16000 16010 16020 16030 16040 16050 16060 16070 16080 16090 16100 16110 16120 16130 16140 16150 16160 16170 16180 16190 16200
AC_000021_rCRS.seq
D14.6-1.seq
D14.6-2.seq
D14.6-4.seq
D14.6-7.seq
D14.6-3.seq
D14.6-8.seq
D18.1-11.seq
D18.1-12.seq
D18.1-3.seq
D18.1-6.seq
D18.1-9.seq
D18.1-2.seq
D15.6-2.seq
D15.6-4.seq
D15.6-10.seq
D15.6-20b.seq
D15.6-23b.seq
D19.1-2.seq
D19.1-4.seq
D19.1-5.seq
D19.1-6.seq
D16.6-1.seq
D16.6-2.seq
D16.6-5.seq
D16.6-6.seq
D16.6-7.seq
D20.1-1.seq
D20.1-4.seq
D20.1-5.seq
D20.1-7.seq
D20.1-8.seq
D17.6-1.seq
D17.6-5.seq
D17.6-12.seq
D17.6-13.seq
D17.6-6.seq
D17.6-8.seq
D21.1-1.seq
D21.1-3.seq
D21.1-5.seq
D21.1-8.seq
16210 16220 16230 16240 16250 16260 16270 16280 16290 16300 16310 16320 16330 16340 16350 16360 16370 16380 16390 16400
AC_000021_rCRS.seq
D14.6-1.seq
D14.6-2.seq
D14.6-4.seq
D14.6-7.seq
D14.6-3.seq
D14.6-8.seq
D18.1-11.seq
D18.1-12.seq
D18.1-3.seq
D18.1-6.seq
D18.1-9.seq
D18.1-2.seq
D15.6-2.seq
D15.6-4.seq
D15.6-10.seq
D15.6-20b.seq
D15.6-23b.seq
D19.1-2.seq
D19.1-4.seq
D19.1-5.seq
D19.1-6.seq
D16.6-1.seq
D16.6-2.seq
D16.6-5.seq
D16.6-6.seq
D16.6-7.seq
D20.1-1.seq
D20.1-4.seq
D20.1-5.seq
D20.1-7.seq
D20.1-8.seq
D17.6-1.seq
D17.6-5.seq
D17.6-12.seq
D17.6-13.seq
D17.6-6.seq
D17.6-8.seq
D21.1-1.seq
D21.1-3.seq
D21.1-5.seq
D21.1-8.seq

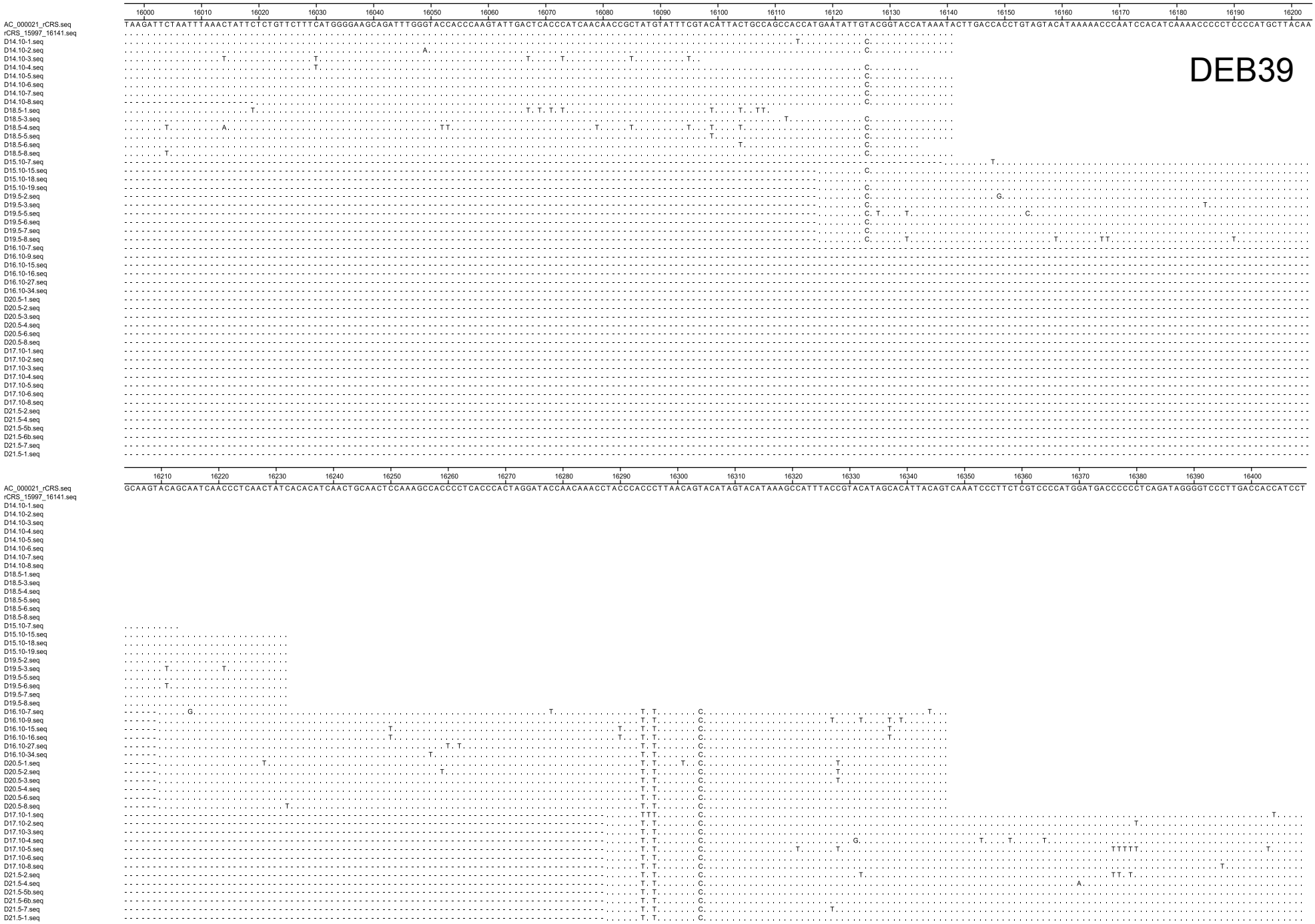
DEB39
