# Supplementary figures and images for: Ancient DNA from European Early Neolithic Farmers Reveals Their Near Eastern Affinities
Source: PLoS Biol. 2010 Nov 9;8(11):e1000536. doi: 10.1371/journal.pbio.1000536 (PMC2976717; doi:10.1371/journal.pbio.1000536)

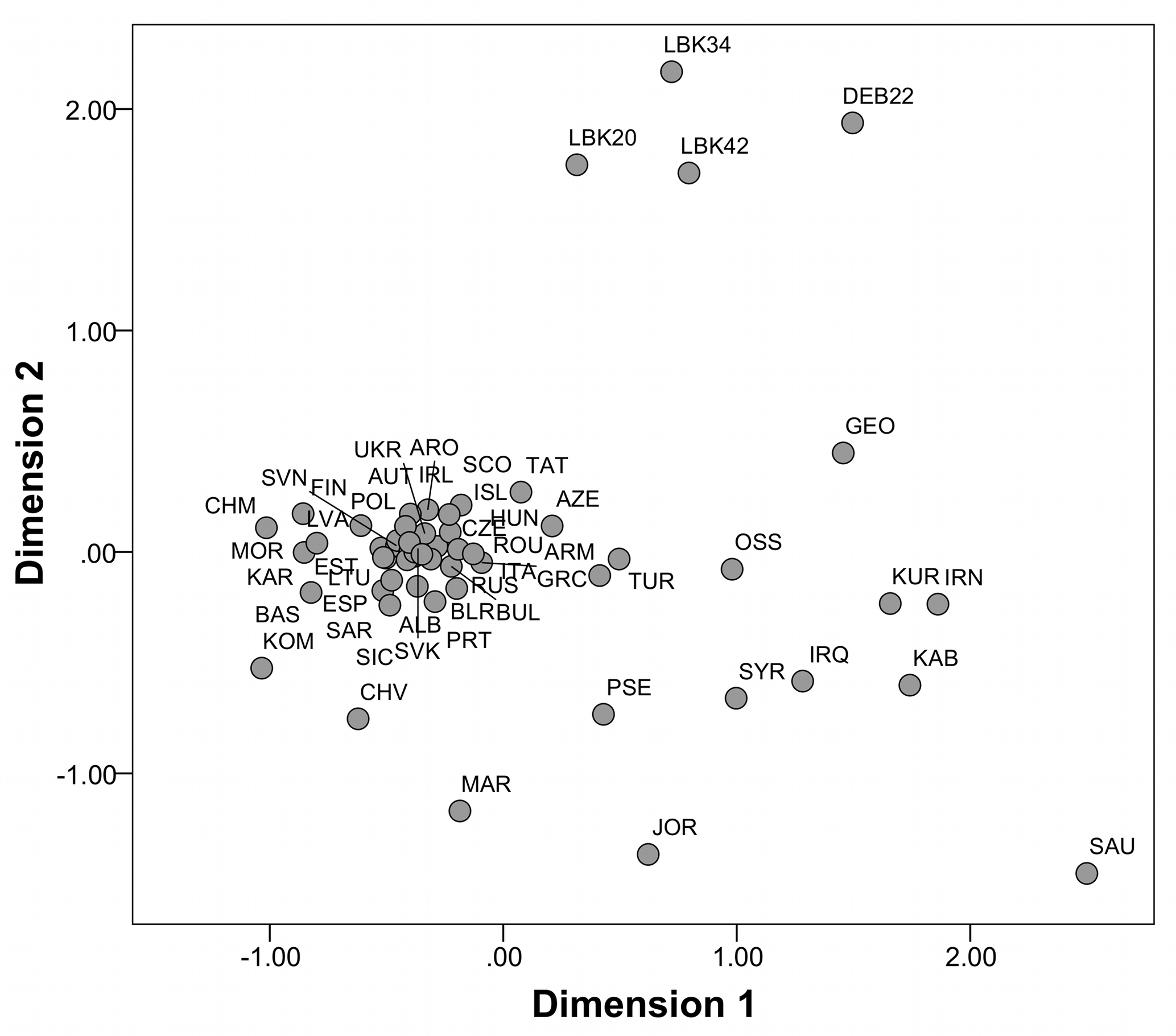

Supplement: Figure S1 — Multidimensional scaling plot of genetic distances based on haplogroup frequencies (alienation = 0, 1117760; stress = 0, 1053030). Population abbreviations are consistent with Figure 1, and further population details and references are listed in Table S6. (1.05 MB TIF) [file pbio.1000536.s002.tif]

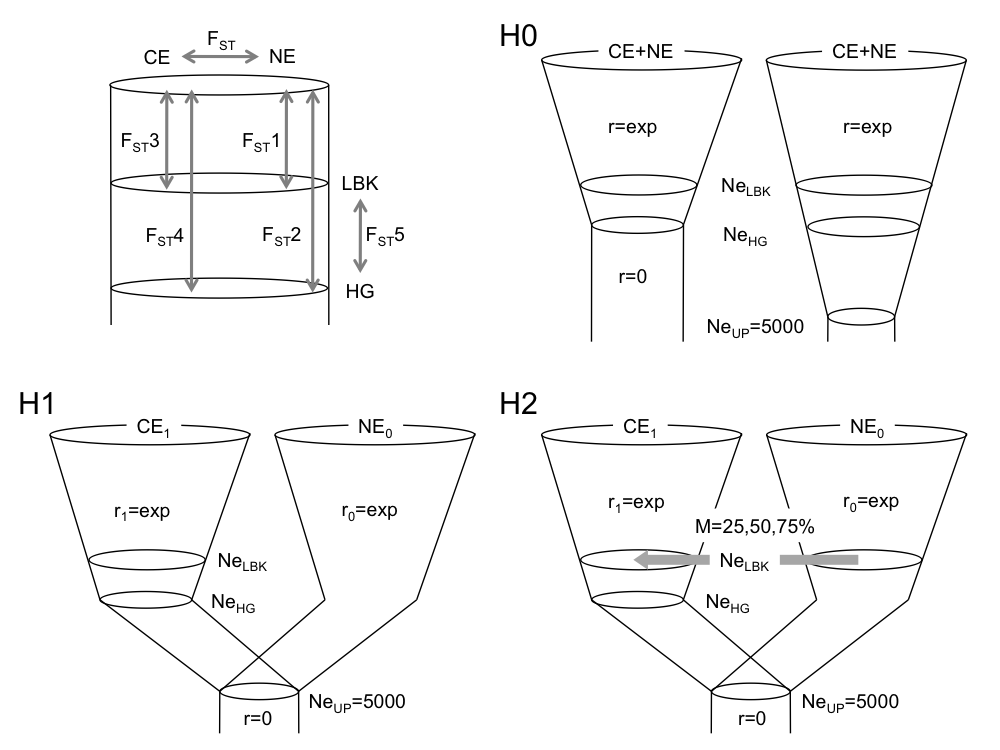

Supplement: Figure S2 — Demographic models and population pairwise F ST values used in BayeSSC analyses. CE1, Central European deme 1; exp, exponential; HG, hunter–gatherers; M, migrants; Ne, effective population size; NE0, Near Eastern deme 0; r, growth rate; UP, Upper Paleolithic. (3.00 MB TIF) [file pbio.1000536.s003.tif]

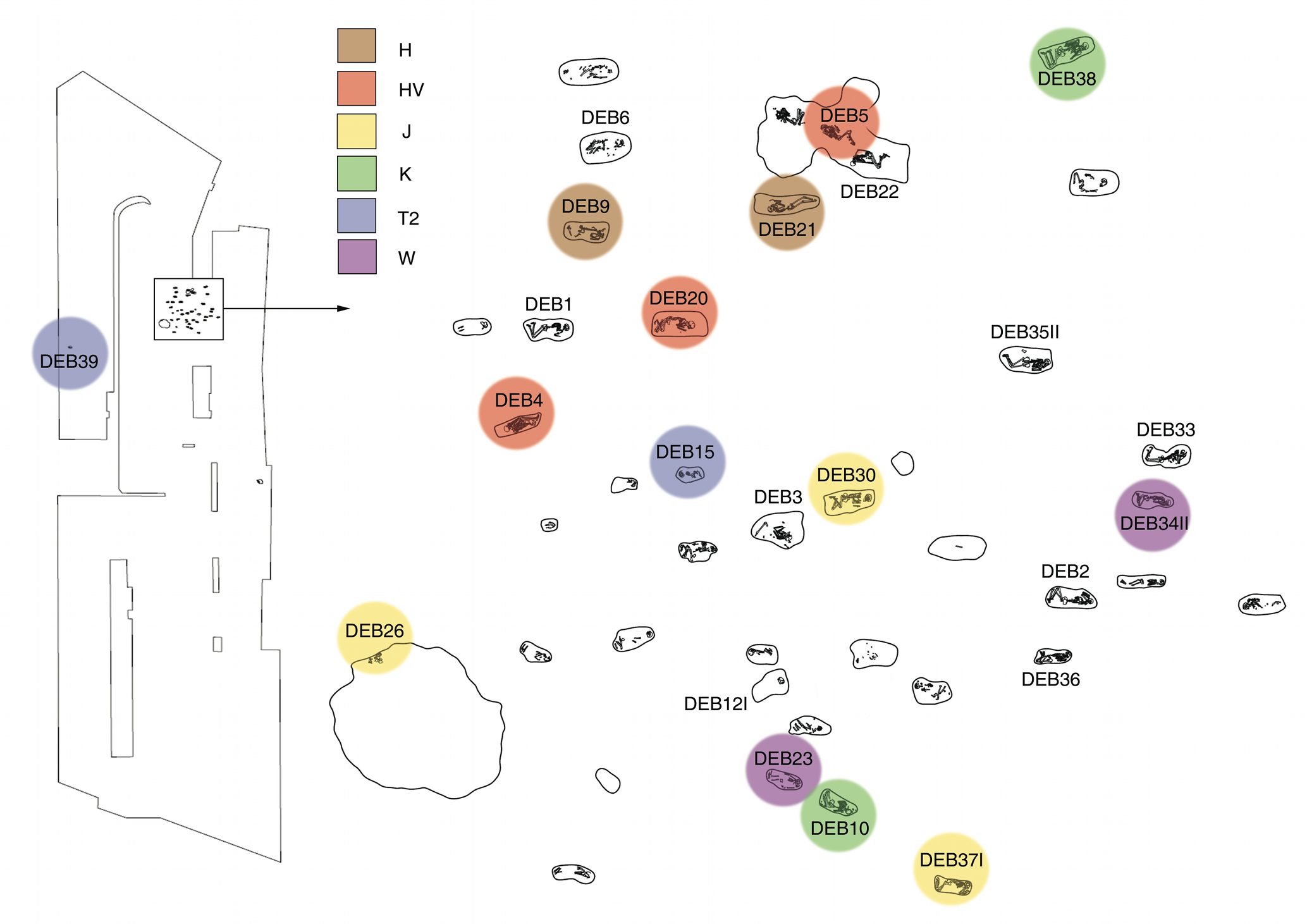

Supplement: Figure S3 — Map of the Neolithic graveyard Derenburg Meerenstieg II. (1.29 MB TIF) [file pbio.1000536.s004.tif]
