# Supplementary material for: Ancient DNA from European Early Neolithic Farmers Reveals Their Near Eastern Affinities
Source: PLoS Biol. 2010 Nov 9;8(11):e1000536. doi: 10.1371/journal.pbio.1000536 (PMC2976717; doi:10.1371/journal.pbio.1000536)
Supplement: Table S1 — Results of mtDNA coding region SNP typing using the GenoCoRe22 assay. SNPs are detected in forward orientation (L-strand) unless stated otherwise (underlined), and SNP results are reported as typed in the SBE assay. Italicized samples were discarded from further analyses. Samples were typed twice from two independent extracts except for individuals deb1 and deb2. Empty cells indicate either allelic dropout or a relative fluorescence unit value below the threshold of 50. SNP 3594_L3'4 consistently yielded relative fluorescence unit values below 50, and was not reported. Subsequent primer mixes were adjusted for the suboptimal performance of SNP3594 (Table S7). (0.26 MB DOC) [file pbio.1000536.s005.doc]

**Table S1. Results of mtDNA coding region SNP typing using the GenoCore22 assay.** SNPs are detected in forward orientation (L-strand) unless stated otherwise (underlined) and SNP results are reported as typed in the SBE assay. Italicized samples were discarded from further analyses. Samples had been typed twice from two independent extracts except for DEB1 and DEB2. Empty cells indicate either allelic dropout or a relative fluororescent unit (rfu) value below the threshold of 50. SNP 3594_L3’4 yielded consistently rfu’s below 50, and were not reported. Subsequent primermixes were adjusted for the suboptimal performance of SNP3594 (Table S7).

| **Hg** | **Sample** | **Ex#** | **13928_R9** | **3594_L3’4** | **10550_K** | **11467_U** | **4248_A** | **8994_W** | **13263_C** | **13368_T** | **11719_ R0** | **8280delB** | **4580_V,M3** | **6371_X** | **10238_N1** | **7028_H** | **10034_I** | **5178_D** | **14766_HV** | **12705_R** | **10873_N** | **12612_J** | **2758_L2’6** | **10400_M** |
| --- | --- | --- | --- | --- | --- | --- | --- | --- | --- | --- | --- | --- | --- | --- | --- | --- | --- | --- | --- | --- | --- | --- | --- | --- |
|  | **Ancestral state** | | **C** | **G** | **T** | **A** | **T** | **G** | **T** | **G** | **T** | **A** | **C** | **G** | **T** | **A** | **T** | **G** | **A** | **A** | **G** | **A** | **A** | **A** |
| N1 | DEB1 | DB8 | C |  | T | A | T | G | T | G | T | A | C | G | C | A | T | G | A | A | A | A | G | G |
| K | DEB10 | DA6 | C |  | C | G | T | G | T | G | T | A | C | G | T | g/a | T | G | A | G | A | A | G | G |
| K | DEB10 | DD6 | C |  | C | G | T | G | T | G | T | A | C | G | T | A | T | G | A | G | A | A | G | G |
| T | DEB11 | DA5 | C |  | T | A | T | G | T | A | T | A | C | G | T | A | T | G |  | G | A | A | G | G |
| T? | DEB11 | DD5 | C |  | T | A | T | G | T | g/a | T | A | C | G | T | A | T | G |  | G | A | A | G | g/a |
| V | DEB12I | DB1 | C |  | T | A | T | G | T | G | c/t | A | T | G | T | A | T | G | G | G | A | A | G | G |
| V | DEB12I | DD8 | C |  | T | A | T | G | T | G | C | A | T | G | T | A | T | G | G | G | A | A | G | G |
|  | *DEB13* | *DA4* |  |  | *T* | *A* | *T* |  |  | *G* | *T* |  | *C* | *G* |  | *G* | *T* |  |  |  |  | *G* |  | *G* |
|  | *DEB13* | *DD4* |  |  | *T* |  |  | *G* | *T* | *G* |  |  |  | *G* | *T* | *g/a* | *T* | *G* |  |  |  |  |  | *G* |
| *N?* | *DEB14II* | *DB2* | *C* |  | *T* | *A* | *T* | *G* | *T* | *G* | *T* | *A* | *C* | *G* | *T* | *A* | *T* | *G* |  | *A* | *A* | *A* | *G* | *G* |
|  | *DEB14II* | *DD9* | *C* |  | *T* | *A* | *T* | *g/a* | | *G* | *T* | *A* | *c/t* | *G* | *T* | *A* | *T* | *G* |  | *A* | *g/a* | *G* | *g/a* | *G* |
| T | DEB15 | DB3 | C |  | T | A | T | G | T | A | T | A | C | G | T | A | T | G | A | G | A | A | G | G |
| T | DEB15 | DD10 | C |  | T | A | T | G | T | A | T | A | C | G | T | A | T | G | A | G | A | A | G | G |
|  | *DEB18* | *DB4* | *C* |  | *T* | *A* | *T* | *G* | *T* | *G* | *T* | *A* | *C* | *G* | *T* | *g/a* | *T* | *G* | *A* | *g/a* | *G* | *A* | *G* | *g/a* |
|  | *DEB18* | *DD11* | *C* |  |  |  |  |  |  | *G* |  | *G* |  | *G* |  | *G* |  | *G* |  |  | *G* | *G* |  |  |
| K | DEB2 | DC4 | C |  | C | G | T | G | T | G | T | A | C | G | T | A | T | G | A | g/a | A | G | G | G |
| HV | DEB20 | DB6 | C |  | T | A | T | G | T | G | C | A | C | G | T | A | T | G | G | G | A | A | G | G |
| HV | DEB20 | DE1 | C |  | T | A | T | G | T | G | C | A | C | G | T | A | T | G | G | G | A | A | G | G |
| H | DEB21 | DB7 | C |  | T | A | T | G | T | G | C | A | C | G | T | G | T | G | G | G | A | A | G | G |
| H | DEB21 | DE2 | C |  | T | A | T | G | T | G | C | A | C | G | T | G | T | G | G | G | A | A | G | G |
| N1 | DEB22 | DB5 | C |  | T | A | T | G | T | G | T | A | C | G | C | A | T | G | A | A | A | A | G | G |
| N1 | DEB22 | DD12 | C |  | T | A | T | G | T | G | T | A | C | G | C | A | T | G | A | A | A | A | G | G |
| **Hg** | **Sample** | **Ex#** | **13928_R9** | **3594_L2** | **10550_K** | **11467_U** | **4248_A** | **8994_W** | **13263_C** | **13368_T** | **11719_ R0** | **8280delB** | **4580_V,M3** | **6371_X** | **10238_N1** | **7028_H** | **10034_I** | **5178_D** | **14766_HV** | **12705_R** | **10873_N** | **12612_J** | **2758_L0'1** | **10400_M** |
|  | **Ancestral state** | | **C** | **G** | **T** | **A** | **T** | **G** | **T** | **G** | **T** | **A** | **C** | **G** | **T** | **A** | **T** | **G** | **A** | **A** | **G** | **A** | **A** | **A** |
| W | DEB23 | DA7 | C |  | T | A | T | A | T | G | T | A | C | G | T | A | T | G | A | A | A | A | G | G |
| W | DEB23 | DD7 | C |  | T | A | T | A | T | G | T | A | C | G | T | A | T | G | A | A | A | A | G | G |
|  | *DEB25* | *DB9* |  |  | *T* | *A* | *T* | *A* |  | *G* |  | *G* | *C* | *G* | *T* | *g/a* | *T* | *G* |  |  |  | *A* |  |  |
|  | *DEB25* | *DE3* | *C* |  | *C* | *g/a* | *T* | *G* | *T* | *G* | *T* | *G* | *C* | *G* | *T* | *G* | *T* | *G* | *g/a* | | *A* | *A* | *G* |  |
| J | DEB26 | DB10 | C |  | T | A | T | G | T | G | T | A | C | G | T | A | T | G | A | G | A | G | G | G |
| J | DEB26 | DE4 | C |  | T | A | T | G | T | G | T | A | C | G | T | A | T | G | A | G | A | G | G | G |
|  | *DEB29II* | *DC1* |  |  |  |  |  |  |  |  |  | *G* |  | *G* |  |  |  | *G* |  |  |  |  |  |  |
| *K* | *DEB29II* | *DE7* | *C* |  | *C* | *G* | *T* | *G* | *T* | *G* | *T* | *A* | *C* | *G* | *T* | *A* | *T* | *G* | *A* | *G* | *A* | *g/a* | *G* | *G* |
| J | DEB30 | DB12 | C |  | T | A | T | G | T | G | T | A | C | G | T | A | T | G | A | G | A | G | G | G |
| J | DEB30 | DE6 | C |  | T | A | T | G | T | G | T | A | C | G | T | A | T | G | A | G | A | G | G | G |
| T | DEB32 | DB11 | C |  | T | A | T | G | T | A | T | A | C | G | T | A | T | G | A | G | A | A | G | G |
| T | DEB32 | DE5 | C |  | T | A | T | G |  | A | T | G | C | G | T | A | T | G |  | G | A | A | G | G |
| T | DEB33 | DC3 | C |  | T | A | T | G | T | A | T | A | C | G | T | A | T | G | A | G | A | A | G | G |
| T | DEB33 | DE9 | C |  | T | A | T | G | T | A | T | A | C | G | T | A | T | G | A | G | A | A | G | G |
| W | DEB34II | DC2 | C |  | T | A | T | A | T | G | T | A | C | G | T | A | T | G | A | A | A | A | G | G |
| W | DEB34II | DE8 | C |  | T | A | T | A | T | G | T | A | C | G | T | A | T | G | A | A | A | A | G | G |
|  | *DEB35* | *DC7* | *C* |  | *T* | *A* |  |  | *T* |  | *T* |  | *C* | *G* |  | *G* |  | *G* |  |  | *A* | *A* |  | *A* |
|  | *DEB35* | *DF2* | *C* |  | *T* |  | *T* | *G* |  | *G* | *T* | *A* | *C* | *G* | *T* |  | *T* | *G* |  | *G* |  | *N* | *G* |  |
| T | DEB35II | DC8 | C |  | T | A | T | G | T | A | T | A | C | G | T | A | T | G | A | G | A | A | G | G |
| T | DEB35II | DF3 | C |  | T | A | T | G | T | A | T | A | C | G | T | A | T | G | A | G | A | A | G | G |
| U | DEB36 | DC5 | C |  | T | G | T | G | T | G | T | A | C | G | T | A | T | G | A | G | A | A | G | G |
| U | DEB36 | DE10 | C |  | T | G | T | G | T | G | T | A | C | G | T | A | T | G | A | G | A | A | G | G |
| J | DEB37 | DC9 | C |  | T | A | T | G | T | G | T | A | C | G | T | A | T | G | A | G | A | G | G | G |
| J | DEB37 | DF4 | C |  | T | A | T | G | T | G | T | A | C | G | T | A | T | G | A | G | A | G | G | G |
| **Hg** | **Sample** | **Ex#** | **13928_R9** | **3594_L2** | **10550_K** | **11467_U** | **4248_A** | **8994_W** | **13263_C** | **13368_T** | **11719_ R0** | **8280delB** | **4580_V,M3** | **6371_X** | **10238_N1** | **7028_H** | **10034_I** | **5178_D** | **14766_HV** | **12705_R** | **10873_N** | **12612_J** | **2758_L0'1** | **10400_M** |
|  | **Ancestral state** | | **C** | **G** | **T** | **A** | **T** | **G** | **T** | **G** | **T** | **A** | **C** | **G** | **T** | **A** | **T** | **G** | **A** | **A** | **G** | **A** | **A** | **A** |
| K | DEB38 | DC6 | C |  | C | G | T | G | T | G | T | A | C | G | T | A | T | G | A | G | A | A | G | G |
| K | DEB38 | DF1 | C |  | C | G | T | G | T | G | T | A | C | G | T | A | T | G | A | G | A | A | G | G |
| T | DEB39 | DC10 | C |  | T | A | T | G | T | A | T | A | C | G | T | A | T | G | A | G | A | A | G | G |
| T | DEB39 | DF5 | C |  | T | A | T | G | T | A | T | A | C | G | T | A | T | G | A | G | A | A | G | G |
| *H* | *DEB6* | *DA2* | *C* |  | *T* | *A* | *T* | *G* | *T* | *G* | *C* | *A* | *C* | *G* | *T* | *G* | *T* | *G* | *G* | *G* | *A* | *A* | *G* | *G* |
| *H?* | *DEB6* | *DD2* | *C* |  | *T* | *A* | *T* | *G* | *T* | *G* | *C* |  | *C* | *G* | *T* | *g/a* | *T* | *G* |  | *G* | *A* | *A* | *G* | *G* |
|  | *DEB8* | *DA3* | *C* |  | *c/t* |  | *T* |  |  | *G* | *T* | *A* |  | *G* | *T* | *A* | *T* | *G* |  | *G* |  | *g/a* | *G* |  |
|  | *DEB8* | *DD3* | *C* |  | *T* |  |  | *A* | *T* | *G* | *T* | *g/a* | *C* | *G* | *T* | *g/a* | *T* | *G* |  | *G* | *G* | *A* |  | *G* |
| H | DEB9 | DA1 | C |  | T | A | T | G | T | G | C | A | C | G | T | G | T | G | G | G | A | A | G | G |
| H | DEB9 | DD1 | C |  | T | A | T | G | T | G | C | A | C | G | T | G | T | G | G | G | A | A | G | G |
