# Supplementary material for: Ancient DNA from European Early Neolithic Farmers Reveals Their Near Eastern Affinities
Source: PLoS Biol. 2010 Nov 9;8(11):e1000536. doi: 10.1371/journal.pbio.1000536 (PMC2976717; doi:10.1371/journal.pbio.1000536)
Supplement: Table S2 — Results of Y chromosome SNP typing using the GenoY25 assay. SNPs are detected in forward orientation unless stated otherwise (underlined), and SNP results are reported as typed in the SBE assay. (0.21 MB DOC) [file pbio.1000536.s006.doc]

**Table S2. Results of Y-chromosome SNP typing using the GenoY25 assay.** SNPs are detected in forward orientation unless stated otherwise (underlined) and SNP results are reported as typed in the SBE assay.

| **YCC2008 Hg** | | **D** | **N** | **Q** | **CR** | **R1b1b1a** | **P** | **BR/R1a** | **E1b1a** | **F** | **J1** | **R1b** | **J** | **J2** | **O** | **E1b1b1** | **I** | **G** | **R1b1b** | **O3** | **R1a1** | **E** | **E1b1b1a** | **K** | **R** | **C** | **G1** | **G2** | **G2a3** | **H** |
| --- | --- | --- | --- | --- | --- | --- | --- | --- | --- | --- | --- | --- | --- | --- | --- | --- | --- | --- | --- | --- | --- | --- | --- | --- | --- | --- | --- | --- | --- | --- |
| **#** | **sample/SNP** | **M174** | **M231** | **M242** | **M168** | **S21** | **M45** | **SRY10831** | **M2** | **M89** | **M267** | **M343** | **M304** | **M172** | **M175** | **M35** | **M170** | **M201** | **M269** | **M122** | **M17** | **M96** | **M78** | **M9** | **M207** | **M216** | **M285** | **P287** | **S126 (L30)** | **M69** |
| **ancestral state** | | **T** | **G** | **C** | **G** | **C** | **G** | **T** | **A** | **C** | **A** | **G** | **A** | **T** | **T** | **G** | **A** | **G** | **A** | **T** | **C** | **G** | **C** | **G** | **T** | **C** | **G** | **G** | **C** | **T** |
| a | DEB12I |  | G |  |  |  |  |  |  |  | A |  |  |  |  |  |  |  |  |  |  |  |  |  |  |  |  |  |  |  |
| b | DEB12I |  |  |  |  |  |  |  |  |  |  |  |  |  |  |  |  |  |  |  |  |  |  |  |  |  |  |  |  |  |
| **DEB12I consensus** | |  |  |  |  |  |  |  |  |  |  |  |  |  |  |  |  |  |  |  |  |  |  |  |  |  |  |  |  |  |
| a | DEB20 | T | A |  |  | C | G |  | A |  | A |  |  |  |  | G |  |  |  |  |  |  |  |  |  |  |  |  |  |  |
| a | DEB20 | T | G | C |  | C | G |  |  | T | A | G |  |  |  |  |  |  |  |  | C |  |  |  |  |  |  |  |  |  |
| a | DEB20 | T | G | C |  | C | G |  | A | T |  |  |  |  | T |  |  | G |  |  |  |  |  |  |  |  |  |  |  | T |
| b | DEB20 | T | G | C | A | C | G | C | A | T | A | G | A | T | T |  |  | G |  | T | C | G |  | G | T | C |  |  |  |  |
| b | DEB20 | T | G | C | A | C | G | C | A | T | A | G | A | T | T |  | A | G | A | T | C | G | C | G | T | C |  |  |  |  |
| b | DEB20 | T | G | C | A | C | G | C | A | T | A | G | A | T | T | G | A | G |  | T | C | G | C | G | T | C |  |  |  | T |
| **DEB20 consensus F*** | | **T** | **G** | **C** | **A** | **C** | **G** | **C** | **A** | **T** | **A** | **G** | **A** | **T** | **T** | **G** | **A** | **G** | **a** | **T** | **C** | **G** | **C** | **G** | **T** | **C** |  |  |  | **T** |
| a | DEB22 |  |  |  |  |  |  |  |  |  |  |  |  |  |  |  |  |  |  |  |  |  |  |  |  |  |  |  |  |  |
| b | DEB22 |  |  |  |  |  |  |  |  |  |  |  |  |  |  |  |  |  |  |  |  |  |  |  |  |  |  |  |  |  |
| **DEB22 consensus** | |  |  |  |  |  |  |  |  |  |  |  |  |  |  |  |  |  |  |  |  |  |  |  |  |  |  |  |  |  |
| a | DEB33 |  |  |  |  | C | G |  |  |  |  |  |  |  |  |  |  |  |  |  |  |  |  |  |  |  |  |  |  |  |
| b | DEB33 |  | G |  |  |  |  |  |  |  |  |  |  |  |  |  |  |  |  |  |  |  |  |  |  |  |  |  |  |  |
| **DEB33 consensus** | |  |  |  |  |  |  |  |  |  |  |  |  |  |  |  |  |  |  |  |  |  |  |  |  |  |  |  |  |  |
| **YCC2008 Hg** | | **D** | **N** | **Q** | **CR** | **R1b1b1a** | **P** | **BR/R1a** | **E1b1a** | **F** | **J1** | **R1b** | **J** | **J2** | **O** | **E1b1b1** | **I** | **G** | **R1b1b** | **O3** | **R1a1** | **E** | **E1b1b1a** | **K** | **R** | **C** | **G1** | **G2** | **G2a3** | **H** |
| **#** | **sample/SNP** | **M174** | **M231** | **M242** | **M168** | **S21** | **M45** | **SRY10831** | **M2** | **M89** | **M267** | **M343** | **M304** | **M172** | **M175** | **M35** | **M170** | **M201** | **M269** | **M122** | **M17** | **M96** | **M78** | **M9** | **M207** | **M216** | **M285** | **P287** | **S126 (L30)** | **M69** |
| **ancestral state** | | **T** | **G** | **C** | **G** | **C** | **G** | **T** | **A** | **C** | **A** | **G** | **A** | **T** | **T** | **G** | **A** | **G** | **A** | **T** | **C** | **G** | **C** | **G** | **T** | **C** | **G** | **G** | **C** | **T** |
| a | DEB34II |  |  |  |  | C | G |  | A | T |  | G |  | G | T |  |  | T |  |  | C | G |  |  | T |  |  |  |  |  |
| a | DEB34II | T |  | C |  | C | G |  |  |  |  |  |  |  |  |  |  |  |  |  |  | G |  |  |  |  |  |  |  |  |
| a | DEB34II | T | G | C |  | C | G |  |  | T | A | G |  |  | T |  |  | T |  |  |  |  |  |  |  |  |  |  |  |  |
| a | DEB34II | T | G | c/t | A | C | G |  |  | T | A | G |  |  | T |  |  | T |  |  | C |  |  |  |  |  | G | T | T |  |
| b | DEB34II | T | G | C | A | C | G | C | A | T | A | G |  | T | T |  |  | T |  |  | C | G |  | G | T | C |  |  |  |  |
| b | DEB34II | T | G | C | A | C | G | C | A | T | A | G | A | T | T |  | A | T | A | T | C | G | C | G | T | C |  |  |  |  |
| b | DEB34II | T | G | C | A | C | G | C | A | T | A | G |  | T | T | G | A | T | A | T | C | G | C | G | T | C | G | T | T |  |
| **DEB34II consensus G2a3** | | **T** | **G** | **C** | **A** | **C** | **G** | **C** | **A** | **T** | **A** | **G** | **t** | **T** | **T** | **G** | **A** | **T** | **A** | **T** | **C** | **G** | **C** | **G** | **T** | **C** | **G** | **T** | **T** |  |
| a | DEB38 |  |  |  |  |  |  |  |  |  |  |  |  |  |  |  |  |  |  |  |  |  |  |  |  |  |  |  |  |  |
| a | DEB38 | T | g/a | c/t | A | C | G | C |  | T | A | G |  |  | T |  |  |  |  | T |  |  |  |  |  |  |  |  |  |  |
| a | DEB38 | T | G | C | A | C | G |  |  | T |  | G |  |  | T |  | A | G |  |  |  |  |  |  |  |  |  |  |  | T |
| b | DEB38 | T | G | C | A | C | G | C | A | T | A | G |  | T | T | G |  | G |  | T | C | G |  |  |  | C |  |  |  |  |
| b | DEB38 | T | G | C | A | C | G | C |  | T | A | G | A |  | T |  | A | G | A | T | C | G | C | G | T | C |  |  |  |  |
| b | DEB38 | T | G | C | A | C | G | C | A | T | A | G | A | t | T |  | A | G | A | T | C | G | C | G | T | C |  |  |  | T |
| **DEB38 consensus F*** | | **T** | **G** | **C** | **A** | **C** | **G** | **C** | **A** | **T** | **A** | **G** | **A** | **t** | **T** | **g** | **A** | **G** | **A** | **T** | **C** | **G** | **C** | **G** | **T** | **C** |  |  |  | **T** |
| a | DEB39 |  | A |  |  |  |  |  |  |  |  |  |  |  |  |  |  |  |  |  |  |  |  |  |  |  |  |  |  |  |
| b | DEB39 | T |  |  |  |  |  |  |  |  |  |  |  |  |  |  |  |  |  |  |  |  |  |  |  |  |  |  |  |  |
| **DEB39 consensus** | |  |  |  |  |  |  |  |  |  |  |  |  |  |  |  |  |  |  |  |  |  |  |  |  |  |  |  |  |  |
