# Supplementary material for: Ancient DNA from European Early Neolithic Farmers Reveals Their Near Eastern Affinities
Source: PLoS Biol. 2010 Nov 9;8(11):e1000536. doi: 10.1371/journal.pbio.1000536 (PMC2976717; doi:10.1371/journal.pbio.1000536)
Supplement: Table S5 — Ancient samples from other LBK sites used for population genetics analyses [19]. (0.07 MB PDF) [file pbio.1000536.s009.pdf]

Table S5. Ancient samples from other LBK sites used for population genetics analyses [1-3].

Sample	Site	Country	HVS I Sequence (np 15997-16409), minus 16000	HVS I	RFLP
ASP2	Asparn Schletz 2	Austria	rCRS	H	H
EIL1	Eilsleben	Germany	rCRS	H	H
FLO1	Flomborn	Germany	147a, 223T, 248T, 320T, 355T	N1a	n.d.
FLO2	Flomborn	Germany	093C	H	H
FLO3	Flomborn	Germany	311C	H	H
FLO4	Flomborn	Germany	126C, 294T, 304C	T2	n.d.
FLO5	Flomborn	Germany	224C, 311C	K	n.d.
FLO6	Flomborn	Germany	224C, 249C, 311C	K	n.d.
HAL1	Halberstadt	Germany	298C	V	V
HAL2	Halberstadt	Germany	086C, 147a, 172C, 223T, 248T, 320T, 355T	N1a	n.d.
HAL3	Halberstadt	Germany	093C, 126C, 294T, 296T, 304C	T2	n.d.
SCHWE1	Schwetzingen	Germany	126C, 294T, 296T, 304C	T2	n.d.
SCHWE2	Schwetzingen	Germany	126C, 292T, 294T, 296T	T2	n.d.
SCHWE4	Schwetzingen	Germany	286T, 304C	H	H
SCHWE5	Schwetzingen	Germany	126C, 294T, 296T	T2	n.d.
SEE1	Seehausen	Germany	069T, 126C	J	n.d.
UNS2	Unseburg	Germany	223T, 292T	W	n.d.
UWS2	Unterwiederstedt	Germany	224C, 249C, 311C	K	n.d.
UWS5	Unterwiederstedt	Germany	129A, 147a, 154C, 172C, 223T, 248T, 320T, 355T	N1a	n.d.
VAI3	Vaihingen an der Enz	Germany	319A, 343G	U3	n.d.

n.d.= not determined

1. Bramanti B, Thomas MG, Haak W, Unterlaender M, Jores P, et al. (2009) Genetic discontinuity between local hunter-gatherers and central Europe's first farmers. Science 326: 137-140.
2. Haak W, Forster P, Bramanti B, Matsumura S, Brandt G, et al. (2005) Ancient DNA from the first European farmers in 7500-year-old Neolithic sites. Science 310: 1016-1018.

3. Haak W (2006) Populationsgenetik der ersten Bauern Mitteleuropas. Eine aDNA-Studie an neolithischem Skelettmaterial. PhD Thesis, University Mainz.
