# Supplementary material for: Ancient DNA from European Early Neolithic Farmers Reveals Their Near Eastern Affinities
Source: PLoS Biol. 2010 Nov 9;8(11):e1000536. doi: 10.1371/journal.pbio.1000536 (PMC2976717; doi:10.1371/journal.pbio.1000536)
Supplement: Table S6 — Details of Neolithic and modern-day populations used for comparison. (0.14 MB XLS) [file pbio.1000536.s010.doc]

**Table S6. Details of Neolithic and modern-day populations used for comparison.**

| **Population** | **Shortcut** | **N** | **Long** | **Lat** | **Analyses*** | **References/Genbank Acc. No.** |
| --- | --- | --- | --- | --- | --- | --- |
| ancient | | | | | | |
| Derenburg | DEB22 | 22 | 11.03 | 51.88 | S,M,P,D | [1], this study |
| LBK excluding Derenburg | LBK20 | 20 |  |  | S,M,P | (1) |
| LBK | LBK42 | 42 |  |  | S,M,P,D,BC/BN |  |
| LBK excluding kin | LBK34kin | 34 |  |  | S,M,P |  |
| Mesolithic hunter-gatherers | HG | 20 |  |  | S,M,P,D,BC | [2] |
| modern | | | | | | |
| Albanians | ALB | 281 | 19.75 | 41.38 | S,M,P,FE,D | [3,4], EBC** |
| Armenians | ARM | 192 | 44.53 | 40.18 | S,M,P,FN,D | AJ233203-AJ233395 |
| Aromuns | ARO | 133 | 21.25 | 41.35 | S,M,P,FE,D | [4] |
| Austrians | AUT | 117 | 11.37 | 47.35 | S,M,P,FE,D | [5,6] |
| Azeris | AZE | 88 | 48 | 40 | S,M,P,FN,D | [7,8] |
| Basques | BAS | 106 | 357.20 | 42.97 | S,M,P,FE,D | [9,10] |
| Bosnians | BOS | 322 | 18.40 | 43.87 | S,M,P,FE,D | [11], AY005485-AY005664 |
| Bulgarians | BUL | 141 | 26.13 | 42.93 | S,M,P,FE,D | [8,12] |
| Byelorussians | BLR | 352 | 29.20 | 53.12 | S,M,P,FE,D | [13], LHPG*** |
| Chuvash | CHV | 92 | 46.77 | 55.97 | S,M,P,FE,D | [8,14] |
| Croats | CRO | 980 | 17.02 | 43.28 | S,M,P,FE,D | [15], EBC |
| Czechs | CZE | 449 | 14.15 | 50.10 | S,M,P,FE,D,BC | [8,16,17], EBC |
| English | ENG | 1297 | 358 | 53 | S,M,P,FE,D | [8,18,19] |
| Estonians | EST | 497 | 26.70 | 58.37 | S,M,P,FE,D | EBC |
| Finns | FIN | 508 | 26.00 | 62.00 | S,M,P,FE,D | [20,21,22] |
| French | FRA | 998 | 0.07 | 47.33 | S,M,P,FE,D | [23,24] |
| Georgians | GEO | 158 | 44 | 42 | S,M,P,FN,D,BN | [7], EBC |
| Germans | DEU | 1406 | 9.60 | 52.57 | S,M,P,FE,D,BC | [8,25,26,27,28] |
| Greeks | GRC | 741 | 22.85 | 40.53 | S,M,P,FE,D,BN | [4,8,29,30], EBC |
| Hungarians | HUN | 190 | 19.00 | 47.50 | S,M,P,FE,D | [30], EBC |
| Icelanders | ISL | 448 | 337.40 | 64.50 | S,M,P,FE,D | [18,31,32] |
| Iranians | IRN | 517 | 51.40 | 35.68 | S,M,P,FN,D,BN | [7,33] |
| Iraqis | IRQ | 168 | 44.40 | 33.33 | S,M,P,FN,D,BN | [8,34] |
| Irish | IRL | 300 | 350.88 | 53.47 | S,M,P,FE,D | [35] |
| Italians | ITA | 830 | 12.48 | 41.90 | S,M,P,FE,D | [8,15,36,37,38,39], DQ081608-DQ081668 |
| Jordanians | JOR | 146 | 35.82 | 31.78 | S,M,P,FN,D,BN | [40] |
| Kabardinians | KAB | 163 | 43 | 43 | S,M,P,FN,D,BN | [8], EBC |
| Karelians | KAR | 83 | 34.30 | 63.72 | S,M,P,FE,D | [31] |
| Komi | KOM | 127 | 53.00 | 61.00 | S,M,P,FE,D | [14] |
| Kurds | KUR | 73 | 43.07 | 37.55 | S,M,P,FN,D | [7,8] |
| Latvians | LVA | 299 | 24 | 57 | S,M,P,FE,D | [41] |
| Lithuanians | LTU | 180 | 24 | 55 | S,M,P,FE,D | [42], EBC |
| Mari | CHM | 136 | 48.10 | 55.95 | S,M,P,FE,D | [14] |
| Moroccans | MAR | 336 | 353.12 | 30.97 | S,M,P,FN,D | [43], EBC |
| Mordvinians | MOR | 99 | 44.47 | 54.30 | S,M,P,FE,D | [14] |
| Norwegians | NOR | 663 | 10.57 | 59.88 | S,M,P,FE,D | [8,44,45,46,47] |
| Ossetians | OSS | 289 | 44.5 | 43 | S,M,P,FE,D | [8], EBC |
| Palestinians | PSE | 117 | 35.10 | 31.82 | S,M,P,FN,D | [8] |
| Poles | POL | 583 | 18.5 | 53 | S,M,P,FE,D,BC | [8,48], EBC |
| Portuguese | PRT | 848 | 351.97 | 39.48 | S,M,P,FE,D | [49,50] |
| Romanians | ROU | 197 | 28.58 | 44.13 | S,M,P,FE,D | [4,8] |
| Russians | RUS | 918 | 32.88 | 53.93 | S,M,P,FE,D | [8,13,51], LHPG, EBC AY959517-AY959667  AY959713-AY959798 |
| Sardinians | SAR | 115 | 9.00 | 40.00 | S,M,P,FE,D | [8] |
| Saudi Arabians | SAU | 325 | 46.48 | 24.62 | S,M,P,FN,D | [52], EBC |
| Scots | SCO | 1121 | 355 | 58 | S,M,P,FE,D | [44] |
| Sicilians | SIC | 106 | 14.17 | 37.62 | S,M,P,FE,D | [53] |
| Slovaks | SVK | 510 | 19.33 | 48.83 | S,M,P,FE,D | EBC |
| Slovenians | SVN | 233 | 14.5 | 46.07 | S,M,P,FE,D | [11,54,55] |
| Spanish | ESP | 704 | 356.17 | 40.63 | S,M,P,FE,D | [9,49,55,56,57,58] |
| Swedes | SWE | 471 | 17.73 | 59.33 | S,M,P,FE,D | EBC |
| Swiss | CHE | 230 | 6.58 | 46.68 | S,M,P,FE,D | [59,60] |
| Syrians | SYR | 169 | 36.20 | 33.58 | S,M,P,FN,D | [8], EBC |
| Tatars | TAT | 225 | 52.38 | 55.30 | S,M,P,FE,D | [14] |
| Turkish | TUR | 608 | 33.00 | 39.00 | S,M,P,FN,D,BN | [7,8,12], EBC |
| Ukrainians | UKR | 610 | 32.07 | 49.43 | S,M,P,FE,D | LHPG, EBC |

*S=shared haplotype analyses; M=MDS; P=PCA; FE=Hg frequency pool Europe, FN=Hg frequency pool Near East; D=mapped genetic distances; BC=BayeSSC pool Central Europe, BN=BayeSSC pool Near East

**EBC - data of Estonian Biocentre, unpublished

***LHPG - data of Laboratory for Human Population Genetics (Research Centre for Medical Genetics RAMS), unpublished

**References**

1. Haak W, Forster P, Bramanti B, Matsumura S, Brandt G, et al. (2005) Ancient DNA from the first European farmers in 7500-year-old Neolithic sites. Science 310: 1016-1018.

2. Bramanti B, Thomas MG, Haak W, Unterlaender M, Jores P, et al. (2009) Genetic discontinuity between local hunter-gatherers and central Europe's first farmers. Science 326: 137-140.

3. Belledi M, Poloni ES, Casalotti R, Conterio F, Mikerezi I, et al. (2000) Maternal and paternal lineages in Albania and the genetic structure of Indo-European populations. Eur J Hum Genet 8: 480-486.

4. Bosch E, Calafell F, Gonzalez-Neira A, Flaiz C, Mateu E, et al. (2006) Paternal and maternal lineages in the Balkans show a homogeneous landscape over linguistic barriers, except for the isolated Aromuns. Ann Hum Genet 70: 459-487.

5. Parson W, Parsons TJ, Scheithauer R, Holland MM (1998) Population data for 101 Austrian Caucasian mitochondrial DNA d-loop sequences: application of mtDNA sequence analysis to a forensic case. Int J Legal Med 111: 124-132.

6. Handt O, Richards M, Trommsdorff M, Kilger C, Simanainen J, et al. (1994) Molecular genetic analyses of the Tyrolean Ice Man. Science 264: 1775-1778.

7. Quintana-Murci L, Chaix R, Wells RS, Behar DM, Sayar H, et al. (2004) Where west meets east: the complex mtDNA landscape of the southwest and Central Asian corridor. Am J Hum Genet 74: 827-845.

8. Richards M, Macaulay V, Hickey E, Vega E, Sykes B, et al. (2000) Tracing European founder lineages in the Near Eastern mtDNA pool. Am J Hum Genet 67: 1251-1276.

9. Corte-Real HB, Macaulay VA, Richards MB, Hariti G, Issad MS, et al. (1996) Genetic diversity in the Iberian Peninsula determined from mitochondrial sequence analysis. Ann Hum Genet 60: 331-350.

10. Bertranpetit J, Sala J, Calafell F, Underhill PA, Moral P, et al. (1995) Human mitochondrial DNA variation and the origin of Basques. Ann Hum Genet 59 ( Pt 1): 63-81.

11. Malyarchuk BA, Grzybowski T, Derenko MV, Czarny J, Drobnic K, et al. (2003) Mitochondrial DNA variability in Bosnians and Slovenians. Ann Hum Genet 67: 412-425.

12. Calafell F, Underhill P, Tolun A, Angelicheva D, Kalaydjieva L (1996) From Asia to Europe: mitochondrial DNA sequence variability in Bulgarians and Turks. Ann Hum Genet 60 ( Pt 1): 35-49.

13. Belyaeva O, Bermisheva M, Khrunin A, Slominsky P, Bebyakova N, et al. (2003) Mitochondrial DNA Variations in Russian and Belorussian. Hum Biol 75: 647-660.

14. Bermisheva M, Tambets K, Villems R, Khusnutdinova E (2002) [Diversity of mitochondrial DNA haplotypes in ethnic populations of the Volga-Ural region of Russia]. Mol Biol (Mosk) 36: 990-1001.

15. Babalini C, Martinez-Labarga C, Tolk HV, Kivisild T, Giampaolo R, et al. (2005) The population history of the Croatian linguistic minority of Molise (southern Italy): a maternal view. Eur J Hum Genet 13: 902-912.

16. Vanecek T, Vorel F, Sip M (2004) Mitochondrial DNA D-loop hypervariable regions: Czech population data. Int J Legal Med 118: 14-18.

17. Malyarchuk BA, Vanecek T, Perkova MA, Derenko MV, Sip M (2006) Mitochondrial DNA variability in the Czech population, with application to the ethnic history of Slavs. Hum Biol 78: 681-696.

18. Richards M, Corte-Real H, Forster P, Macaulay V, Wilkinson-Herbots H, et al. (1996) Paleolithic and neolithic lineages in the European mitochondrial gene pool. Am J Hum Genet 59: 185-203.

19. Sykes B (2006) Blood of the Isles: Exploring the Genetic Roots of Our Tribal History: Bantam. 306 p.

20. Meinila M, Finnila S, Majamaa K (2001) Evidence for mtDNA admixture between the Finns and the Saami. Hum Hered 52: 160-170.

21. Lahermo P, Sajantila A, Sistonen P, Lukka M, Aula P, et al. (1996) The genetic relationship between the Finns and the Finnish Saami (Lapps): analysis of nuclear DNA and mtDNA. Am J Hum Genet 58: 1309-1322.

22. Kittles RA, Bergen AW, Urbanek M, Virkkunen M, Linnoila M, et al. (1999) Autosomal, mitochondrial, and Y chromosome DNA variation in Finland: evidence for a male-specific bottleneck. Am J Phys Anthropol 108: 381-399.

23. Richard C, Pennarun E, Kivisild T, Tambets K, Tolk HV, et al. (2007) An mtDNA perspective of French genetic variation. Ann Hum Biol 34: 68-79.

24. Dubut V, Chollet L, Murail P, Cartault F, Beraud-Colomb E, et al. (2004) mtDNA polymorphisms in five French groups: importance of regional sampling. Eur J Hum Genet 12: 293-300.

25. Poetsch M, Wittig H, Krause D, Lignitz E (2003) Mitochondrial diversity of a northeast German population sample. Forensic Sci Int 137: 125-132.

26. Pfeiffer H, Brinkmann B, Huhne J, Rolf B, Morris AA, et al. (1999) Expanding the forensic German mitochondrial DNA control region database: genetic diversity as a function of sample size and microgeography. Int J Legal Med 112: 291-298.

27. Lutz S, Weisser HJ, Heizmann J, Pollak S (1998) Location and frequency of polymorphic positions in the mtDNA control region of individuals from Germany. Int J Legal Med 111: 67-77.

28. Baasner A, Schafer C, Junge A, Madea B (1998) Polymorphic sites in human mitochondrial DNA control region sequences: population data and maternal inheritance. Forensic Sci Int 98: 169-178.

29. Irwin J, Saunier J, Strouss K, Paintner C, Diegoli T, et al. (2008) Mitochondrial control region sequences from northern Greece and Greek Cypriots. Int J Legal Med 122: 87-89.

30. Bogacsi-Szabo E, Kalmar T, Csanyi B, Tomory G, Czibula A, et al. (2005) Mitochondrial DNA of ancient Cumanians: culturally Asian steppe nomadic immigrants with substantially more western Eurasian mitochondrial DNA lineages. Hum Biol 77: 639-662.

31. Sajantila A, Lahermo P, Anttinen T, Lukka M, Sistonen P, et al. (1995) Genes and languages in Europe: an analysis of mitochondrial lineages. Genome Res 5: 42-52.

32. Helgason A, Sigurdardottir S, Gulcher JR, Stefansson K, Ward R, et al. (2000) Sampling Saturation and the European MtDNA Pool: Implications for Detecting Genetic Relationships among Populations. Archaeogenetics: DNA and the population prehistory of Europe. Cambridge: McDonald Institute for Archaeological Research. pp. 285-294.

33. Metspalu M, Kivisild T, Metspalu E, Parik J, Hudjashov G, et al. (2004) Most of the extant mtDNA boundaries in South and Southwest Asia were likely shaped during the initial settlement of Eurasia by anatomically modern humans. BMC Genetics 5: 26. doi:10.1186/1471-2156-5-26

34. Al-Zahery N, Semino O, Benuzzi G, Magri C, Passarino G, et al. (2003) Y-chromosome and mtDNA polymorphisms in Iraq, a crossroad of the early human dispersal and of post-Neolithic migrations. Mol Phylogenet Evol 28: 458-472.

35. McEvoy B, Richards M, Forster P, Bradley DG (2004) The Longue Duree of genetic ancestry: multiple genetic marker systems and Celtic origins on the Atlantic facade of Europe. Am J Hum Genet 75: 693-702.

36. Tagliabracci A, Turchi C, Buscemi L, Sassaroli C (2001) Polymorphism of the mitochondrial DNA control region in Italians. Int J Legal Med 114: 224-228.

37. Achilli A, Olivieri A, Pala M, Metspalu E, Fornarino S, et al. (2007) Mitochondrial DNA variation of modern Tuscans supports the near eastern origin of Etruscans. Am J Hum Genet 80: 759-768.

38. Francalacci P, Bertranpetit J, Calafell F, Underhill PA (1996) Sequence diversity of the control region of mitochondrial DNA in Tuscany and its implications for the peopling of Europe. Am J Phys Anthropol 100: 443-460.

39. Mogentale-Profizi N, Chollet L, Stevanovitch A, Dubut V, Poggi C, et al. (2001) Mitochondrial DNA sequence diversity in two groups of Italian Veneto speakers from Veneto. Ann Hum Genet 65: 153-166.

40. Gonzalez AM, Karadsheh N, Maca-Meyer N, Flores C, Cabrera VM, et al. (2008) Mitochondrial DNA variation in Jordanians and their genetic relationship to other Middle East populations. Ann Hum Biol 35: 212-231.

41. Pliss L, Tambets K, Loogvali EL, Pronina N, Lazdins M, et al. (2006) Mitochondrial DNA portrait of Latvians: towards the understanding of the genetic structure of Baltic-speaking populations. Ann Hum Genet 70: 439-458.

42. Kasperaviciute D, Kucinskas V, Stoneking M (2004) Y chromosome and mitochondrial DNA variation in Lithuanians. Ann Hum Genet 68: 438-452.

43. Rando JC, Pinto F, Gonzalez AM, Hernandez M, Larruga JM, et al. (1998) Mitochondrial DNA analysis of northwest African populations reveals genetic exchanges with European, near-eastern, and sub-Saharan populations. Ann Hum Genet 62 ( Pt 6): 531-550.

44. Helgason A, Hickey E, Goodacre S, Bosnes V, Stefansson K, et al. (2001) mtDNA and the Islands of the North Atlantic: Estimating the Proportions of Norse and Gaelic Ancestry. Am J Hum Genet 68: 723-737.

45. Passarino G, Cavalleri GL, Lin AA, Cavalli-Sforza LL, Borresen-Dale AL, et al. (2002) Different genetic components in the Norwegian population revealed by the analysis of mtDNA and Y chromosome polymorphisms. Eur J Hum Genet 10: 521-529.

46. Opdal SH, Rognum TO, Vege A, Stave AK, Dupuy BM, et al. (1998) Increased number of substitutions in the D-loop of mitochondrial DNA in the sudden infant death syndrome. Acta Paediatr 87: 1039-1044.

47. Dupuy BM, Olaisen B (1996) mtDNA sequences in the Norwegian Saami and main populations. In: Carracedo A, Brinkmann B, Bär W, editors. Advances in forensic haemogenetics. Berlin: Springer. pp. 23-25.

48. Malyarchuk BA, Grzybowski T, Derenko MV, Czarny J, Wozniak M, et al. (2002) Mitochondrial DNA variability in Poles and Russians. Ann Hum Genet 66: 261-283.

49. Gonzalez AM, Brehm A, Perez JA, Maca-Meyer N, Flores C, et al. (2003) Mitochondrial DNA affinities at the Atlantic fringe of Europe. Am J Phys Anthropol 120: 391-404.

50. Pereira L, Cunha C, Amorim A (2004) Predicting sampling saturation of mtDNA haplotypes: an application to an enlarged Portuguese database. Int J Legal Med 118: 132-136.

51. Kornienko IV, Vodolazhskii DI, Afanas'eva GV, Ivanov PL (2004) [Polymorphism of the central region of D-loop of mitochondrial DNA and personality identification by forensic medicine methods]. Sud Med Ekspert 47: 27-32.

52. Abu-Amero KK, Larruga JM, Cabrera VM, Gonzalez AM (2008) Mitochondrial DNA structure in the Arabian Peninsula. BMC Evol Biol 8: 45.

53. Cali F, Le Roux MG, D'Anna R, Flugy A, De Leo G, et al. (2001) MtDNA control region and RFLP data for Sicily and France. Int J Legal Med 114: 229-231.

54. Zupanic Pajnic I, Balazic J, Komel R (2004) Sequence polymorphism of the mitochondrial DNA control region in the Slovenian population. Int J Legal Med 118: 1-4.

55. Larruga JM, Diez F, Pinto FM, Flores C, Gonzalez AM (2001) Mitochondrial DNA characterisation of European isolates: the Maragatos from Spain. Eur J Hum Genet 9: 708-716.

56. Salas A, Comas D, Lareu MV, Bertranpetit J, Carracedo A (1998) mtDNA analysis of the Galician population: a genetic edge of European variation. Eur J Hum Genet 6: 365-375.

57. Crespillo M, Luque JA, Paredes M, Fernandez R, Ramirez E, et al. (2000) Mitochondrial DNA sequences for 118 individuals from northeastern Spain. Int J Legal Med 114: 130-132.

58. Maca-Meyer N, Sanchez-Velasco P, Flores C, Larruga JM, Gonzalez AM, et al. (2003) Y chromosome and mitochondrial DNA characterization of Pasiegos, a human isolate from Cantabria (Spain). Ann Hum Genet 67: 329-339.

59. Dimo-Simonin N, Grange F, Taroni F, Brandt-Casadevall C, Mangin P (2000) Forensic evaluation of mtDNA in a population from south west Switzerland. Int J Legal Med 113: 89-97.

60. Pult I, Sajantila A, Simanainen J, Georgiev O, Schaffner W, et al. (1994) Mitochondrial DNA sequences from Switzerland reveal striking homogeneity of European populations. Biol Chem Hoppe Seyler 375: 837-840.
