# Supplementary material for: Ancient DNA from European Early Neolithic Farmers Reveals Their Near Eastern Affinities
Source: PLoS Biol. 2010 Nov 9;8(11):e1000536. doi: 10.1371/journal.pbio.1000536 (PMC2976717; doi:10.1371/journal.pbio.1000536)
Supplement: Table S7 — GenoCoRe22 and GenoY25 multiplex assay and additional Y chromosome PCR primer information. (0.24 MB XLS) [file pbio.1000536.s011.doc]

**Table S7**. GenoCoRe22 and GenoY25 multiplex assay and additional Y-chromosome PCR primer information.

| **GenoCoRe22 PCR primer information** | | | | | | | |
| --- | --- | --- | --- | --- | --- | --- | --- |
| μM | Name | Sequence 5’-3’ | Name | Sequence 5’-3’ | Amplicon size (bp) | Haplogroup | Targeted SNP sites |
| 0.022 | L02727 | AACACAGCAAGACGAGAAGACC | H02760 | GGACCTGTGGGTTTGTTAGGT | 75 | L2’3’4’6’7 | 2758 |
| 0.088 | L03585 | CCCCTCCCCATACCCAAC | H03601 | GGCTAGAATAAATAGGAGGCCTAGGTT | 60 | L | 3594 |
| 0.051 | L04237 | TGATATGTCTCCATACCCATTACAA | H04253 | CTTTTATCAGACATATTTCTTAGGTTTGAG | 70 | A | 4248 |
| 0.051 | L04578 | TTACCTGAGTAGGCCTAGAAATAAACA | H04618 | GCAGCTTCTGTGGAACGAG | 85 | V,M3 | 4580 |
| 0.015 | L05171 | ACCCTACTACTATCTCGCACCTGA | H05204 | CTAGGGAGAGGAGGGTGGAT | 76 | D | 5178 |
| 0.011 | L06363 | ACCATCTTCTCCTTACACCTAGCAG | H06378 | GATGAAATTGATGGCCCCTAA | 60 | X | 6371 |
| 0.051 | L07003 | GCAAACTCATCACTAGACATCGTACT | H07029 | CCTATTGATAGGACATAGTGGAAGTG | 77 | H | 7028 |
| 0.015 | L08970 | CATACTAGTTATTATCGAAACCATCAGC | H09003 | CTGCAGTAATGTTAGCGGTTAGG | 83 | W | 8994 |
| 0.059 | L10025 | CTTTTAGTATAAATAGTACCGTTAACTTCCAA | H10037 | AAGTTTATTACTCTTTTTTGAATGTTGTCA | 73 | I | 10034 |
| 0.059 | L10228 | TCCCTTTCTCCATAAAATTCTTCTT | H10249 | AGGAGGGCAATTTCTAGATCAAATA | 70 | N1 | 10238 |
| 0.037 | L10382 | AAGTCTGGCCTATGAGTGACTACAA | H10421 | TGAGTCGAAATCATTCGTTTTG | 85 | M | 10400 |
| 0.029 | L10548 | GAATACTAGTATATCGCTCACACCTCA | H10558 | GCGATAGTATTATTCCTTCTAGGCATAGTA | 66 | K | 10550 |
| 0.051 | L10870 | CCACAGCCTAATTATTAGCATCATC | H10888 | GCTAAATAGGTTGTTGTTGATTTGG | 67 | N | 10873 |
| 0.015 | L11454 | ATCGCTGGGTCAATAGTACTTGC | H11479 | TGAGTGTGAGGCGTATTATACCATAG | 73 | U | 11467 |
| 0.037 | L11710 | GGCGCAGTCATTCTCATAATC | H11735 | AGTTTGAGTTTGCTAGGCAGAATAG | 70 | preHV | 11719 |
| 0.037 | L12611 | CTACTTCTCCATAATATTCATCCCTGT | H12621 | AATTCTATGATGGACCATGTAACG | 60 | J | 12612 |
| 0.037 | L12689 | CAGACCCAAACATTAATCAGTTCTT | H12715 | TGTTAGCGGTAACTAAGATTAGTATGGT | 78 | R | 12705 |
| 0.051 | L13258 | ATCGTAGCCTTCTCCACTTCAA | H13295 | AGGAATGCTAGGTGTGGTTGGT | 80 | C | 13263 |
| 0.022 | L13350 | CACGCCTTCTTCAAAGCCATA | H13372 | GTTCATTGTTAAGGTTGTGGATGAT | 67 | T | 13368 |
| 0.051 | L14759 | AGAACACCAATGACCCCAATAC | H14799 | GGTGGGGAGGTCGATGA | 78 | HV | 14766 |
| 0.037 | L13923 | TTTCTCCAACATACTCGGATTCTAC | H13942 | AGAAGGCCTAGATAGGGGATTGT | 66 | R9 | 13928 |
| 0.037 | L08268 | AATAGGGCCCGTATTTACCCTATA | H08295 | AGGTTAATGCTAAGTTAGCTTTACAGTG | 78 | B | 8280del |

**Table S7. Continued.**

| **GenoCoRe22 multiplex assay SBE primer information** | | | | | |
| --- | --- | --- | --- | --- | --- |
| μM | Name | Sequence 5’-3’ | size in bp | Nucleotides detected | Detection orientation |
| 0.050 | 13928snR | tctctctctctctGATTGTGCGGTGTGTGATG | 32 | C>G OR T | Reverse |
| 0.050 | 3594snR | ctctctctctctctctctGAGGCCTAGGTTGAGGTT | 36 | G>A | Reverse |
| 0.050 | 10550snR | ctctctctctctctctTCTAGGCATAGTAGGGAGGA | 36 | T>C | Reverse |
| 0.050 | 11467snF | ctctctctctctctctctctGTACTTGCCGCAGTACTCTT | 40 | A>G | Forward |
| 0.050 | 4248snF | tctctctctctctctctATACCCATTACAATCTCCAGCAT | 40 | T>C | Forward |
| 0.050 | 8994snF | ctctctctctctctctctctctTACTCATTCAACCAATAGCCCT | 44 | G>A | Forward |
| 0.050 | 13263snR | ctctctctctctctctctCCGATTGTAACTATTATGAGTCCTAG | 44 | T>C | Reverse |
| 0.050 | 13368snF | ctctctctctctctctctctctctctCCATACTATTTATGTGCTCCGG | 48 | G>A | Forward |
| 0.050 | 11719snR | tctctctctctctctctctctTAGGCAGAATAGTAATGAGGATGTAAG | 48 | C>T | Reverse |
| 0.050 | 4580snR | ctctctctctctctctctctctctTTTTGGTTAGAACTGGAATAAAAGCTAG | 52 | C>T | Reverse |
| 0.050 | 8280delsnR | tctctctctctctctctctctctctctCTTTACAGTGGGCTCTAGAGGGGGT | 52 | A>G(9del) | Reverse |
| 0.050 | 6371snR | tctctctctctctctctctctctctctctctctAAATTGATGGCCCCTAAGATAGA | 56 | G>A | Reverse |
| 0.050 | 10238snF | tctctctctctctctctctctctctCTTTCTCCATAAAATTCTTCTTAGTAGCTAT | 56 | T>C | Forward |
| 0.050 | 7028snR | tctctctctctctctctctctctctctctctctctCTATTGATAGGACATAGTGGAAGTG | 60 | G>A | Reverse |
| 0.050 | 10034snF | ctctctctctctctctctctctctctGTATAAATAGTACCGTTAACTTCCAATTAACTAG | 60 | T>C | Forward |
| 0.050 | 5178snR | tctctctctctctctctctctctctctctctctctctctGGAATTAAGGGTGTTAGTCATGTTA | 64 | G>T | Reverse |
| 0.050 | 14766snR | ctctctctctctctctctctctctctctctctctctctGAGTGGTTAATTAATTTTATTAGGGGGTTA | 68 | G>A | Reverse |
| 0.050 | 12705snR | ctctctctctctctctctctctctctctctctctctctctctGGTAACTAAGATTAGTATGGTAATTAGGAA | 72 | G>A | Reverse |
| 0.050 | 10873snR | ctctctctctctctctctctctctctctctctctctctctctctGTTGTTGTTGATTTGGTTAAAAAATAGTAG | 74 | A>G | Reverse |
| 0.050 | 12612snF | ctctctctctctctctctctctctctctctctctctctctctctctctctCTACTTCTCCATAATATTCATCCCTGT | 77 | A>G | Forward |
| 0.050 | 2758snF | ctctctctctctctctctctctctctctctctctctctctctctctctctCTATGGAGCTTTAATTTATTAATGCAAACA | 80 | G>A | Forward |
| 0.050 | 10400snR | ctctctctctctctctctctctctctctctctctctctctctctctctAAATCATTCGTTTTGTTTAAACTATATACCAATTC | 83 | G>A | Reverse |

**Table S7. Continued.**

| **GenoY25 multiplex assay PCR primer information** | | | | | | | |
| --- | --- | --- | --- | --- | --- | --- | --- |
| μM | Name | Sequence 5'-3' | Name | Sequence 5'-3' | size in bp | Haplogroup | SNP name |
| 0.02 | M174F | ATGTATCAAATCGCTTCTCTGAATAC | M174R | CAAATGCACCCCTCACTTCT | 66 | D | M174 |
| 0.02 | M231F | AACAACATTTACTGTTTCTACTGCTTTC | M231R | CAGAAATTACAGGTATGAATTCTTTGAC | 73 | N | M231 |
| 0.01 | M242F | ATAGAAAGTTTGTGCAAAAAGGTGA | M242R | AAAAACACGTTAAGACCAATGC | 61 | Q | M242 |
| 0.01 | M168F | GTGGAGTATGTGTTGGAGGTGA | M168R | CATCTCTTACCCAAACTGCTAAAAC | 74 | CR | M168 |
| 0.02 | S21F | GCATAGGGATTCCTGAATAGCAAAT | S21R | GTCGGGGAAGGCAGGTA | 79 | R1b1b1a | S21 |
| 0.02 | M45F | AGAGAGGATATCAAAAATTGGCAGT | M45R | GCCTGGACCTCAGAAGGAG | 68 | P | M45 |
| 0.02 | SRY10831F | CAGTATCTGGCCTCTTGTATCTGAC | SRY10831R | CACCACATAGGTGAACCTTGAA | 68 | BR/R1a | SRY10831 |
| 0.01 | M2F | GCTCCCCTGTTTAAAAATGTAGGT | M2R | CCCCCTTTATCCTCCACAGAT | 83 | E1b1a | M2 |
| 0.01 | M89F | TTCAGCTCTCTTCCTAAGGTTATGT | M89R | GTAGCTGCAACTCAGGCAAAGT | 65 | F | M89 |
| 0.02 | M267F | ACCAAGTCTGGATAGCGGATT | M267R | CAGCTAGATTGTGTTCTTCCACAC | 83 | J1 | M267 |
| 0.01 | M343F | TTAAATATGCAAATGCAGAGTGC | M343R | ACGTGCCTGGCAGCATAG | 78 | R1b | M343 |
| 0.02 | M304F | TTGTAACAAACAGTATGTGGGATTT | M304R | CGTCTTATACCAAAATATCACCAGTT | 88 | J | M304 |
| 0.02 | M172F | CCAAACCCATTTTGATGCTT | M172R | CCAGGTACAGAGAAAGTTTGGACT | 87 | J2 | M172 |
| 0.03 | M175F | AGGCACATGCCTTCTCACTT | M175R | TTTCTACTGATACCTTTGTTTCTGTTC | 61 | O | M175 |
| 0.07 | M35F | CAATACTCAGTGTCCCAATTTTCCT | M35R | ACTTTCGGAGTCTCTGCCTGT | 61 | E1b1b1 | M35 |
| 0.03 | M170F | GTTTTCATATTCTGTGCATTATACAAATTAC | M170R | GTGAGACACAACCCACACTGAA | 87 | I | M170 |
| 0.03 | M201F | CTCAGATCTAATAATCCAGTATCAACTGA | M201R | CCTATCAGCTTCATCCAACACTAA | 72 | G | M201 |
| 0.04 | M269F | GGGAATGATCAGGGTTTGGTTA | M269R | GCCTTCTGAGGCACATATGATAA | 74 /75 | R1b1b | M269 |
| 0.02 | M122F | GCCTTTTGGAAATGAATAAATCAAG | M122R | CTTTATTCAGATTTTCCCCTGAGA | 80 | O3 | M122 |
| 0.01 | M17F | TCACCAGAGTTTGTGGTTGC | M17R | TCACAAAAATAGTTTGGCCACTT | 85 | R1a1 | M17 |
| 0.02 | M96F | GTGTAACTTGGAAAACAGGTCTCTC | M96R | AAGGACCATATATTTTGCCATAGGT | 85 | E | M96 |
| 0.02 | M78F | CATGAACACAAATTGATACACTTAACA | M78R | CAAGTACTATGACCAGCTTATTTTGAA | 83 | E1b1b1a | M78 |
| 0.02 | M9F | AGAAACGGCCTAAGATGGTTG | M9R | AACTAAGTATGTAAGACATTGAACGTTTG | 84 | K | M9 |
| 0.02 | M207F | GGGGCAAATGTAAGTCAAGC | M207R | TCACTTCAACCTCTTGTTGGAA | 83 | R | M207 |
| 0.04 | M216F | GCTAGAAAAAAATTCCTTTATTAAAGAAATGTA | M216R | TTCTAAATCTGAATTCTGACACTGC | 83 | C | M216 |

**Table S7. Continued.**

| **GenoY25 multiplex assay SBE primer information** | | | | | |
| --- | --- | --- | --- | --- | --- |
| μM | Name | Sequence 5'-3' | Size in bp | Alleles Anc>der | Detection orientation |
| 0.06 | M174snF | tctctctctctctAATACCTTCTGGAGTGCCC | 32 | T>C | Forward |
| 0.10 | M231snF | ctctctctAACAACATTTACTGTTTCTACTGCTTTC | 36 | G>A | Forward |
| 0.10 | M242snF | ctctctctctctctctAAAAGGTGACCAAGGTGCT | 36 | C>T | Forward |
| 0.06 | M168snR | tctctctctCTAAAACTATTGTTTTAATTCTTCAGCTAGC | 40 | G>A | Reverse |
| 0.10 | S21snF | tctctctctctctctctctATAGCAAATCCCAAAGCTCCA | 40 | C>T | Forward |
| 0.13 | M45snF | ctctctctctGATATCAAAAATTGGCAGTGAAAAATTATAGATA | 44 | G>A | Forward |
| 0.06 | SRY10831snR | ctctctctctctctctctCACATAGGTGAACCTTGAAAATGTTA | 44 | T>C or C>T | Reverse |
| 0.06 | M2snF | tctctctctctctGTTTTATTATTATATTTCATTGTTAACAAAAGTCC | 48 | A>G | Forward |
| 0.06 | M89snF | ctctctctctctctctctctCTCTTCCTAAGGTTATGTACAAAAATCT | 48 | C>T | Forward |
| 0.06 | M267snR | ctctctctctctctctctctctctctctCTTCCACACAAAATACTGAACGT | 52 | A>C | Reverse |
| 0.10 | M343snR | tctctctctctctctctctctctctctctctctCCCACATATCTCCAGGTGT | 52 | G>T | Reverse |
| 0.13 | M304snF | ctctctctctctctctctctctctctctATGTGTTCAATTTGAAAGTAACTTGTGA | 56 | A>C | Forward |
| 0.04 | M172snF | ctctctctctctctctctctctctctctctctctctCCAAACCCATTTTGATGCTT | 56 | T>G | Forward |
| 0.08 | M175snF | ctctctctctctctctctctctctctctctctctctctctCACATGCCTTCTCACTTCTC | 60 | T>A | Forward |
| 0.21 | M35snF | tctctctctctctctctctctctctctctctctctctctCAATTTTCCTTTGGGACACTG | 60 | G>C | Forward |
| 0.15 | M170snF | tctctctctctctctctctctctctctctctctctCTATTTTATTTACTTAAAAATCATTGTTC | 64 | A>C | Forward |
| 0.10 | M201snF | ctctctctctctctctctctctctctctctctctctAGATCTAATAATCCAGTATCAACTGAGG | 64 | G>T | Forward |
| 0.15 | M269snR | tctctctctctctctctctctctctctctctctATATGATAAAAAAAAAATTGTTTTCAATTTACCAG | 68 | A>G | Reverse |
| 0.15 | M122snF | tctctctctctctctctctctctctctctctctctctctGGTAGAAAAGCAATTGAGATACTAATTCA | 68 | T>C | Forward |
| 0.13 | M17snR | ctctctctctctctctctctctctctctctctctctctctctctctctCCAAAATTCACTTAAAAAAACCC | 71 | C>G | Reverse |
| 0.06 | M96snF | ctctctctctctctctctctctctctctctctctctctctctctctctACTTGGAAAACAGGTCTCTCATAATA | 74 | G>C | Forward |
| 0.17 | M78snF | ctctctctctctctctctctctctctctctctctctctctctctCAAATTGATACACTTAACAAAGATACTTCTTTC | 77 | C>T | Forward |
| 0.17 | M9snR | tctctctctctctctctctctctctctctctctctctctctctctGTTTGAACATGTCTAAATTAAAGAAAAATAAAGAG | 80 | G>C | Reverse |
| 0.13 | M207snR | ctctctctctctctctctctctctctctctctctctctctctctctctctGAAGATTATTCAAAAGGTATTGTTATTCTCTTT | 83 | T>C | Reverse |
| 0.21 | M216snF | tctctctctctctctctctctctctctctctctctctctctctctctctctAGCTAGAAAAAAATTCCTTTATTAAAGAAATGTAA | 86 | C>T | Forward |

| **Additional NRY singleplex PCR primer information.** | | | | | | |
| --- | --- | --- | --- | --- | --- | --- |
| Name | Sequence 5'-3' | Name | Sequence 5'-3' | size in bp | Haplogroup | SNP name |
| M285F | TCATTTCTCATCATCTACATTTCTCC | M285R | TCGAATCCGCTATCCAGACT | 85 | G1 | M285 |
| P287F | CCTCTGGAACCTCCTGACTG | P287R | TTGGCAAAGCTAAAGCCACT | 72 | G2 | P287 |
| S126F | TTCAGAGCCCTTAGTAGCTCA | S126R | TTGGAAATTTAGGCAAGACCA | 85 | G2a3 | S126/L30 |
| M69F | TGGGTAGCCTGTTCAAATCC | M69R | TTCCCTTTGTCTTGCTGAAA | 82 | H | M69 |

**Table S7. Continued.**
